# Modeling Magnetic Fields from a DC Power Cable Buried Beneath San Francisco Bay Based on Empirical Measurements

**DOI:** 10.1371/journal.pone.0148543

**Published:** 2016-02-25

**Authors:** Robert Kavet, Megan T. Wyman, A. Peter Klimley

**Affiliations:** 1 Environment Sector, Electric Power Research Institute, Palo Alto, California, United States of America; 2 Biotelemetry Laboratory, Department of Fish, Wildlife, & Conservation Biology, University of California Davis, Davis, California, United States of America; Universidad Miguel Hernandez de Elche, SPAIN

## Abstract

The Trans Bay Cable (TBC) is a ±200-kilovolt (kV), 400 MW 85-km long High Voltage Direct Current (DC) buried transmission line linking Pittsburg, CA with San Francisco, CA (SF) beneath the San Francisco Estuary. The TBC runs parallel to the migratory route of various marine species, including green sturgeon, Chinook salmon, and steelhead trout. In July and August 2014, an extensive series of magnetic field measurements were taken using a pair of submerged Geometrics magnetometers towed behind a survey vessel in four locations in the San Francisco estuary along profiles that cross the cable’s path; these included the San Francisco-Oakland Bay Bridge (BB), the Richmond-San Rafael Bridge (RSR), the Benicia-Martinez Bridge (Ben) and an area in San Pablo Bay (SP) in which a bridge is not present. In this paper, we apply basic formulas that ideally describe the magnetic field from a DC cable summed vectorially with the background geomagnetic field (in the absence of other sources that would perturb the ambient field) to derive characteristics of the cable that are otherwise not immediately observable. Magnetic field profiles from measurements taken along 170 survey lines were inspected visually for evidence of a distinct pattern representing the presence of the cable. Many profiles were dominated by field distortions unrelated to the cable caused by bridge structures or other submerged objects, and the cable’s contribution to the field was not detectable. BB, with 40 of the survey lines, did not yield usable data for these reasons. The unrelated anomalies could be up to 100 times greater than those from the cable. In total, discernible magnetic field profiles measured from 76 survey lines were regressed against the equations, representing eight days of measurement. The modeled field anomalies due to the cable (the difference between the maximum and minimum field along the survey line at the cable crossing) were virtually identical to the measured values. The modeling yielded a pooled cable depth below the bay floor of 2.06 m (±1.46 std dev), and estimated the angle to the horizontal of the imaginary line connecting the cross-sectional center of the cable’s two conductors (0.1143 m apart) as 178.9° ±61.9° (std dev) for Ben, 78.6°±37.0° (std dev) for RSR, and 139.9°±27.4° (std dev) for SP. The mean of the eight daily average currents derived from the regressions was 986 ±185 amperes (A) (std dev), as compared to 722 ±95 A (std dev) provided by Trans Bay Cable LLC. Overall, the regressions based on fundamental principles (Biot Savart law) and the vectorial summation of cable and geomagnetic fields provide estimates of cable characteristics consistent with plausible expectations.

## Introduction

**Fig 1:**
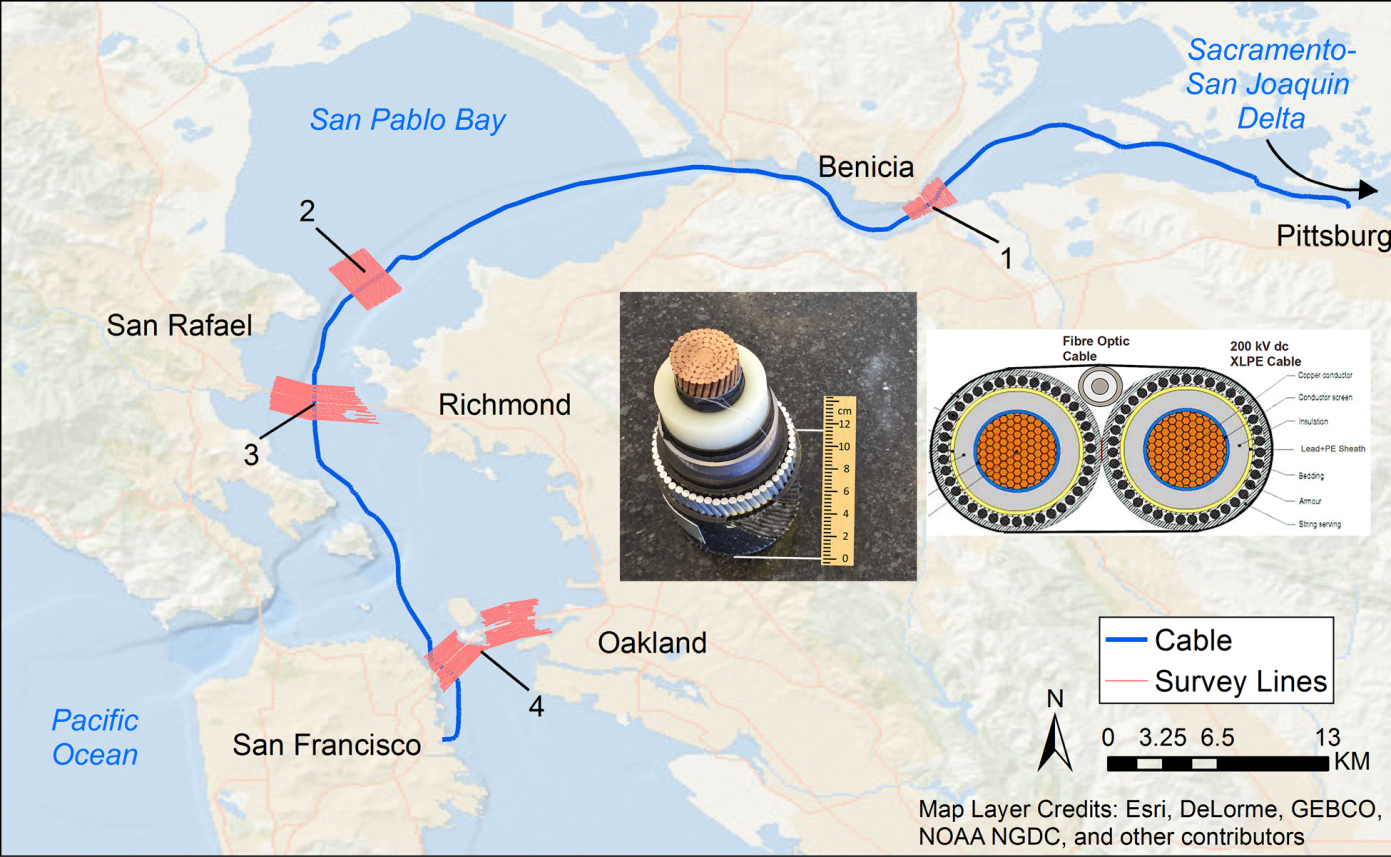
The Trans Bay Cable route (thick blue line) from Pittsburg, CA (upper right) to San Francisco, CA (lower left) showing locations of magnetometer surveys (red lines). (1) Benicia-Martinez Bridge (Ben), (2) San Pablo Bay (SP), (3) Richmond-San Rafael Bridge (RSR), and (4) San Francisco-Oakland Bay Bridge (BB). The migration route of anadromous fish runs between the Sacramento-San Joaquin Delta (upper right) and the mouth of the Pacific Ocean (lower left). The left inset shows a cross-section of the cable with a total diameter of 11.43 cm (note it is clad in a metallic sheath, which shields electric fields). The right inset shows a schematic of the cable bundle (source: TBC LLC).

The Trans Bay Cable (TBC) is a ±200-kilovolt (kV) 85-km long High Voltage Direct Current (DC) buried transmission line linking Pittsburg, CA with San Francisco, CA (SF) beneath the San Francisco Estuary (SFE) (Fig 1). The cable operates at a nominal power of 400 megawatts (MW); thus, the cable is rated to operate with an approximate nominal load current of 1,000 ampere (A). The current produces a DC magnetic field in the proximity of the cable; however, the cable is clad in a conductive sheath that shields the electric field the cable would otherwise produce (Fig 1, left inset). With the expected rise of offshore electric power generation with associated transport of electricity through buried cables such as these, there is heightened interest in assessing how anthropogenic magnetic fields may impact the behaviors of species with magnetosensitivity [1]. The TBC runs parallel or perpendicular (depending on the location) to the migratory route of various anadromous species that spend most of their adult life in the ocean, but migrate upstream to spawn in rivers. Adult Chinook salmon *Oncorhynchus tshawytscha*, steelhead trout *Oncorhynchus mykiss* [2] and green sturgeon *Acipenser medirostris* [3, 4] migrate from the Pacific Ocean through the San Francisco Bay to their spawning destinations further upstream in the Sacramento-San Joaquin Watershed. After spawning, steelhead trout and green sturgeon transit back through the bay on their way to their feeding grounds in the ocean and along the coast. Juvenile Chinook salmon *Oncorhynchus tshawytscha* [5, 6] and steelhead trout [7, 8] also migrate from the upstream rivers to the ocean. Studies have indicated that species within the salmonid [9–12] and sturgeon families [13–15] may use the natural ambient magnetic (or electric) fields to guide their movements during migration or foraging behaviors. Under an award from the U.S. Department of Energy researchers at the Electric Power Research Institute in Palo Alto, CA and the University of California at Davis are addressing whether the presence of the TBC affects migratory patterns of these anadromous species as they pass through the (SFE). In the current context, the cable is serving as a surrogate for assessing potential magnetic field effects associated with marine hydrokinetic technologies.

In July and August 2014, an extensive series of magnetic field measurements were taken by a pair of submerged Geometrics magnetometers towed behind a vessel in four locations in the SFE along survey lines that cross the cable’s path (Fig 1). In this paper, we apply basic formulas that ideally describe the magnetic field from a DC cable summed vectorially with the background geomagnetic field (in the absence of other sources that would perturb the ambient field) to derive characteristics of the cable that are otherwise not immediately observable. A consistency of the measured with the derived profiles would provide assurance that magnetic fields could be predicted for a specific set of cable conditions and configurations relevant to a previous date and location of interest with respect to fish migratory behavior.

## Methods

### DOE Approval

The protocol for this study, including the magnetometer survey, was reviewed and approved of by the US Department of Energy (DOE) and the US Bureau of Ocean Energy Management. Specially, the DOE approved a National Environmental Policy Act (NEPA) categorical exclusion determination for the in-water survey activities described in our proposal. The following is an excerpt from their approval letter:

“Upon review of the proposed activities and the biological evaluation, DOE has determined that the proposed in-water activities would:

- have no effect to Endangered Species Act listed species or critical habitat within the proposed action area
- not adversely affect essential fish habitat and fishery resources protected by the Magnuson-Stevens Fishery Conservation and Management Act within the proposed action area, and
- not result in the incidental harassment or taking of marine mammals protected by the Marine Mammal Protection Act within the proposed action area.

DOE’s determination of no effect was based on the type of survey activities proposed, the short in-water activity periods, the nature of survey equipment that would be used, and the proposed use of best management practices for boating. DOE has complied with the requirements under the Marine Mammal Protection Act, the Magnuson-Stevens Fishery Conservation and Management Act, and the Endangered Species Act, and the recipient is authorized to initiate in-water activities as described in Task 2 of the Statement of Project Objectives.”

### Study Site and Data Collection

The four locations were the San Francisco-Oakland Bay Bridge (BB), the Richmond-San Rafael Bridge (RSR) and the Benicia-Martinez Bridge (Ben), as well as an area in San Pablo Bay (SP) in which a bridge is not present (Fig 1). Survey lines were designed to run parallel to pre-existing cross-channel arrays of fish detecting acoustic hydrophones deployed in the San Francisco Bay along the three bridges and in San Pablo Bay. Most of these survey lines crossed the cable approximately perpendicularly with subsequent analyses limited to those with 90°±12.8° to limit possible error to ±2.5%. These lines extended along the entire span of the bridges (as far towards the banks as possible) and at least 1 km outwards from the fish detection array in San Pablo Bay. Survey lines started as close to the bridge/array as possible on each side and were repeated every 100 m away from the bridge/array up to 1 km. Twenty to 24 survey lines were delineated at each location.

To acquire magnetic field data, a G-882 Transverse Gradiometer (TVG) (Geometrics, Inc., San Jose, CA)—consisting of a pair of cesium magnetometers (sensitivity of 0.004 nT/√Hz RMS, with a 10-Hz sampling rate) separated by 1.5 m, two depth sensors, and an altimeter—was deployed into the water and towed behind the survey vessel. MagLog magnetic data acquisition software (Geometrics, Inc.) was used to design the survey line tracks, accurately navigate the boat, and integrate the data streams during field measurements.

[Note: A pair of magnetometers was used to increase the area sampled during each survey line transect. This was a more efficient and cost effective means of measuring magnetic fields over large areas. The readings from the two units were averaged for this study, but were essentially identical in all cases screened.]

The magnetic field measurements were geo-referenced using a Trimble GeoExplorer XT Global Positioning System (GPS) with Hurricane LI antenna (Trimble Navigation Limited, Sunnyvale, CA). Two types of tows were conducted on each survey line: 'Surface tows', with the TVG towed between 0.5 and 3 m beneath the water’s surface, were conducted along the full length of each survey line; and 'Deep tows' were conducted in areas where the TVG during Surface tows was more than 10 m above the channel bottom.

**Fig 2:**
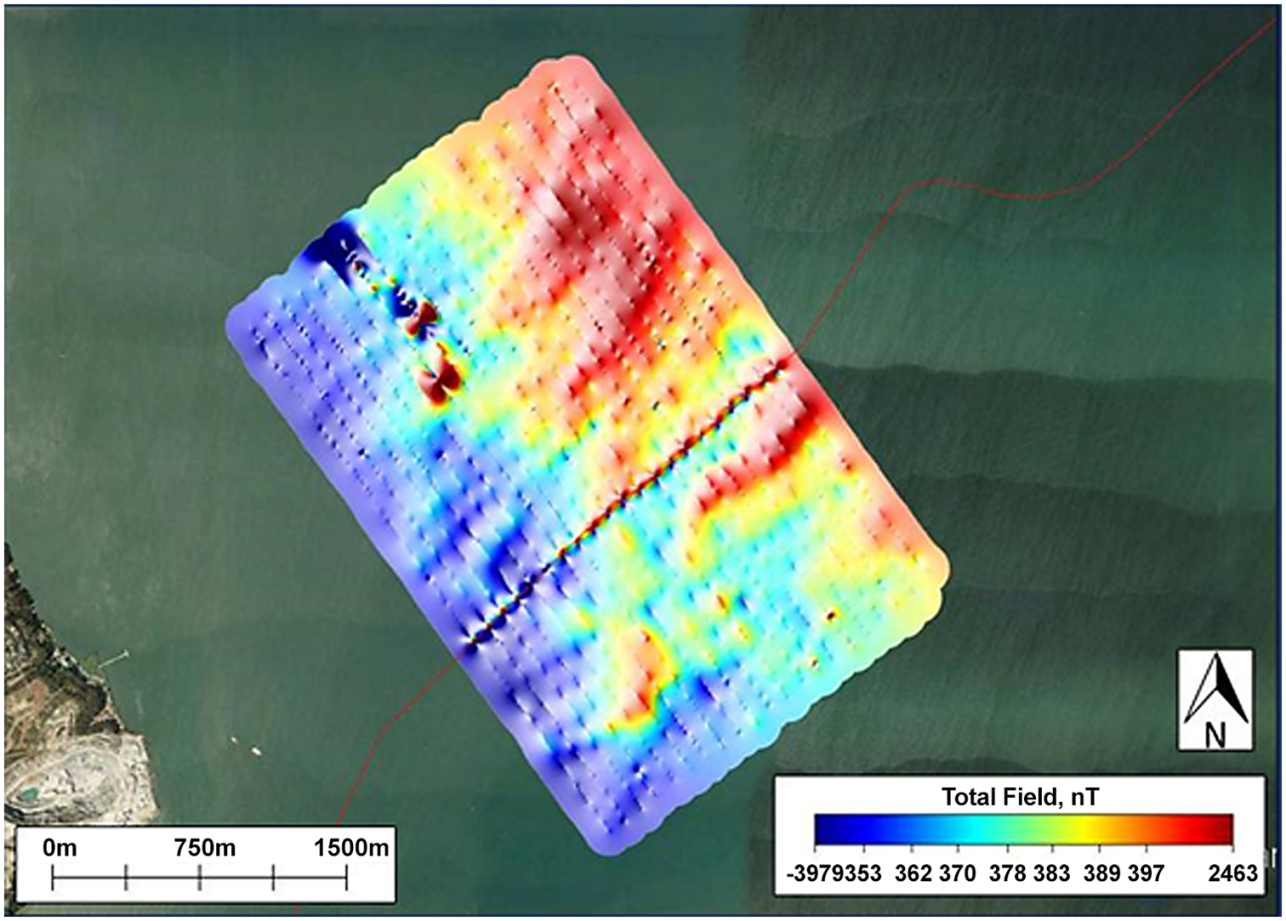
Local magnetic field map of San Pablo Bay generated by Surface tow profiles. The path of the cable, indicated by the line of small spikes in nT, can be seen running through the middle of the magnetic field map in a southwesterly direction.

After the field surveys were completed, MagPick magnetometer data processing software (Geometrics, Inc., http://sourceforge.net/projects/magpick/) was used to post-process and map the magnetic field data. Post-processing included 1) differentially correcting the GPS points to get submeter accuracy on survey positions using Trimble GPS Pathfinder Office software (Trimble Navigation Ltd.) (see note below), 2) correcting for tidal stage, based on tidal stage data downloaded from the nearest port station operated by the National Oceanic and Atmospheric Administration (NOAA), 3) correcting for fluctuations in the Earth's magnetic field (by subtracting daily measurements of the Earth's field made by the total-field magnetic field observatory at Jasper Ridge Biological Preserve from our field measures), and 4) removing HVDC offset between the two magnetometers on the TVG. After post-processing, the survey line profiles were used to map local magnetic field anomalies at each location for both the Surface and the Deep tows (Fig 2).

[Note: For many applications, it is very common practice to post-process GPS locations using differential corrections to reduce errors and obtain more accurate sub-meter positions. Although it is possible to differentially correct positions in real-time while taking the data points in the field, the GPS unit was not set up for this purpose, thus requiring the differential corrections to be performed at a later time during post-processing using Trimble GPS Pathfinder Office software.

### Magnetic Field Calculation

The magnetic fields attributable to the load on the TBC can be calculated with the Biot-Savart law (Appendix I), and are broken down to vertical, east and north components, which sum as vectors with the respective component of the geomagnetic field. In the SFE, the field has a total magnitude of about 48,800 nT, with a vertical component (i.e., perpendicular to the Earth’s plane) of about 42,800 nT, pointing into the Earth (down); a component pointing east with a magnitude of about 5,600 nT; and a component pointing north with a magnitude of about 22,700 nT. The subscripts in the following notation indicate the magnetic field (*B*) source, *Geo* for the geomagnetic field, *Cable* for the TBC, with each followed by their directional component, *North*, *East* or *Vertical*. The magnitude of the geomagnetic field, also known as its *resultant*, *B*_*GeoTotal*_, is calculated as,

$$B_{GeoTotal}=\;{\left[{\left(B_{GeoNorth}\right)}^{2}+\;{\left(B_{GeoEast}\right)}^{2}+\;{\left(B_{GeoVertical}\right)}^{2}\right]}^{0.5}\text{nT}$$ (1)

The combined *Geo* and *Cable* magnetic field resultant, *B*_*Total*_, is,

$$B_{Total}=\;{\left[{\left(B_{CableNorth}+B_{GeoNorth}\right)}^{2}+\;{\left(B_{CableEast}+B_{GeoEast}\right)}^{2}+\;{\left(B_{CableVertical}+B_{GeoVertical}\right)}^{2}\right]}^{0.5}\text{nT}$$ (2)

The *Net*, that is, the amount that the magnitude of the local field near the TBC deviates from the magnitude of the geomagnetic field is,

$$B_{Net}=B_{Total}-B_{GeoTotal}\;\text{nT}$$ (3)

**Fig 3:**
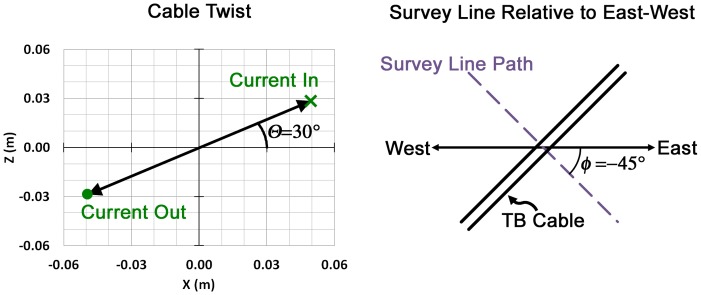
Depiction of the angle of cable twist (*ϴ*), and the angle of the lateral survey line relative to geographic East-West (*Ф*).

The equations that serve as the basis for the cable fields in eq 2 are provided in Appendix II. The parameters used to describe the magnetic field attributable to the TBC using the Biot-Savart law are listed in Table 1, with a description of *ϴ* and *Ф* shown in Fig 3.

**Table 1:** Parameters for computing field from DC cable.

Parameter	Symbol	Units
Current	*I*	Amps (A)
Conductor separation	*s*	meters (m)
Height of magnetometer above bottom	ALT	m
Buried depth of cable	*a*	m
Lateral distance of magnetometer along the survey line from vertical projection of cable	*x*	m
Cable twist (Fig 4)	*ϴ*	radians (degrees)
Profile angle relative to East-West (Fig 4)	*Ф*	radians (degrees)
North component of cable field	*B*_*CableNorth*_	nT
East component of cable field	*B*_*CableEast*_	nT
Vertical component of cable field	*B*_*CableVertical*_	nT
North component of geomagnetic field	*B*_*GeoNorth*_	nT
East component of geomagnetic field	*B*_*GeoEast*_	nT
Vertical component of geomagnetic field	*B*_*GeoVertical*_	nT

**Fig 4:**
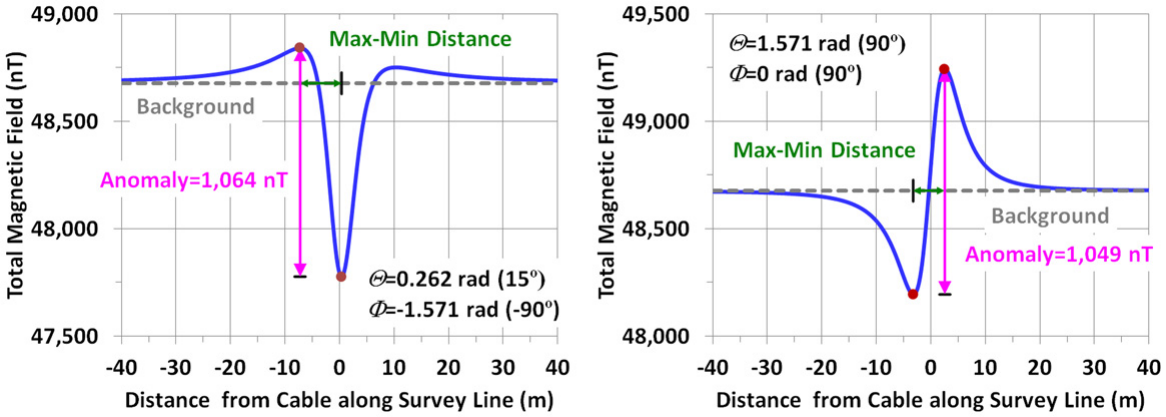
The total magnetic field for two sample profiles (5 m above the cable, loaded to 1,000 A). The graphs indicate the magnitude of the anomaly and the distance between field minima and maxima. The anomaly's magnitude is measured as the difference between the maxima and minima of the total magnetic field values. The examples illustrate the variability of the profiles with different values of *ϴ* and *Ф*.

The centers of the two cross-sections of the conductors that comprise the cable are 0.1143 meters (m) apart (4.5 inches) (*s* in Table 1 and see right inset in Fig 1). Two examples of the dependence of the field profile on *ϴ* and *Ф* are illustrated in Fig 4; the examples assume the following values (and assumes the survey line’s path is exactly perpendicular to the cable):

I= 1,000 A

s= 0.1143 m

$$\begin{array}{l} \text{ALT}+a=\;5\text{ m }\left(\text{that is, the total vertical height to the measurement point from the cable}\right)\\ B_{GeoTotal}=\;48,677\text{ nT }\left(\text{horizontal broken line}\right) \end{array}$$

The cable ‘*Anomaly*’ is the perturbation in the ambient magnetic field produced by the cable. Its magnitude is the difference between the maximum and the minimum net magnetic field along the survey line at the cable crossing. Note that for these examples, meant to represent nominal load conditions, the field decays to background by ~±40 m lateral distance from the vertical projection of the center of the cable.

### Fitting the Observed Data

For each magnetometer (1 and 2), the post-processed data for each measurement point included:

- Survey line profile number
- Latitude and longitude (LAT1/LAT2 and LONG1/LONG2) (degrees)
- z1 and z2, the magnetometers’ depth beneath the surface (m)
- ALT1 and ALT2, the magnetometers’ height above bottom (m)
- T1 and T2, the local net magnetic field (nT) (i.e., after subtracting background earth field)
- A single date and time stamp for each pair of data points

For each point the LAT1 and LAT2, LONG1 and LONG2, z1 and z2, ALT1 and ALT2, and T1 and T2 were averaged (the paired values were almost always virtually identical). The distance along the survey line referenced to the first point was determined by transforming the difference between the latitude and longitude of the first point with each successive measurement point into an interval (m) using the haversine formula (with 6,371 km used as an estimate of the Earth’s radius), and transforming each latitude-longitude pair into a cumulative distance (see Appendix III).

For each field profile with a clear anomaly above the cable crossing through visual inspection, a segment anywhere between 400 and about 2,500 consecutive records was extracted for further processing. A first order approximation of the point designated *x* = 0 m (approximate center of cable) was assigned as the point along the survey line corresponding to the average of the distances of the maximum and the minimum fields within the anomaly. Also as a first order approximation, the background magnetic field (i.e., not attributable to the cable) was ‘zeroed’ by subtracting the crude average field across the profile from each measurement point. Then, to perform the regression, the field profile was trimmed to a segment with the anomaly signature that ranged between 128 m to 300 m. This approach excluded irregular field patterns beyond either side of these segments to allow a ‘cleaner’ regression of the anomaly.

By convention, the observed and modeled profiles were drawn with the left side of the x-axis representing the eastern (or southeastern) end of each profile. Having this convention means the currents will not change polarity (and the angles will not flip) as one ‘switches back’ between profiles of successive survey lines. The curve fittings were performed on the non-linear regression platform of JMP, Version 5.0.1. The distance from the magnetometer to the bottom, ALT, was regressed with every measurement point to derive *a*, cable depth. The components of the Earth’s field for the measurement locales were obtained from the NOAA website, http://www.ngdc.noaa.gov/geomag-web/#igrfwmm. In addition to *I*, *ϴ* and *a*, the regression used two fitting parameters (not shown) to transpose the fitted curve horizontally (x, distance from center of cable) and vertically (B, the magnetic field) to match the observed anomaly as closely as possible. The value of *Ф*, based on the known direction of each measurement profile relative to East-West, was entered into the regression as an initial empirically-based angle, and fine-tuned subsequently by fixing I, *ϴ*,and a, and allowing *Ф* to ‘adjust’ to the best fit, given those values.

Magnetic field profiles that were distorted or obscured by structures or submerged objects or possibly other electrical activity in the vicinity were not analyzed in the regression model. Nor were profiles regressed when the sine of the angle between a survey line’s path and the cable heading was less than 0.975 (<77.2° or >102.8°), as the model assumes a perpendicular relationship between the two.

Two further factors are noted with regard to the cable’s physical properties. The first concerns the cable’s twist angle. The cable consists of two conductors with a cross-section photo of one shown in the left inset of Fig 1. The right inset in the figure illustrates that the two conductors are bundled. According to a 2014 TBC presentation (source: http://www.ewh.ieee.org/r6/san_francisco/pes/pes_pdf/TransBayCable2014.pdf), the cable is buried roughly 6 feet (1.83 m) beneath the bay floor, and given its linear path where the measurements occurred (see below) and relatively rigid structure, we consider the twist angle to be constant along the relevant cable segment for each field profile. As the cable bends along its path, the twist angle may rotate at specific points or gradually, but this cannot be known definitively given the cable is buried out of sight beneath the water.

The second concerns the cable’s curvature (or lack thereof). The site maps used for the measurements indicated that, for the survey lines with discernible profiles (*i*.*e*., those reported under Results), the cable’s path was linear where the survey vessel crossed that path. If the cable had significant curvature, then the simplified version of the Biot Savart equation (based on an infinitely linear cable) would not provide accurate results. Of the 76 magnetic field profiles analyzed, one was within 25–30 m of a 30° ‘dogleg’ of the cable, and this case, dealt with further in the Discussion, did not significantly skew the results.

## Results

### The Sample

The results presented below include measurements taken at three locations on eight separate days in 2014 from July 10 to August 7. Two groups of measurements were taken at each location, one closer to the surface (S), and another at a greater depth (D); thus, for example, the Deep measurements at San Pablo Bay would be identified by SP-D. These measurements were taken along the same set of parallel survey lines at each location. A single set of survey lines were run at each location except for Richmond Bridge. Due to a curvature in this bridge, three sets of parallel survey lines were required to accurately measure the magnetic fields in this location. The cable’s path runs perpendicularly through the middle of the central set of survey lines at Richmond Bridge and so only measurements from this set of lines were used in the subsequent analysis below. The breakdown of survey line profiles by location and date is shown in Table 2, indicating the number of profiles measured and the number with sufficient structure at the anomaly to be regressed. Anomalies unrelated to the DC cable could be up to roughly 100 times greater than the anomaly associated with the cable. Only two of the 40 profiles at BB were regressed, and the BB profiles were not considered further in the results. For the other three locations, 76 out of a total of 130 measured profiles were fitted (58.5%). The raw data for all 130 are provided in Tables A-F in S1 File.

**Table 2:** Survey Line Profiles: Dates Measured and Number Regressed.

Group	Date	Number of Profiles Measured	Number of Profiles Regressed	Percent
**BB-D**	**8/6**	**2**	**0**	**0.0%**
**BB-D**	**8/8**	**16**	**2**	**12.5%**
**BB-D**	**8/9**	**2**	**0**	**0.0%**
**BB-S**	**7/16**	**9**	**0**	**0.0%**
**BB-S**	**7/17**	**9**	**0**	**0.0%**
**BB-S**	**7/25**	**2**	**0**	**0.0%**
Ben-D	8/7	24	7	29.2%
Ben-S	8/7	24	9	37.5%
RSR-D	8/1	11	7	63.6%
RSR-D	8/4	9	7	77.8%
RSR-S	7/10	10	4	40.0%
RSR-S	7/15	10	7	70.0%
SP-D	7/29	2	2	100.0%
SP-D	7/31	19	18	94.7%
SP-S	7/28	13	10	76.9%
SP-S	7/29	8	5	62.5%
	All	170	78	45.9%
	w/o BB	130	76	58.5%

BB, San Francisco-Oakland Bay Bridge; Ben, Benicia-Martinez Bridge; RSR, Richmond-San Rafael Bridge; SP, San Pablo Bay. Suffixes represent measurement depth: D, Deep; S, Surface. (Bolded rows not included in Results because of insufficient sample size.)

**Fig 5:**
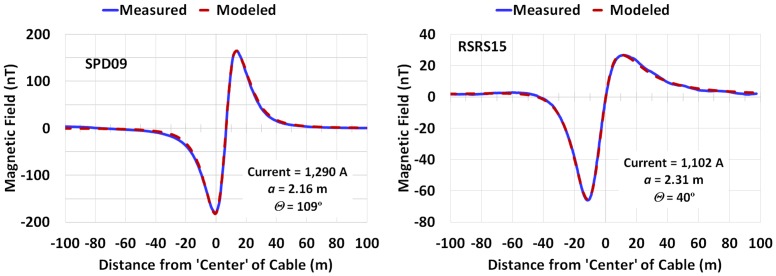
Two examples of the measured and modeled net magnetic field (*B*_*net*_). The derived values of current (*I*), cable depth (*a*), and twist angle (*ϴ*) are shown in the insets. (SPD09, San Pablo Bay Deep, Profile #9;RSRS15, Richmond San Rafael bridge Surface, Profile #15).

With the exception of Ben-S, whose profiles analyzed included between 401 and 601 data points across an average distance of 144 m, the other sites had profiles consisting of between roughly 800 and 2,500 data points across distances of 166 m to 300 m (Table 3). The average distance between measurement points for each group (organized by location and tow depth) ranged from between 0.15 m/measurement to 0.26 m/measurement with the minima and maxima shown in Table 3. The average height of the magnetometers above the bottom of the water, ALT_avg_ (m), pooled across all three locations was 8.00±1.07 m for Deep and 13.72±1.56 m for Surface measurements, with no statistically significant differences across locations (one-way ANOVA; *p*>0.2 for Deep, p>0.9 for Surface). Two examples of the curve-fitting results for profiles displaying a discernible anomaly are shown in Fig 5. In general all of the fits were similar in nature to those shown in the max-min region of the curves, but several fits diverged at the right and/or left tail (probably attributable to unrelated, but unidentifiable sources). The observed and modeled profiles for all 76 magnetic field profiles evaluated are provided in Figs A-BX in S2 File.

**Table 3:** Summary Characteristics of Survey Line Profiles Regressed.

Group	N Profiles	N Meas.	Profile Distance (m)	Dist/Meas (m)
Ben-D	7	1492 (1393–1601)*	217 (166–250)	0.15 (0.10–0.17)
Ben-S	9	423 (401–601)	144 (128–202)	0.34 (0.32–0.38)
RSR-D	14	1368 (1256–1489)	228 (199–300)	0.17 (0.13–0.20)
RSR-S	11	1026 (790–1263)	258 (200–300)	0.26 (0.19–0.38)
SP-D	20	1611 (887–2474	260 (200–300)	0.17 (0.08–0.23)
SP-S	15	1215 (962–1474)	271 (200–300)	0.23 (0.15–0.31)
All	76	1252 (401–2474)	238 (128–300)	0.21 (0.08–0.38)

* Mean (Min-Max Range)

Twist Angle, *ϴ*, and Cable Depth, *a*

### Anomaly

**Fig 6:**
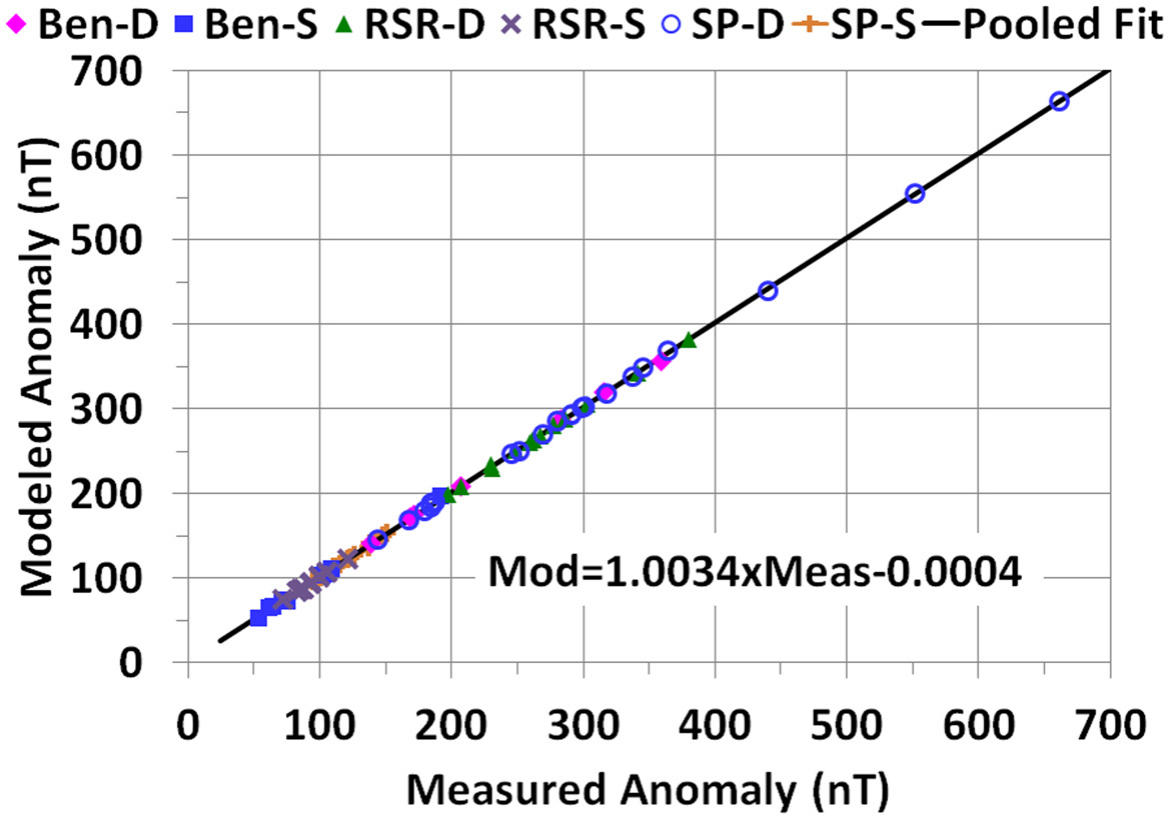
Modeled versus measured anomaly by data set group. The regression line represents all points pooled.

**Fig 7:**
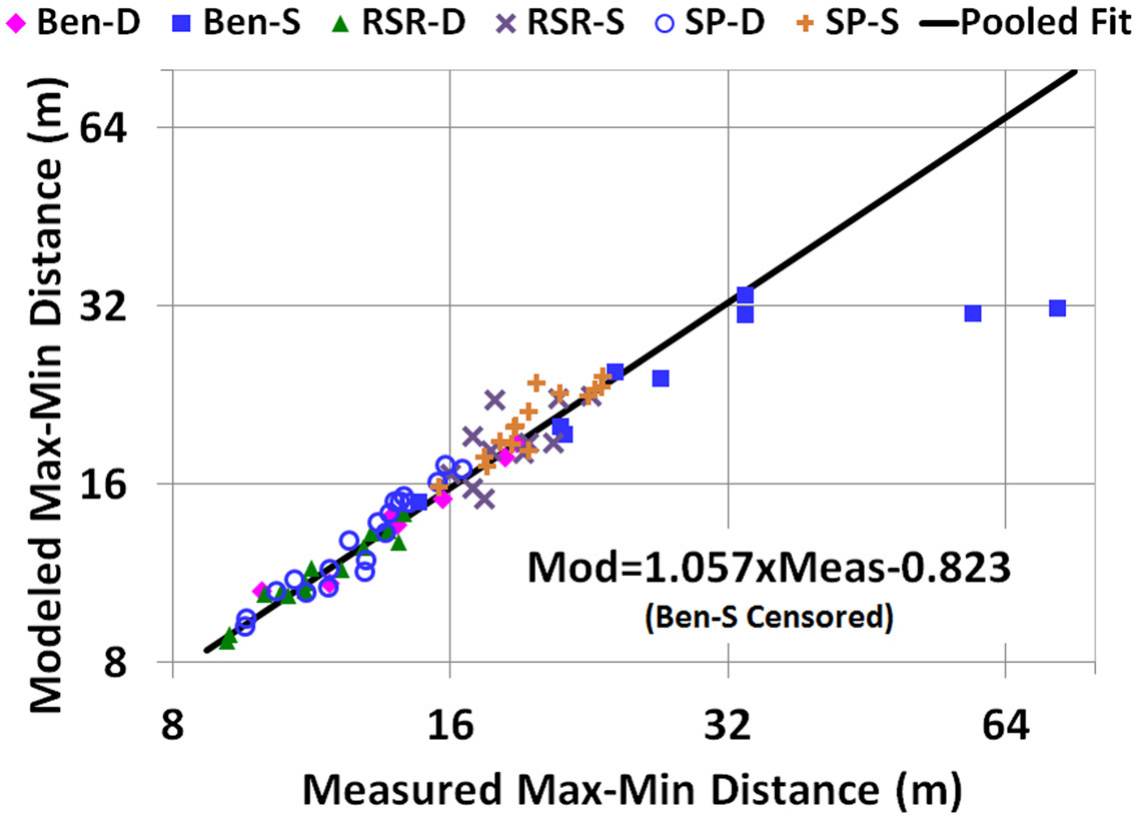
Modeled versus measured absolute min-max distance by data set group. The regression line represents all points pooled with the exception of Ben-S (see Discussion).

A primary result of the exercise is that the measured and modeled values of the anomalies were virtually identical, with a pooled slope of unity (Pearson correlation, *r* = 0.9999) when plotted against each other and a pooled intercept consistent with the origin (Fig 6). Ben-S was censored for comparing the modeled values of max-min distance (Fig 7) to the measured values because their profiles were notably shorter than those from the other groups (Table 3 and Figs A-BX in S2 File), and the observed anomalies did not appear to have well defined minima (dealt with further in Discussion). For the other five groups the modeled absolute max-min distances were highly correlated to the observed absolute values (*r* = 0.97), and the average of the modeled values (15.43±4.32 m) was virtually identical to the average of the measured values (15.37±3.96 m). The *t*-test comparing the slope in Fig 7 to 1.0 was not statistically significant (*p*>0.05, df = 65), and the intercept of the regression line was consistent with the origin (*p*>0.10).

Besides the separation of the cable’s two conductors (0.1143 m for its entire route), the other two physical descriptors of the cable that are relevant to the magnetic field, but are not observable in any practical way, are its twist angle, *ϴ* (deg) and buried depth, *a* (m). The descriptive statistics summarizing the regression results are shown in Tables 4 and 5.

**Table 4:** Modeled Mean Twist Angle, *ϴ* (deg), by Location and Depth of Measurement.

	Deep (1)		Surface (0)		Pooled	
Location	N	Mean ±sd	N	Mean ±sd	N	Mean ±sd
Ben	7	160.8±74.5	9	192.9±50.2	16	178.9±61.9
RSR	14	80.2±33.1	11	76.6±43.0	25	78.6±37.0
SP	20	139.8±27.3	15	140.0±28.5	35	139.9±27.4

**Table 5:** Modeled Mean Cable Depth, *a* (m), by Location and Measurement Depth.

	Deep (1)		Surface (0)		Pooled	
Location	N	Mean ±sd	N	Mean ±sd	N	Mean ±sd
Ben	7	1.39±1.02	9	3.53±2.33	16	2.59±2.12
RSR	14	1.57±0.64	11	1.11±1.45	25	1.37±1.07
SP	20	2.19±1.01	15	2.48±1.33	35	2.31±1.15

The derived mean values of these variables for each profile were entered into a model in which the three locations were categorical variables (‘Location’) and Measurement Depth was assigned a dummy variable (Deep = 1; Surface = 0), and an interaction term, Location*Depth was included.

The ANOVA model for *ϴ* was highly statistically significant with *p* = 0.0003 (*F* = 5.4 df 5/70), with the significance attributable to differences across locations (p = 0.0003). *ϴ* was not significantly related to measurement depth (i.e., within Location) (*p*>0.2), and the interaction term was also not statistically significant (p>0.3). The pooled values of *ϴ* by Location were 178.9°±61.9° for Ben, 78.6°±37.0° for RSR, and 139.9°±27.4° for SP; note the relatively greater variability for Ben.

The ANOVA model for *a* was statistically significant (*p* = 0.0012; *F* = 4.5 df 5/70), with the significance attributable mainly to differences across locations (*p* = 0.0004), and the interaction term (*p* = 0.01). The statistical significance of the interaction is most apparent in the disparate Deep and Surface values for Ben (Table 5). Cable depth, *a*, derived from the model was marginally related to measurement depth (i.e., within Location) (*p* = 0.043), with the estimate of cable depth 0.66 m lower for Deep than for Surface measurements. The pooled values of *a* by Location were 2.59±2.12 m for Ben, 1.37±1.07 m for RSR, and 2.31±1.15 m for SP. Were one to assume that the cable maintained a constant depth along its entire route as an engineering specification, then the mean cable depth, *a*, pooled across all 76 profiles was 2.06 m (standard deviation, sd = 1.46 m).

### Current

**Fig 8:**
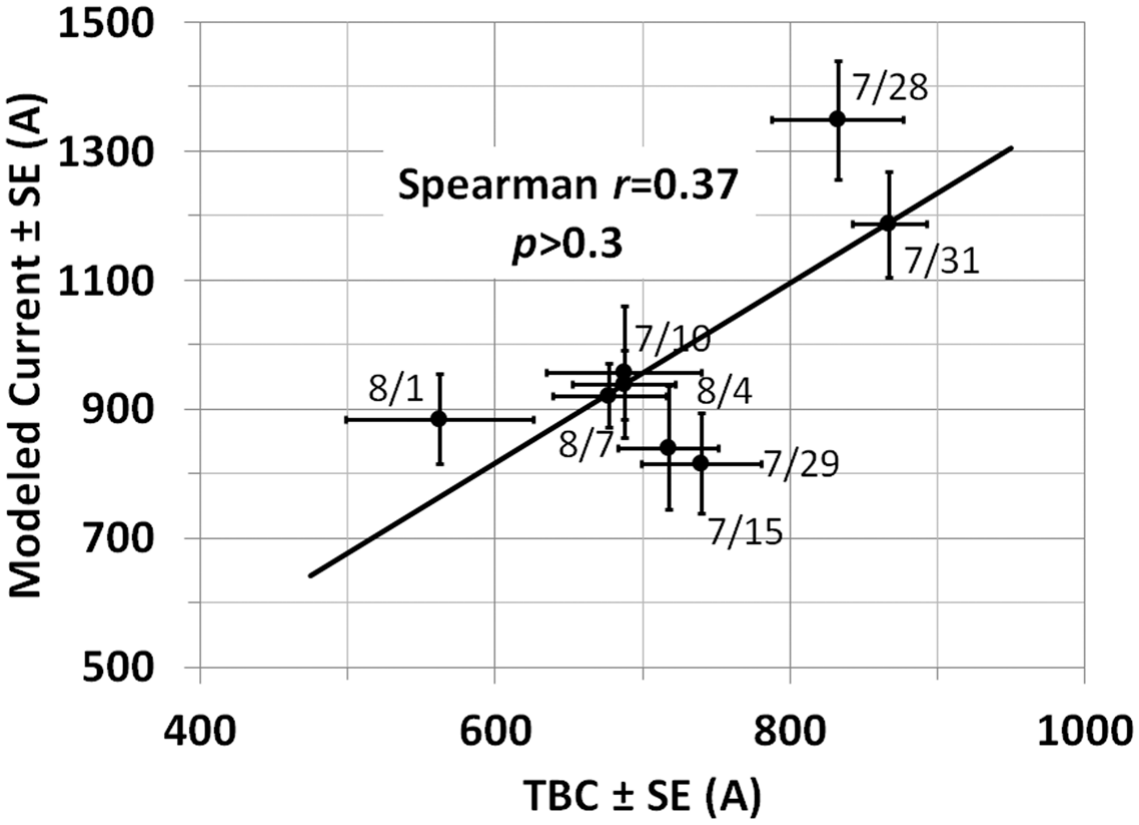
Average modeled current versus average current provided by TBC for each of the eight days for which data are reported. The least-square regression line is shown. Note SE for TBC is based on the assumption that the variance provided was based on 24 hourly samples; for the survey described here, SE is based on number of survey lines for each day.

Trans Bay Cable LLC provided daily average load±sd expressed in MW, and assuming a constant voltage of ±200 kV, current was calculated as watts/(4*10^5^ volts). For TBC, the daily means over the eight measurement days (Table 2) averaged 722 A ±95 A std dev and the modeled values averaged 986 A ±185 A std dev, resulting in a difference of 264±134 A with a paired two-tailed *t*-test with *p*<0.001 (*t* = 5.57, df = 7). The eight paired mean values yielded a Spearman correlation coefficient of 0.37 (*p*>0.3) (and a Pearson value of 0.72, *p* = 0.045) (Fig 8 shows the least-squares regression line). The difference in the two correlation values is attributable to the small sample size. All 76 derived currents were lognormally distributed by the Shapiro-Wilk test with a geometric mean of 981 A, and a geometric standard deviation of 1.34 (95% range of 918-1,048 A).

## Discussion

The mean values of twist angle, *ϴ*, and cable depth, *a*, derived from the regressions appear to bear a consistency with what one could expect from direct observation of these parameters. However, the values for current in many cases (36 of 76) were greater than those expected a priori, given the cables nominal rating of 1,000 A. The values derived from regressing any single profile would not be expected to be a reliable estimate of the cable’s characteristics, but the objective of growing the sample size as large as possible is to have errors even out on both the positive and negative side.

The ambient DC magnetic field detected by the magnetometers included all sources of DC magnetic fields and all perturbations of the background magnetic field, whether caused by large objects, other electrical activity, or by field variations associated with ferromagnetic deposits in the upper earth’s crust. Thus, while a distinct pattern of an anomaly associated with the cable could be visually discernible, we cannot assume the field pattern was pristine, that is, due to only the cable and the geomagnetic field posted on the NOAA website for the location of interest. The modeling also accepted profiles whose angle relative to the cable’s direction were 90°±12.8°, a decision that introduces an error of up to 2.5% in the calculated cumulative distance from the first measurement point of a survey line’s field profile, although the error was typically much less (data not shown). The trade-off for enforcing a tighter tolerance would have been a loss of data.

### Detectability of Anomalies

The magnetometers appeared to be exquisitely sensitive to the DC magnetic field environment in all locations surveyed. The manufacturer specifies a nominal resolution of 3 nT, and that sensitivity was abundantly apparent as they detected anomalies from the cable as small as about 50 nT. Detection of cable-associated anomalies was clearly handicapped by the presence of bridge structures. At the BB, only 2 of 40 (20 Deep, 20 Surface) profiles had detectable anomalies, which manifested as ‘blips’ on a profile mostly obscured by the bridge and other unidentified objects. In addition, the magnetometers recorded evidence of the activity of the Bay Area Rapid Transit (BART) system—which operates at 1,000 V DC—at the edge of various BB profiles although distant from the cable crossing of the survey lines.

At Ben, 7 of 24 (29.2%) Deep profiles and 9 of 24 (37.5%) Surface profiles could be analyzed in the regression model, all but one of these (a Surface profile) on the southwest side of the bridge. Anomalies for the profiles immediately adjacent to the bridge and all those northeast (save one) were not visually resolvable. The most obvious explanation for the latter would concern unidentified submerged objects with magnetic properties located on the northeast side of the bridge.

RSR provided a very distinct pattern of readable as opposed to non-readable anomalies. A total of 20 profiles were measured for both Deep and Surface, 10 south of the bridge and 10 to the north. The three profiles immediately to the north and south were not readable, although one could see a distortion of the anomaly in several of these. All of the other Deep profiles, 14 of 20 (70%), were regressed and 11 of 20 (55%) Surface profiles were regressed.

No bridges were located in SP, affording an opportunity to measure in relatively ‘open water’ resulting in resolving nearly all of the Deep profiles (20/21, 95.2%) and the majority of the Surface profiles (15/21, 71.4%). There may have been other objects in SP that perturbed the Surface measurements, they having weaker fields from the cable than Deep measurements, while the fields at the deeper measurement locations may have surmounted any perturbances.

**Fig 9:**
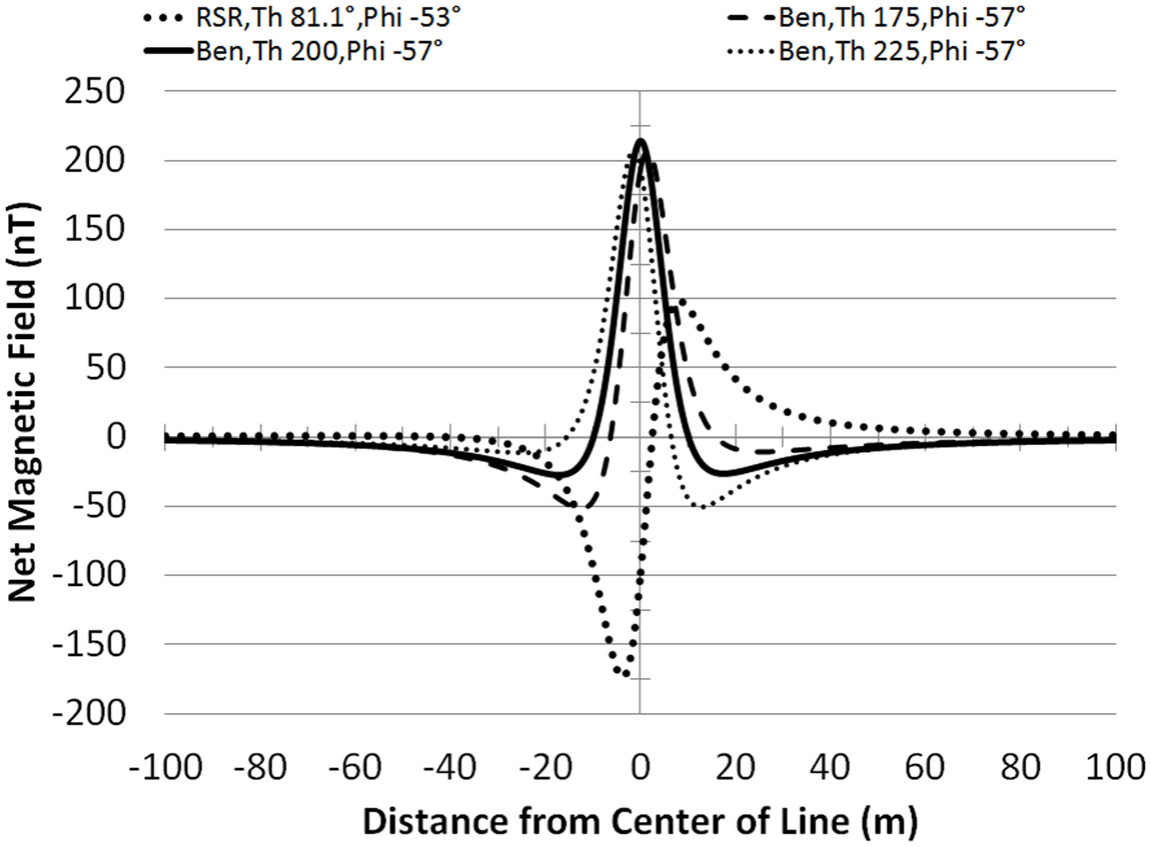
Variability of profile pattern structure. An illustration of how the profile has a less distinct pattern for *ϴ* and *Ф* in the Benicia-Martinez Bridge (Ben) data set centered on *ϴ* ≈200° (thick black curve), compared to the more distinct ‘whiplash’ pattern for *ϴ* and *Ф* in the Richmond San Rafael Bridge (RSR). The Ben curves for *ϴ* = 175° and *ϴ* = 225° illustrate transitional patterns with a more distinct structure than for *ϴ* = 225°.

Compared to RSR and SP, Ben had the greatest variability in *ϴ* and *a*, (Tables 4 and 5) and the regressed profiles at Ben-S did not produce estimates of min-max distances consistent with the values based on the measurements (although Ben-D did) (Fig 8). One plausible reason for the large variability is that the estimate of *ϴ* (192.9°±50.2°) for Ben-S includes 200°, which has a much less distinct anomaly pattern than the more typical ‘whiplash’ shape with a distinct structure lending it comparatively more amenable to an accurate regression. The imprecision of estimating min-max differences for Ben-S was not related entirely to a lack of sensitivity attributable to having the lowest anomaly of all eight groups (93.5±42.4 nT). For RSR-S, with nearly the same average anomaly (second lowest with 95.7±13.4 nT), but with a ‘whiplash’ field contour, the estimated min-max differences were consistent with the measured values. These factors are illustrated in Fig 9 with curves based on idealized calculations (in the same manner as conducted for Fig 4). They show the unimodal pattern of the anomaly with *ϴ* = 200° (and *Ф* = -57°), characteristic of Ben-S along with the slightly more distinct shapes at 175° and 225°. By comparison, RSR-S had an estimated *ϴ* of slightly lower variability of 76.6°±43.0° (with *Ф* = -53°), which produces more of the whiplash shape shown in Fig 9 that apparently resolves comparatively better in the regression exercise.

### Anomaly

Regardless of these sources of error, the values of the parameters produced by the regression produced an excellent fit with the observed anomalies with their magnitudes virtually identical. The distance between the modeled minima and maxima averaged over the entire sample were also virtually identical to the average of the measured values. The slope of the modeled to measured regression line was consistent statistically with unity and the intercept was consistent statistically with the origin.

**Fig 10:**
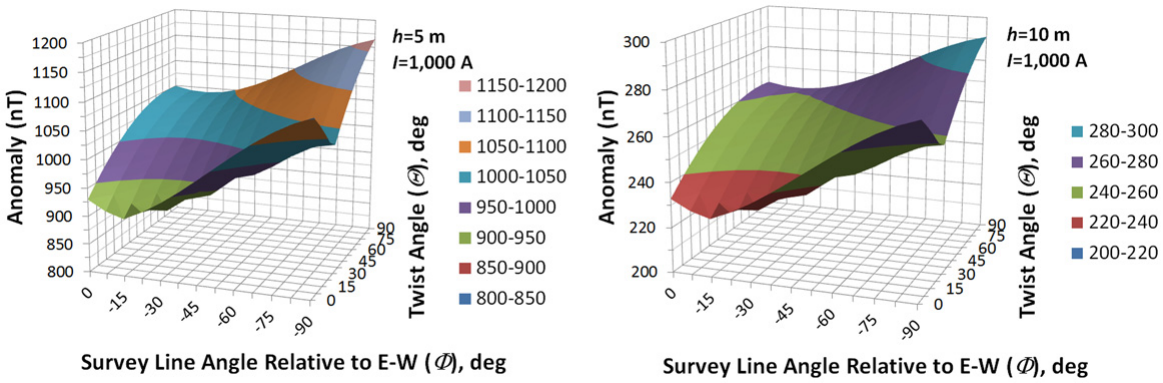
Sensitivity of the anomaly to height above the cable, as well as to *ϴ* and *Ф*. See text.

For a cable such as the TBC, the magnitude of the anomaly scales directly with current and decreases as the inverse square of the distance above the cable. However, there remains a dependency also on *ϴ* and *Ф*. The 3-D contour charts in Fig 10 illustrate how the anomaly’s magnitude decreases by a factor of 4 when the height, *h*, above the cable doubles (the contour retains the same shape with respect to *ϴ* and *Ф* at both heights). The variability across *ϴ* and *Ф* should be evident in the contours, with a max:min ratio of about 1.3.

### Twist Angle, *ϴ*

At any location along its route, the cable’s twist angle, *ϴ*, has an effect on the magnitude of the anomaly associated with the load on the cable. For example, for a field profile 10 m above the cable loaded at 1,000 A, with survey line angle, *Ф* = -55° (similar to *Ф* at Ben and SP), the calculated anomaly can vary over a 14.3% range relative to the anomaly averaged over 360° (259 nT). Thus, to predict what the anomaly may be at any of the three locations, an accurate estimate of *ϴ* is desirable. Despite the high variability of the estimate of *ϴ* for Ben, the data suggest that the cable may twist at various points or gradually along its path between Pittsburg and San Francisco (Table 4). The high variability of the estimate of *ϴ* for Ben may also be based on a comparatively less distinct field profile at the Ben location (Discussion above and Fig 9).

### Cable Depth, *a*

Cable depth is a challenging parameter, because it represents only a portion of the vertical drop from the magnetometer to the cable itself, and the regression solves for total height with *a* the difference between height and ALT. For the Surface measurements (*N* = 35), the ratio of *a* to the average of ALT across each profile was 0.237±0.132, and for the Deep measurements (*N* = 41), the ratio was 0.175±0.147. Negative values of *a* were derived in 2 of 35 Surface measurements and 1 of 41 Deep measurements (not a statistically significant difference by Fischer’s exact 2-tail test, *p*>0.5); of course, a negative value is implausible as it represents the cable as above the water’s bottom surface. Understanding cable depth is important from the perspective of estimating anomalies. Using the example of 1,000 A, and 10-m measurement height above the cable, the average anomaly over all possible values of *ϴ* in 7.5° increments and *Ф* from 0° to -90° in 7.5° increments is 259 nT, with a slope of -52 nT per meter increment above the cable. Thus, with regard to calculating an anomaly at a particular point, errors in estimating *a* can be at least as sizable as errors associated with estimating *ϴ*. Nevertheless, the mean pooled value of *a* derived from all 76 profiles was 2.06 m, with a 95% range of 1.73-2.39 m, which straddles the value of 6 feet or 1.83 m cited in the 2014 TBC presentation referenced previously.

### Current

Load currents were derived from magnetic field profiles measured during daytime hours on eight days in July and August 2014. As described in Results, the sample of 76 current values was lognormally distributed with a 95% range that encompasses the cable’s rated current. The averages of these derived values for each day were compared to 24-hour average loads for the same days furnished by TBC LLC. Thus, the two data sets, though plotted against each other (Fig 8), were based on different time frames.

Of greater potential concern is that for 36 of the 76 (47%) profiles reported, the derived currents exceeded the nominal load rating of the line (1,000 A). A portion, if not all, of these exceedances can be attributed to variability inherent in the regression model. It is not known to the investigators whether some of these were due to periods of the day in which TBC LLC was asked by the system operator to increase load beyond the rated level to satisfy demand for electricity. The 13 highest derived currents all occurred for SPS on 7/28/14 (6 profiles) and SPD on 7/31/14 (7 profiles), the same two days TBC reported their highest daily averages. Therefore, it is no coincidence that the regressions here derived the highest current values for those same days, and the high values are almost certainly unrelated to location. Also, those two days had only moderate temperatures in San Francisco, with maxima of 74° F (23.3°C) and 72°F (22.2°C) on 7/28 and 7/31, respectively (http://www.wunderground.com/history/airport/KSFO/2014/1/2/DailyHistory.html). It is more likely that the high loads for those days were related to demand factors unrelated to weather.

### Curvature of the Cable

**Fig 11:**
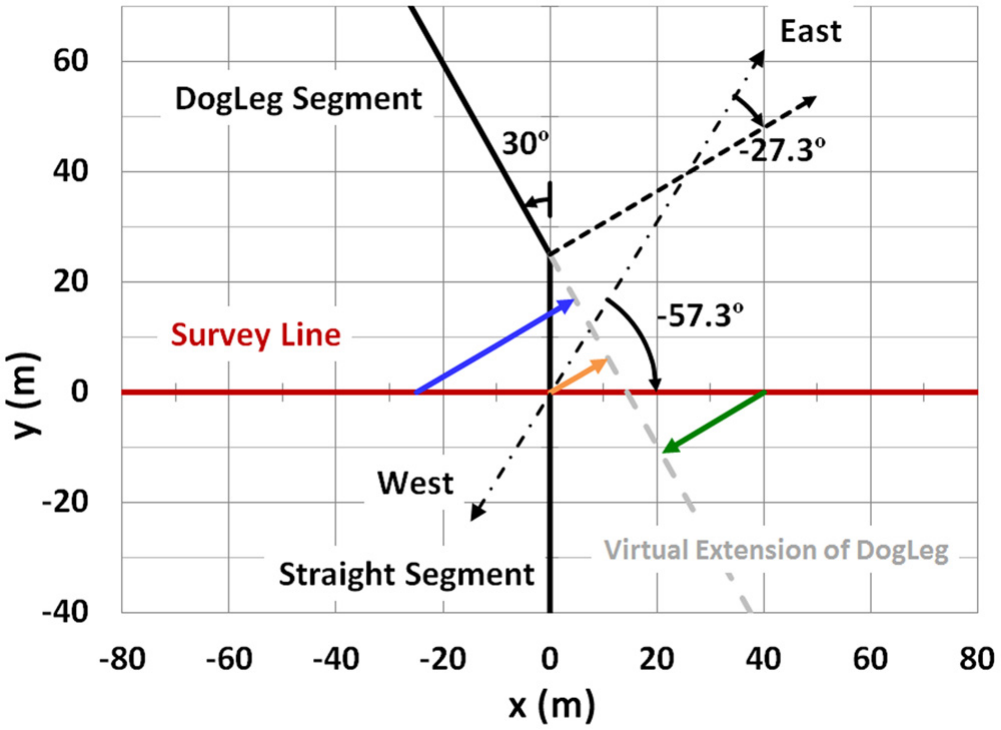
Scenario for evaluating the effect of a 30° dogleg 25 m from the survey line for magnetic field profile BenS16. The drawing illustrates the case that was analyzed by Biot Savart to estimate the effect of the dogleg on the magnetic field profile. The colored lines with arrowheads represent the perpendicular projection of various points along the survey line to the virtual extension of the dogleg segment of the cable. Also represented are the angle between the survey line (red) and East-West (dash-dot black line), and the angle between the projection of the dogleg extension (dashed black line) with East-West.

**Fig 12:**
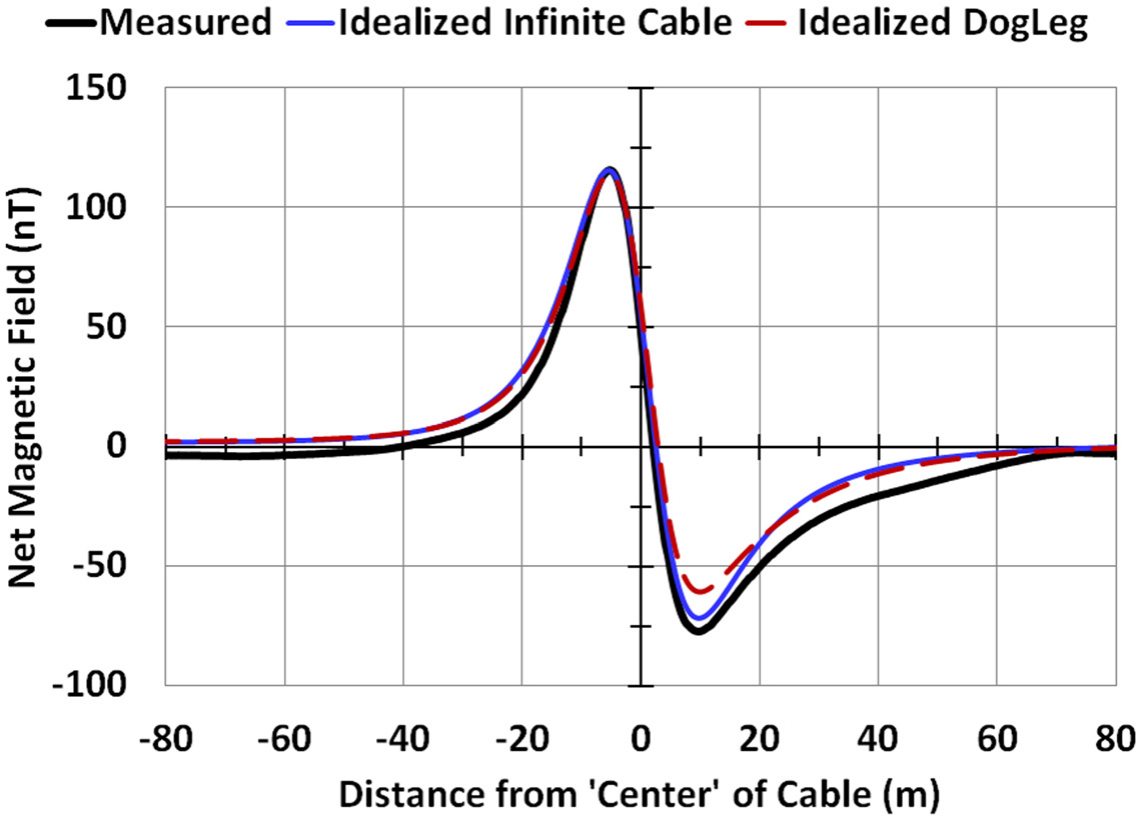
Comparison of idealized profiles from an infinitely straight cable with the dogleg scenario shown in Fig 11. The modeled magnetic field profile for an infinitely straight cable and the dogleg scenario shown in Fig 11. The values of the parameters entered were: *I* = 1,123 A; average measurement height above the cable, measurement height, *h* = *a*+ALT_avg_ = 12.9; *ϴ* = 269°; and *Ф* = -57.3° for the straight segment and *Ф* = -27.3° for the dogleg segment.

In one of the 76 cases analyzed, BenS16, the survey line crossed the cable within 25 m of a 30° ‘dogleg’ in the cable’s path. However, the regressions performed in this study assumed the cable was infinitely straight in all cases. The Biot Savart equations were applied for this case to estimate the error associated with a straight cable assumption. The cable was split into two segments, each extending to infinity from the point of the dogleg. Then the North, East and Vertical contributions from each segment were summed respectively with the geomagnetic field components, and *B*_*net*_ was calculated (eqs 1–3 and Appendix II). The model assumed values of the parameters that resulted from the infinitely straight cable regression. The scenario is illustrated schematically in Fig 11 (see caption, which includes values of the parameters entered). The results, shown in Fig 12, indicate that theoretically, including the dogleg would produce a 7% smaller anomaly compared to the infinitely straight line case, and a min-max distance 2% lower; the figure includes the original observation for this survey line. For the nine profiles analyzed for BenS, averaging over the sample would result ideally in a correction of 1.4 nT, and 0.038 m for min-max distance. Given that the variability associated with the measurement data can be considerable, this minor difference is not considered of significance.

## Conclusion

The magnetometer used in the survey of magnetic fields in the San Francisco Bay faithfully recorded the ambient DC magnetic fields in its pathway. Otherwise, the regressions of the visible anomalies could not have plausibly produced the matches as exemplified in Fig 5. This study has demonstrated that the regression formulas applied to the measured profiles based on the fundamental principle of the Biot-Savart law and the vectorial summation of cable and geomagnetic fields in rectangular coordinates provide estimates of cable characteristics consistent with reasonable expectations. Nonetheless, various factors contribute to variability inherent in such an effort, particularly uncontrollable sources of field perturbations that do not lend themselves to modeling, and remain empirical observations. Whether magnetic field perturbations of a large magnitude from fixed structures and objects clustered in individual locations contribute to behavioral modifications in fish, as opposed to relatively small anomalies on the order of <1,000 nT continuously present along an 85 km long DC cable route is the subject of ongoing and possibly future research. Regardless, the methods described in this paper should be applicable to other buried HVDC cables. However, they would not apply to buried alternating current (AC) power cables for which modeling would be more complex.

### Appendix I: Biot Savart Equation

**Fig 13:**
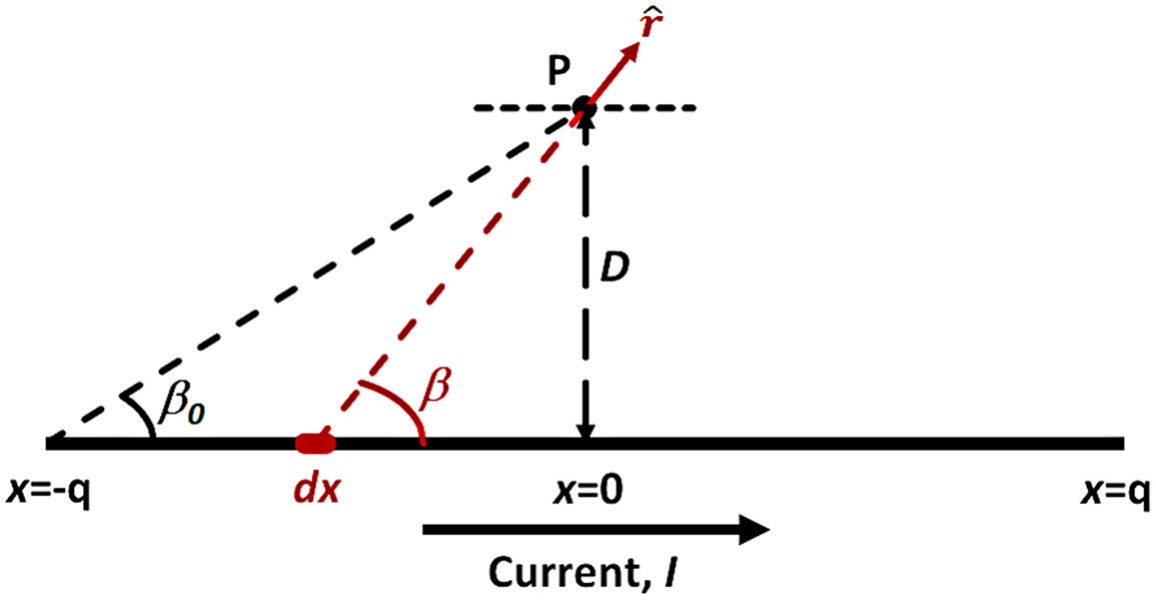
Parameters used for the Biot Savart calculation.

Assume a straight conductor carrying current, *I* (Fig 13). At a point, P, a perpendicular distance, *D*, from the conductor, the magnetic flux density, *B*, attributable to the conductor segment a distance ±*q* from the intersection of the perpendicular projection with the conductor is:

$$B\left(P\right)=\left(\mu_{0}/4\pi )I\int_{-q}^{q}(d\vec{x}\times \hat{r}\right)/r^{2}$$ (4)

For a straight conductor, vector notation is not necessary for calculating the magnitude of *B*.

$$\left|d\vec{x}\times \hat{r}\right|=dx*sin\left(\beta \right)$$ (5)

$$r^{2}=x^{2}+D^{2}$$ (6)

$$sin\left(\beta \right)=D/\sqrt{x^{2}+D^{2}}$$ (7)

$$\mu_{0}=4\pi *10^{-7}$$ (8)

Integrating,

$$B\left(P\right)=\text{cos}\left(\beta_{0}\right)\left(2I\times 10^{-7}\right)/D\;\text{Tesla}$$ (9)

$$B\left(P\right)=\text{cos}\left(\beta_{0}\right)\left(2I\times 10^{2}\right)/D\;\text{nT}$$ (10)

$$\text{If }q\gg D,\text{ cos}\left(\beta_{0}\right)\to 1.0,\text{ and}$$

$$B\left(P\right)=2I\times 10^{2}/D\;\text{nT},\text{ the Biot Savart equation}.$$ (11)

The formula on which the Biot Savart equation is based indicates that for a point, P, a perpendicular distance of *D* meters away from a conductor, 95% of the field at P is accounted for by the current in the conductor within ±3.04x*D* from the point of P’s perpendicular projection onto the conductor. To account for 97.5% of the field, the multiple is ±4.39, and for 99% it is ±7.02. Thus, for a measurement point 10 meters above the horizontal plane of the cable and 20 meters in the lateral direction, 95% of the measured field is accounted for in the segment ±68.0 meters from the perpendicular projection of P on the cable, 97.5% by ±98.1 meters, and 99% by ±157 meters.

### Appendix II: Magnetic Field Equations

Both the DC magnetic field from the cable and the geomagnetic field were broken into mutually orthogonal vectors in a Cartesian coordinate space, with the vectors pointing east, north and vertical (pointing into the earth). All parameters are identified in Table 1 of the main manuscript.

The magnetic fields due to the current on the cable are as follows:

$$\begin{array}{l} B_{CableNorth}=(I/5)*\text{SIN}(\Phi )*\{((h+a)-(s/2)*\text{SIN}(\theta ))/[{\left(x-(s/2)*\text{COS}(\theta )\right)}^{2}+((h+a)-\\ \left(s/2)*\text{SIN}(\theta ){)}^{2}]-((h+a)+(s/2)*\text{SIN}(\theta ))/[{\left(x+(s/2)*\text{COS}(\theta )\right)}^{2}+{\left((h+a)+(s/2)*\text{SIN}(\theta )\right)}^{2}]\right\} \end{array}$$ (12)

$$\begin{array}{l} B_{CableEast}=(I/5)*\text{COS}(\Phi )*\{((h+a)-(s/2)*\text{SIN}(\theta ))/[{\left(x-(s/2)*\text{COS}(\theta )\right)}^{2}+((h+a)-\\ \left(s/2)*\text{SIN}(\theta ){)}^{2}]-((h+a)+(s/2)*\text{SIN}(\theta ))/[{\left(x+(s/2)*\text{COS}(\theta )\right)}^{2}+{\left((h+a)+(s/2)*\text{SIN}(\theta )\right)}^{2}]\right\} \end{array}$$ (13)

$$\begin{array}{l} B_{CableVertical}=(I/5)*\{(x-(s/2)*\text{COS}(\theta ))/[{\left(x-(s/2)*\text{COS}(\theta )\right)}^{2}+((h+a)-\\ \left(s/2)*\text{SIN}(\theta ){)}^{2}]-(x+(s/2)*\text{COS}(\theta ))/[{\left(x+(s/2)*\text{COS}(\theta )\right)}^{2}+{\left((h+a)+(s/2)*\text{SIN}(\theta )\right]}^{2})\right\} \end{array}$$ (14)

Then, with the north, east and vertical components of the geomagnetic field defined as *B*_*GeoNorth*_, *B*_*GeoEast*_, and *B*_*GeoVertical*_, respectively, the magnitude of the total field, *B*_*Total*_, is:

$$B_{Total}=\;{\left[{\left(B_{CableNorth}+B_{GeoNorth}\right)}^{2}+{\left(B_{CableEast}+B_{GeoEast}\right)}^{2}+{\left(B_{CableVertical}+B_{GeoVertical}\right)}^{2}\right]}^{0.5}\;\;\;\;\;$$ (15)

The geomagnetic field, *B*_*GeoTotal*_ is calculated as:

$$B_{GeoTotal}=\;{\left(B_{GeoNorth}{}^{2}+B_{GeoEast}{}^{2}+B_{GeoVertical}{}^{2}\right)}^{0.5}$$ (16)

Finally, the ***anomaly*** itself is expressed as *B*_*Net*_, which is *B*_*GeoTotal*_ subtracted from *B*_*Total*_:

$$B_{Net}=B_{Total}-B_{GeoTotal}$$ (17)

**Fig 14:**
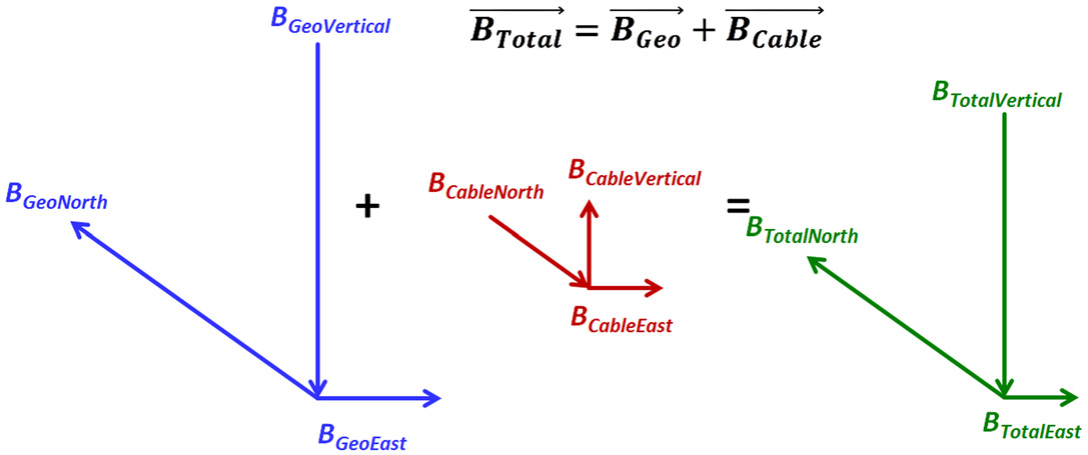
Vector relationships.

The summation of vector components from the cable and geomagnetic sources is shown in Fig 14.

### Appendix III: Haversine Equation

The circumferential distance between points 1 and 2 may be expressed by the haversine formula:

$$\begin{array}{l} D_{1-2}=\{2*6371*\text{ASIN}[\text{SQRT}\{(1-\text{COS}(2\pi *\text{LAT}2/360-2\pi *\text{LAT}1/360))/2+\text{COS}(2\pi *\text{LAT}1/360)*\\ \text{COS}(2\pi *\text{LAT}2/360)*(1-\text{COS}(2\pi *\text{LONG}2/360-2\pi *\text{LONG}1/360))/2\}]\}*1000\text{meters} \end{array}$$ (18)

where 6,371 km is an estimate of the Earth’s radius in kilometers and LAT1/LONG1 and LAT2/LONG2 refer to the coordinates of points 1 and 2. (For further description, see http://www.movable-type.co.uk/scripts/latlong.html but many other websites are available)

## Supporting Information

S1 File Raw Data. Table A. BenD, Raw Data. Table B. BenS, Raw Data. Table C. RSRD, Raw Data. Table D. RSRS, Raw Data. Table E1. SPD, Raw Data1. Table E2. SPD, Raw Data2. Table F1. SPS, Raw Data1. Table F2. SPS, Raw Data2.
(ZIP)

S2 File Measured and Modeled Profiles. Fig A. BenD01, Measured and Modeled Profiles. Fig B. BenD02, Measured and Modeled Profiles. Fig C. BenD03, Measured and Modeled Profiles. Fig D. BenD05, Measured and Modeled Profiles. Fig E. BenD06, Measured and Modeled Profiles. Fig F. BenD07, Measured and Modeled Profiles. Fig G. BenD09, Measured and Modeled Profiles. Fig H. BenS01, Measured and Modeled Profiles. Fig I. BenS02, Measured and Modeled Profiles. Fig J. BenS03, Measured and Modeled Profiles. Fig K. BenS04, Measured and Modeled Profiles. Fig L. BenS05, Measured and Modeled Profiles. Fig M. BenS08, Measured and Modeled Profiles. Fig N. BenS09, Measured and Modeled Profiles. Fig O. BenS10, Measured and Modeled Profiles. Fig P. BenS16, Measured and Modeled Profiles. Fig Q. RSRD01, Measured and Modeled Profiles. Fig R. RSRD02, Measured and Modeled Profiles. Fig S. RSRD03, Measured and Modeled Profiles. Fig T. RSRD04, Measured and Modeled Profiles. Fig U. RSRD05, Measured and Modeled Profiles. Fig V. RSRD06, Measured and Modeled Profiles. Fig W. RSRD07, Measured and Modeled Profiles. Fig X. RSRD14, Measured and Modeled Profiles. Fig Y. RSRD15, Measured and Modeled Profiles. Fig Z. RSRD16, Measured and Modeled Profiles. Fig AA. RSRD17, Measured and Modeled Profiles. Fig AB. RSRD18, Measured and Modeled Profiles. Fig AC. RSRD19, Measured and Modeled Profiles. Fig AD. RSRD20, Measured and Modeled Profiles. Fig AE. RSRS01, Measured and Modeled Profiles. Fig AF. RSRS02, Measured and Modeled Profiles. Fig AG. RSRS03, Measured and Modeled Profiles. Fig AH. RSRS05, Measured and Modeled Profiles. Fig AI. RSRS07, Measured and Modeled Profiles. Fig AJ. RSRS15, Measured and Modeled Profiles. Fig AK. RSRS16, Measured and Modeled Profiles. Fig AL. RSRS17, Measured and Modeled Profiles. Fig AM. RSRS18, Measured and Modeled Profiles. Fig AN. RSRS19, Measured and Modeled Profiles. Fig AO. RSRS20, Measured and Modeled Profiles. Fig AP. SPD01, Measured and Modeled Profiles. Fig AQ. SPD02, Measured and Modeled Profiles. Fig AR. SPD03, Measured and Modeled Profiles. Fig AS. SPD04, Measured and Modeled Profiles. Fig AT. SPD06, Measured and Modeled Profiles. Fig AU. SPD07, Measured and Modeled Profiles. Fig AV. SPD08, Measured and Modeled Profiles. Fig AW. SPD09, Measured and Modeled Profiles. Fig AX. SPD10, Measured and Modeled Profiles. Fig AY. SPD11, Measured and Modeled Profiles. Fig AZ. SPD12, Measured and Modeled Profiles. Fig BA. SPD13, Measured and Modeled Profiles. Fig BB. SPD14, Measured and Modeled Profiles. Fig BC. SPD15, Measured and Modeled Profiles. Fig BD. SPD16, Measured and Modeled Profiles. Fig BE. SPD17, Measured and Modeled Profiles. Fig BF. SPD18, Measured and Modeled Profiles. Fig BG. SPD19, Measured and Modeled Profiles. Fig BH. SPD20, Measured and Modeled Profiles. Fig BI. SPD21, Measured and Modeled Profiles. Fig BJ. SPS01, Measured and Modeled Profiles. Fig BK. SPS02, Measured and Modeled Profiles. Fig BL. SPS03, Measured and Modeled Profiles. Fig BM. SPS04, Measured and Modeled Profiles. Fig BN. SPS07, Measured and Modeled Profiles. Fig BO. SPS08, Measured and Modeled Profiles. Fig BP. SPS09, Measured and Modeled Profiles. Fig BQ. SPS10, Measured and Modeled Profiles. Fig BR. SPS12, Measured and Modeled Profiles. Fig BS. SPS13, Measured and Modeled Profiles. Fig BT. SPS14, Measured and Modeled Profiles. Fig BU. SPS15, Measured and Modeled Profiles. Fig BV. SPS17, Measured and Modeled Profiles. Fig BW. SPS18, Measured and Modeled Profiles. Fig BX. SPS19, Measured and Modeled Profiles.
(PPTX)
