# Supplementary material for: Modeling Magnetic Fields from a DC Power Cable Buried Beneath San Francisco Bay Based on Empirical Measurements
Source: PLoS One. 2016 Feb 25;11(2):e0148543. doi: 10.1371/journal.pone.0148543 (PMC4767330; doi:10.1371/journal.pone.0148543)
Supplement: S2 File — Fig A. BenD01, Measured and Modeled Profiles. Fig B. BenD02, Measured and Modeled Profiles. Fig C. BenD03, Measured and Modeled Profiles. Fig D. BenD05, Measured and Modeled Profiles. Fig E. BenD06, Measured and Modeled Profiles. Fig F. BenD07, Measured and Modeled Profiles. Fig G. BenD09, Measured and Modeled Profiles. Fig H. BenS01, Measured and Modeled Profiles. Fig I. BenS02, Measured and Modeled Profiles. Fig J. BenS03, Measured and Modeled Profiles. Fig K. BenS04, Measured and Modeled Profiles. Fig L. BenS05, Measured and Modeled Profiles. Fig M. BenS08, Measured and Modeled Profiles. Fig N. BenS09, Measured and Modeled Profiles. Fig O. BenS10, Measured and Modeled Profiles. Fig P. BenS16, Measured and Modeled Profiles. Fig Q. RSRD01, Measured and Modeled Profiles. Fig R. RSRD02, Measured and Modeled Profiles. Fig S. RSRD03, Measured and Modeled Profiles. Fig T. RSRD04, Measured and Modeled Profiles. Fig U. RSRD05, Measured and Modeled Profiles. Fig V. RSRD06, Measured and Modeled Profiles. Fig W. RSRD07, Measured and Modeled Profiles. Fig X. RSRD14, Measured and Modeled Profiles. Fig Y. RSRD15, Measured and Modeled Profiles. Fig Z. RSRD16, Measured and Modeled Profiles. Fig AA. RSRD17, Measured and Modeled Profiles. Fig AB. RSRD18, Measured and Modeled Profiles. Fig AC. RSRD19, Measured and Modeled Profiles. Fig AD. RSRD20, Measured and Modeled Profiles. Fig AE. RSRS01, Measured and Modeled Profiles. Fig AF. RSRS02, Measured and Modeled Profiles. Fig AG. RSRS03, Measured and Modeled Profiles. Fig AH. RSRS05, Measured and Modeled Profiles. Fig AI. RSRS07, Measured and Modeled Profiles. Fig AJ. RSRS15, Measured and Modeled Profiles. Fig AK. RSRS16, Measured and Modeled Profiles. Fig AL. RSRS17, Measured and Modeled Profiles. Fig AM. RSRS18, Measured and Modeled Profiles. Fig AN. RSRS19, Measured and Modeled Profiles. Fig AO. RSRS20, Measured and Modeled Profiles. Fig AP. SPD01, Measured and Modeled Profiles. Fig AQ. SPD02, Measured and Modeled Profiles. Fig AR. [file pone.0148543.s002.pptx]

## Slide 1
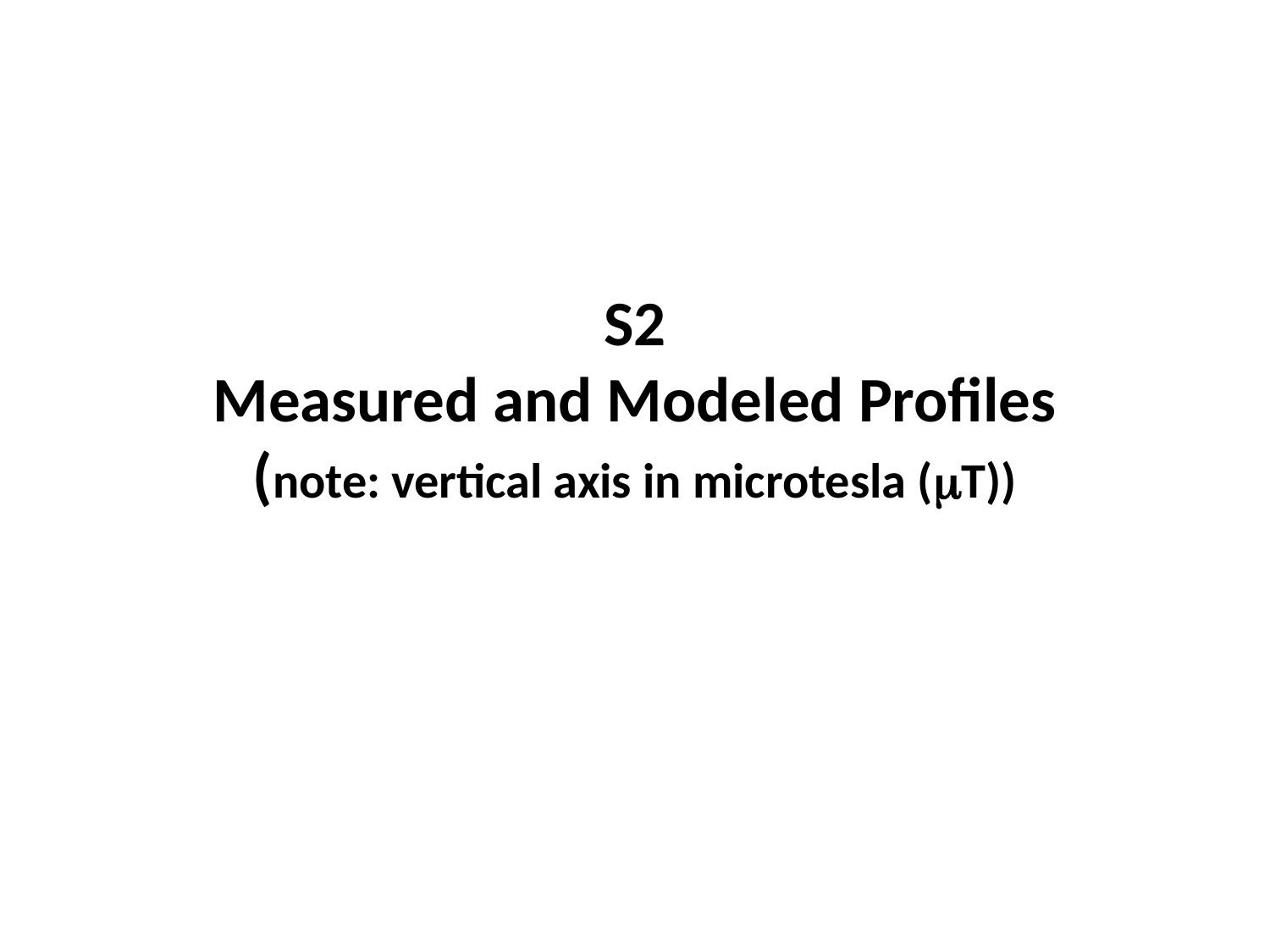

# S2 Measured and Modeled Profiles (note: vertical axis in microtesla (mT))

## Slide 2
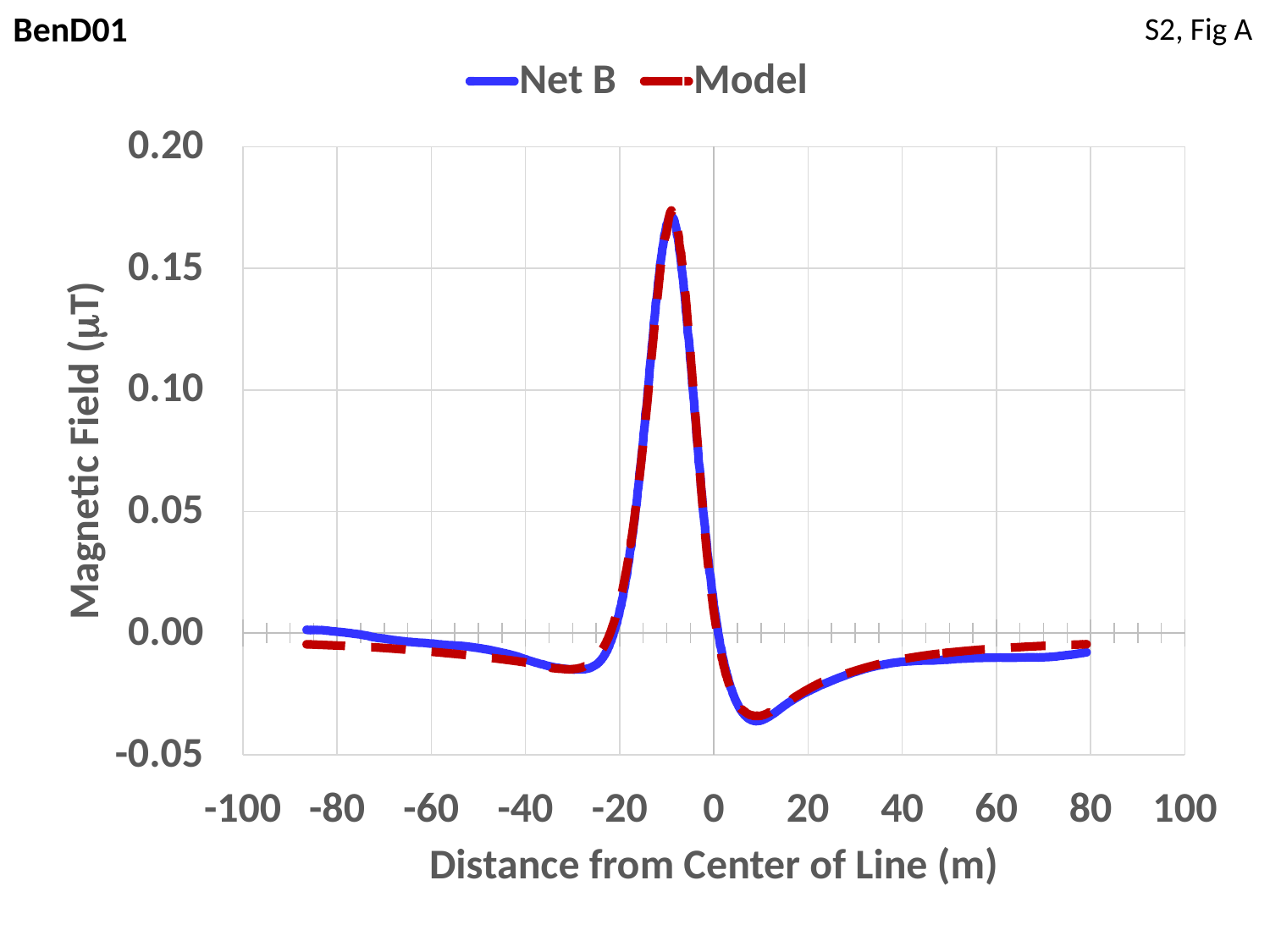

BenD01
S2, Fig A

## Slide 3
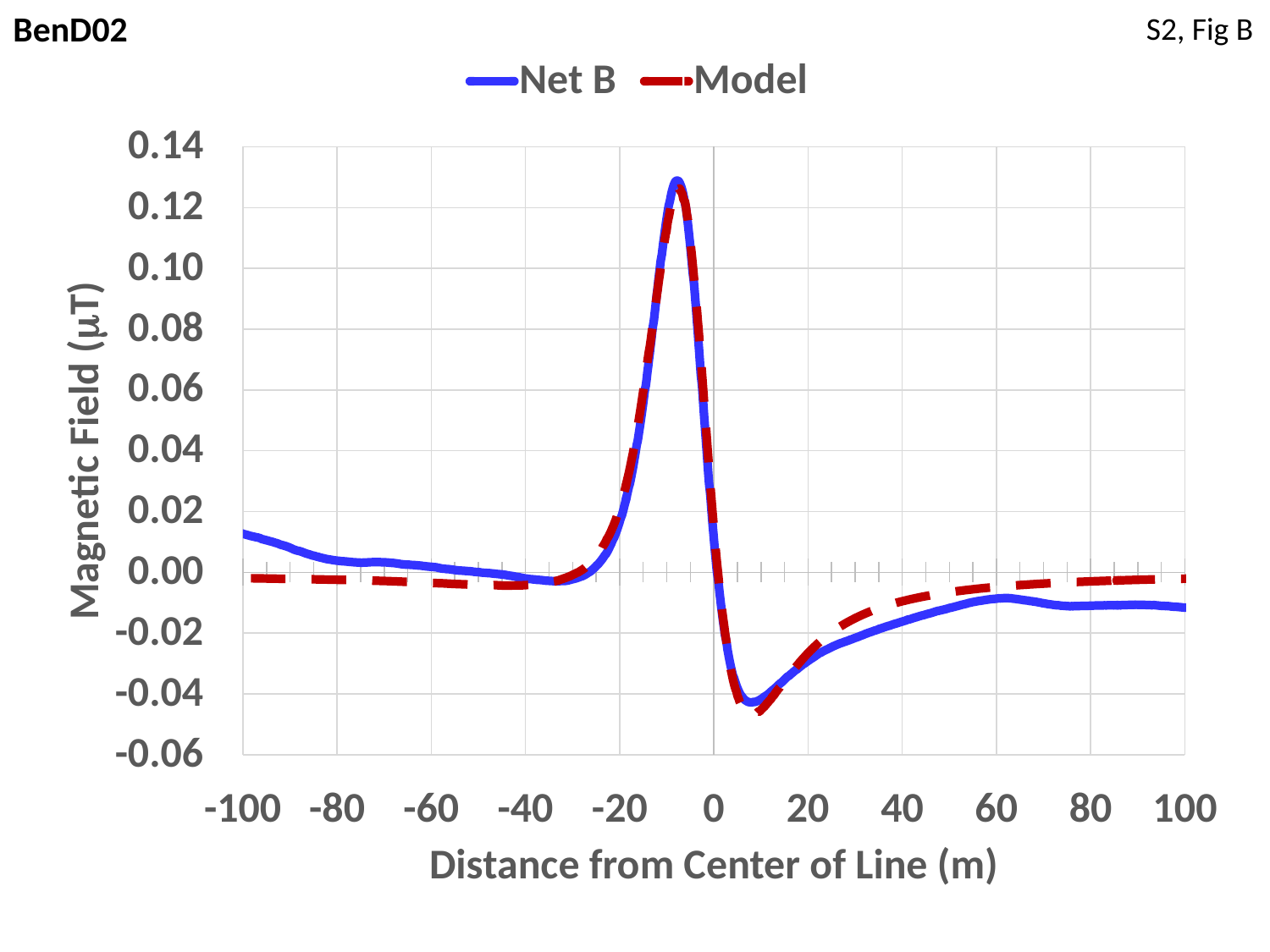

BenD02
S2, Fig B

## Slide 4
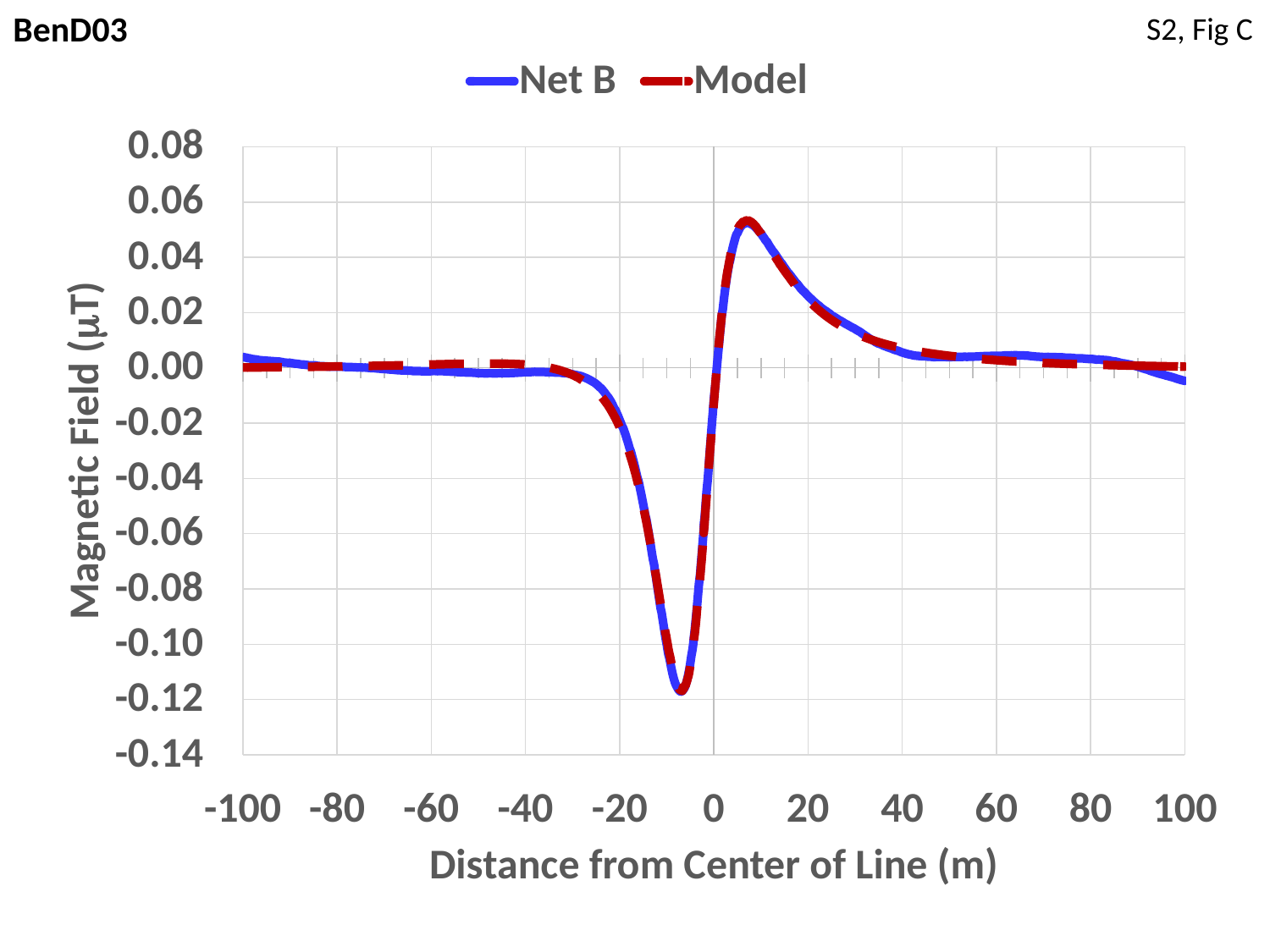

BenD03
S2, Fig C

## Slide 5
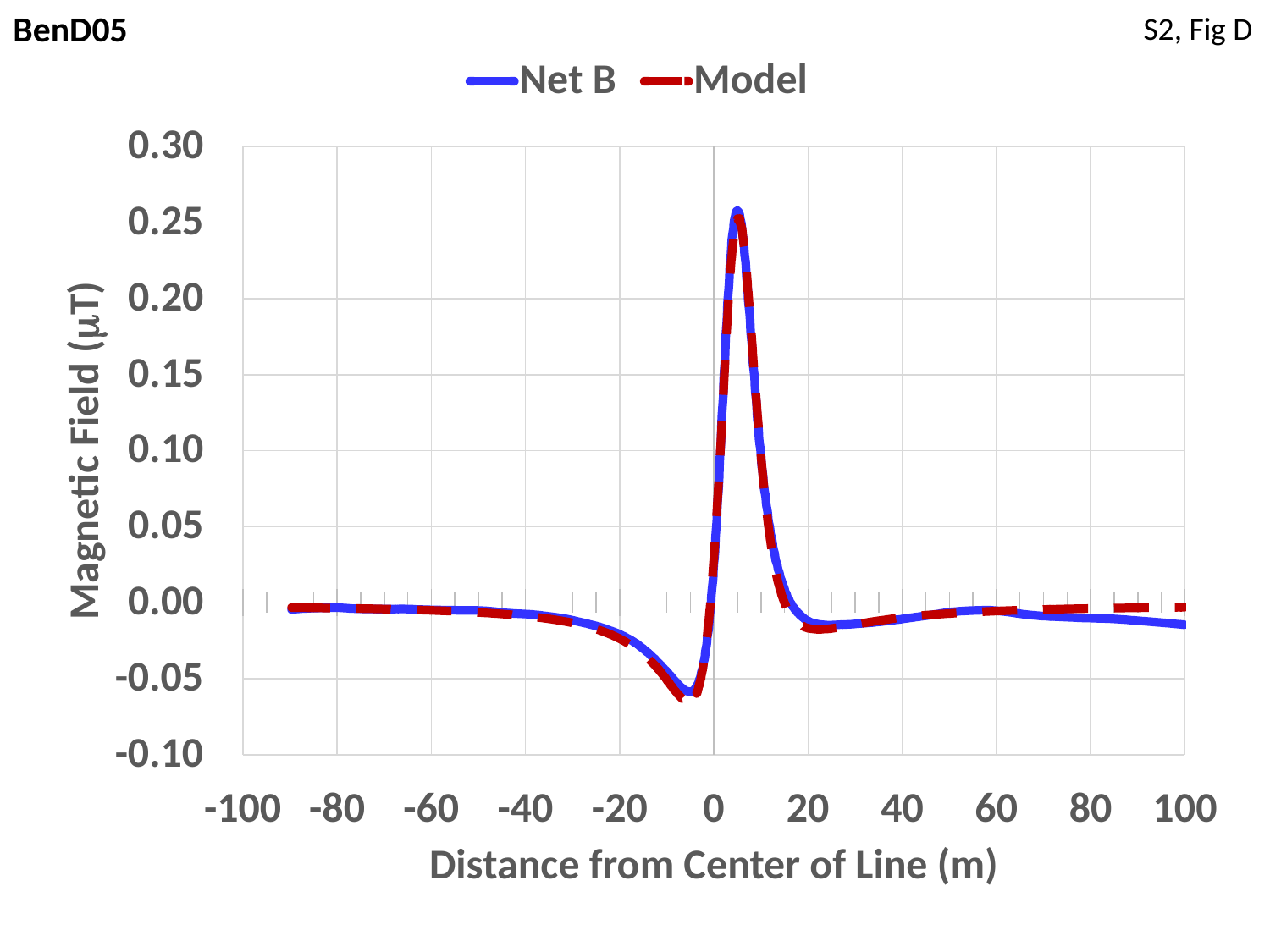

BenD05
S2, Fig D

## Slide 6
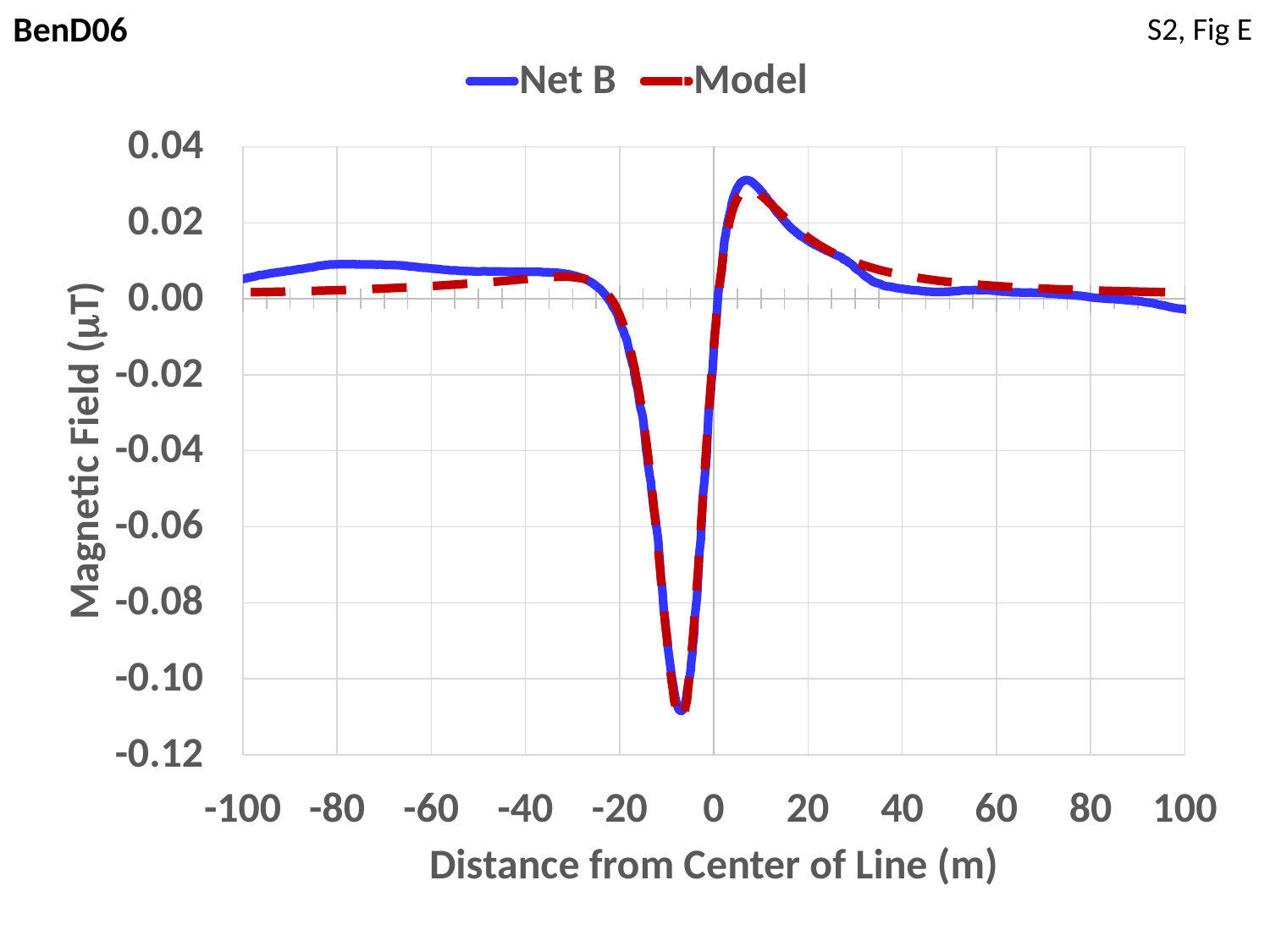

BenD06
S2, Fig E

## Slide 7
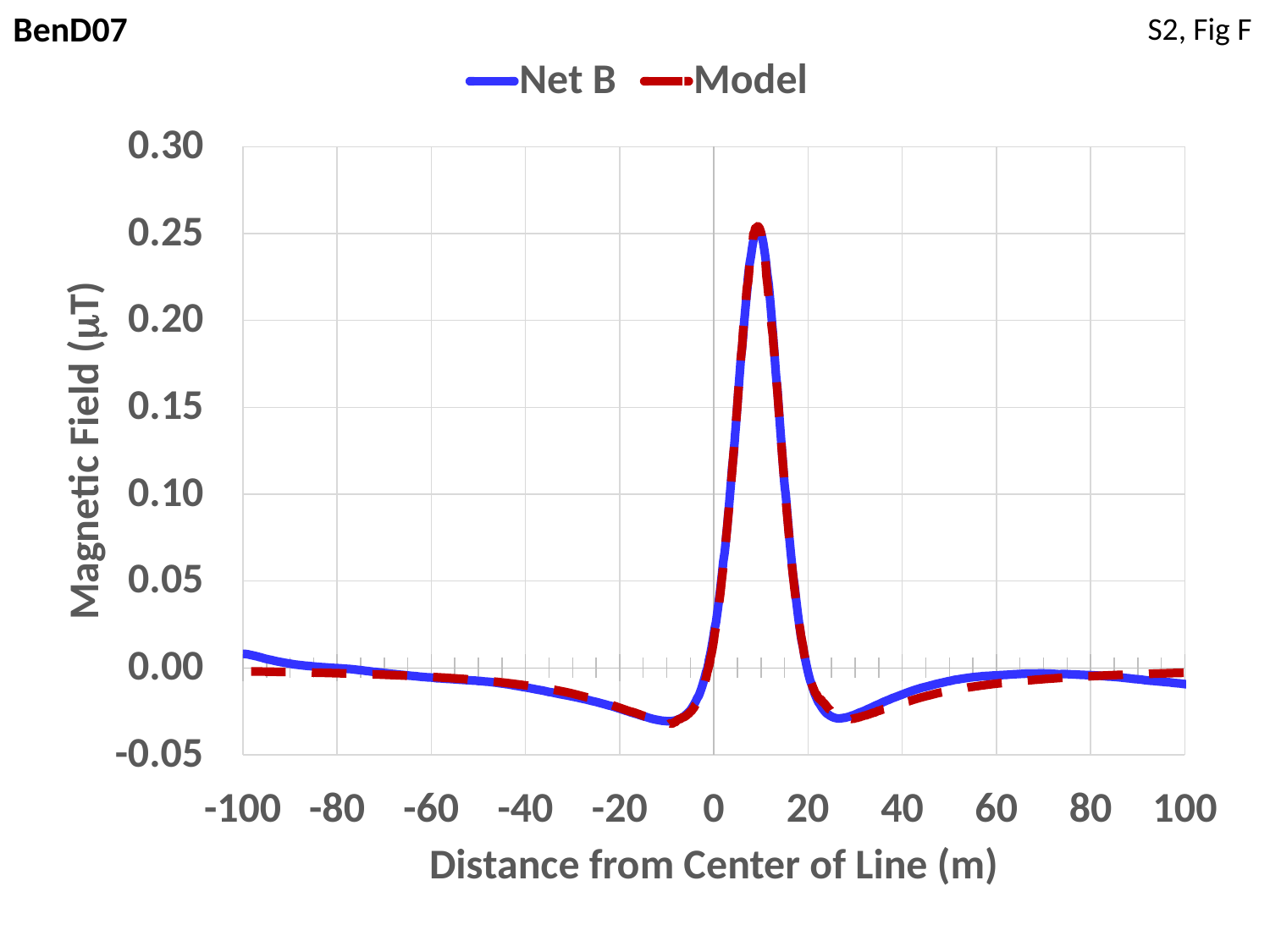

BenD07
S2, Fig F

## Slide 8
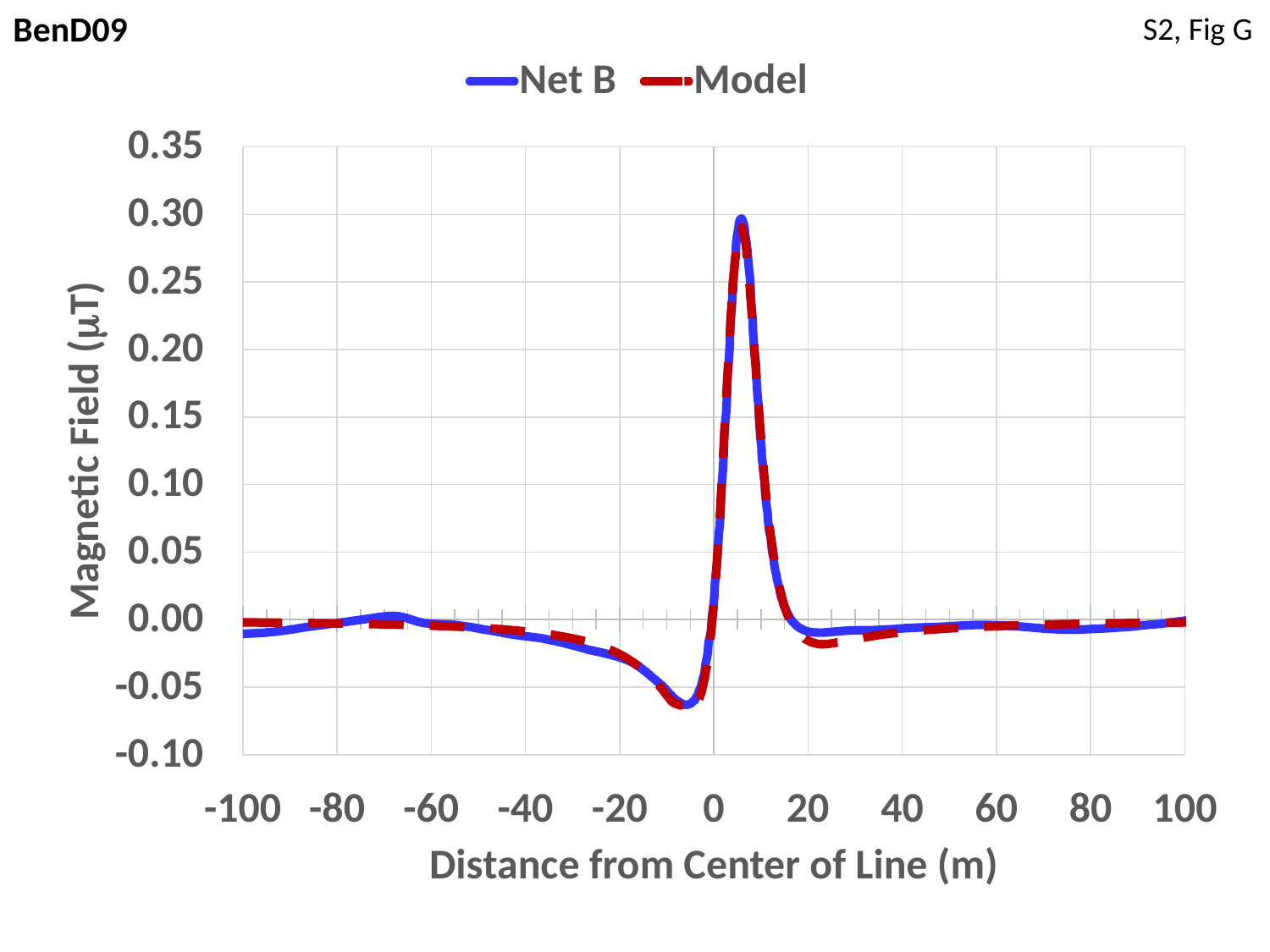

BenD09
S2, Fig G

## Slide 9
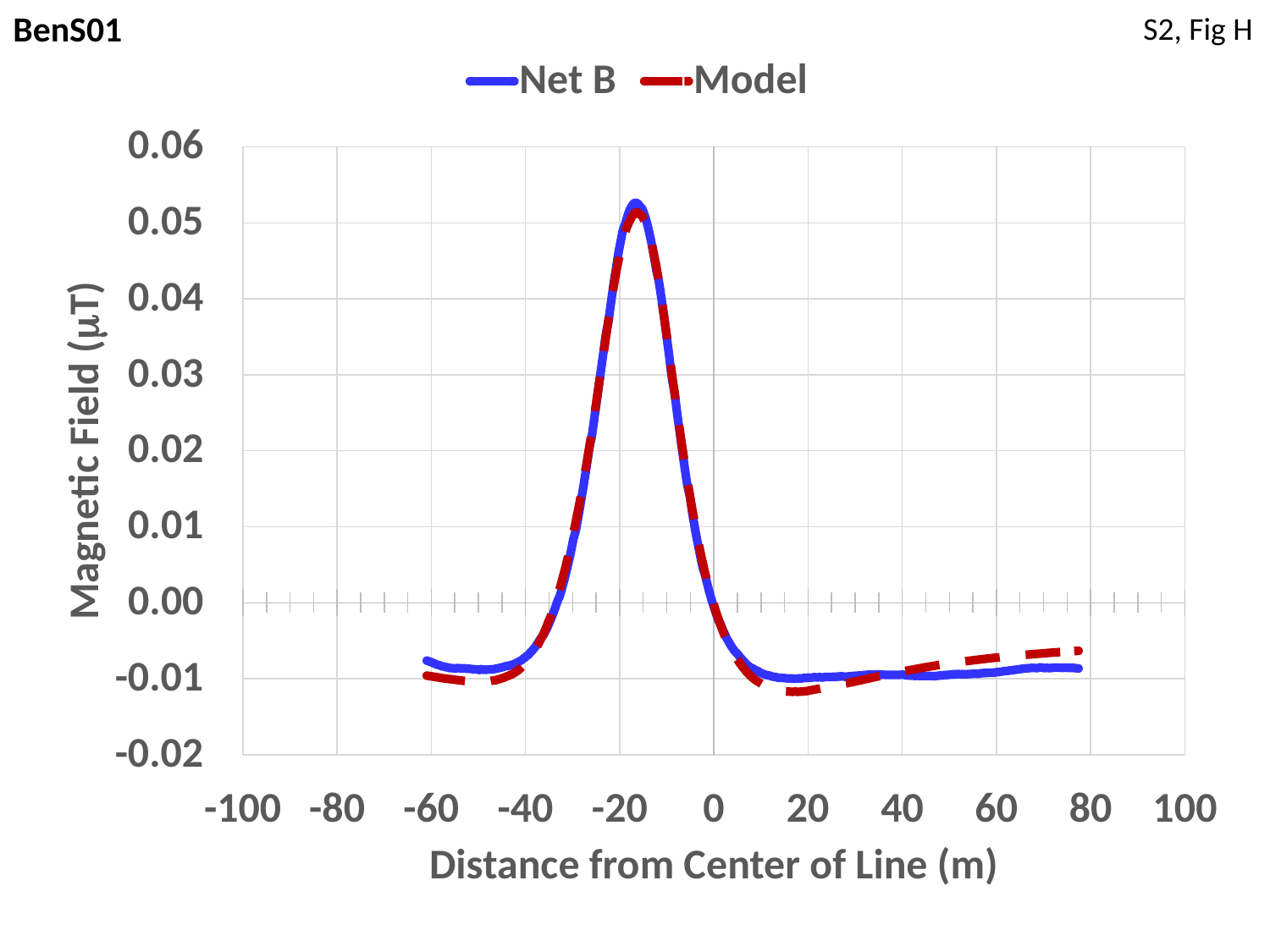

BenS01
S2, Fig H

## Slide 10
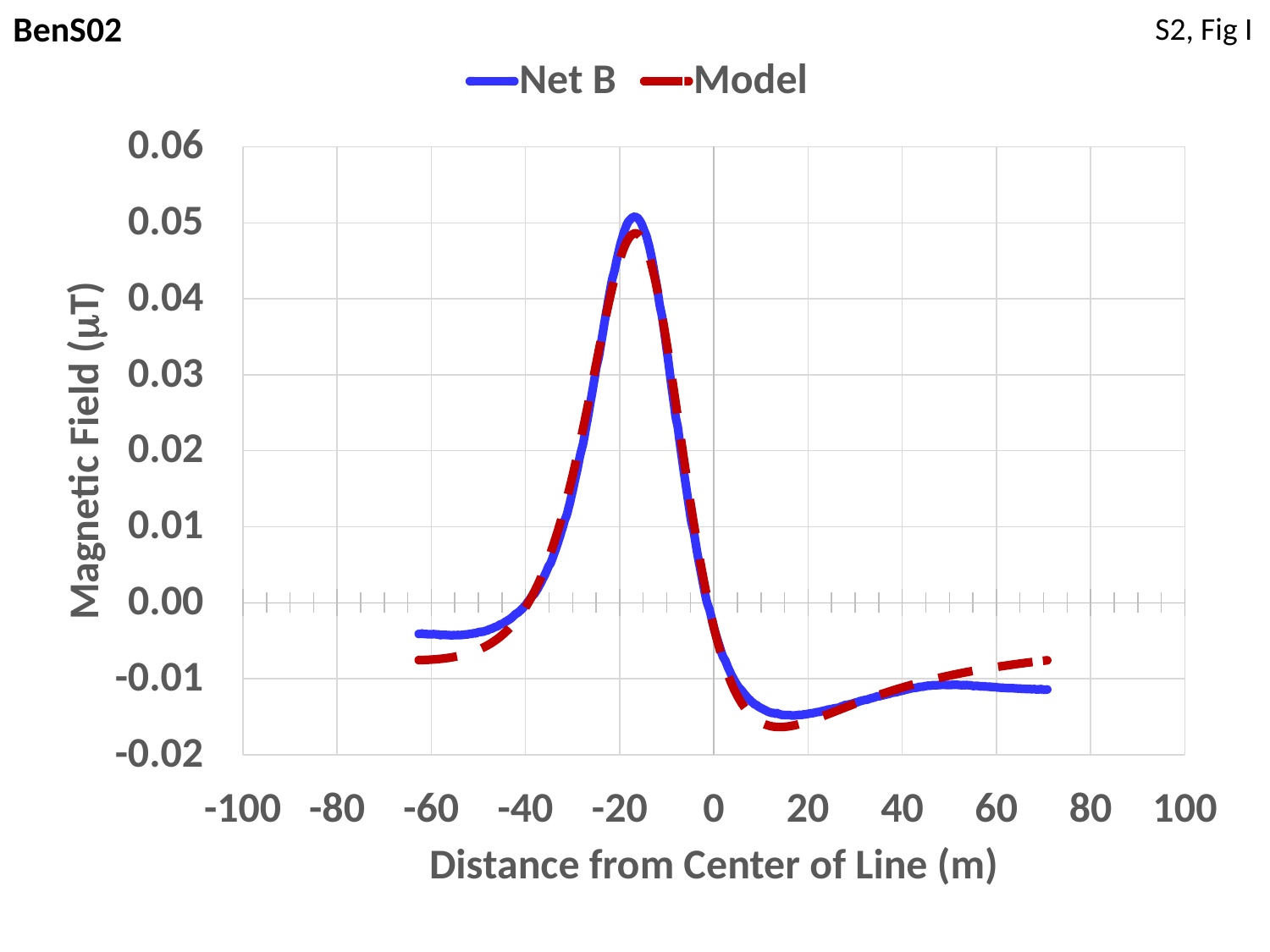

BenS02
S2, Fig I

## Slide 11
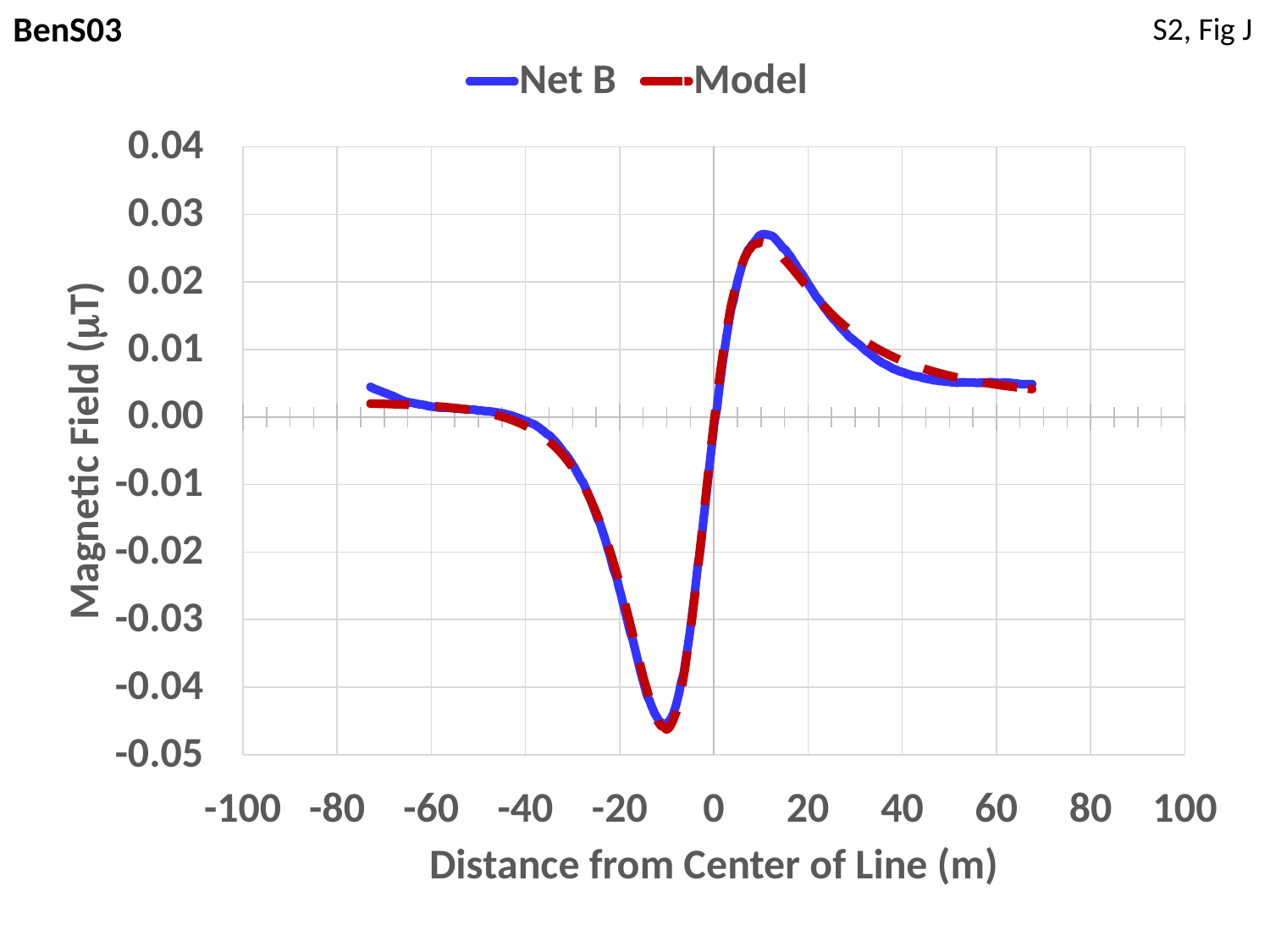

BenS03
S2, Fig J

## Slide 12
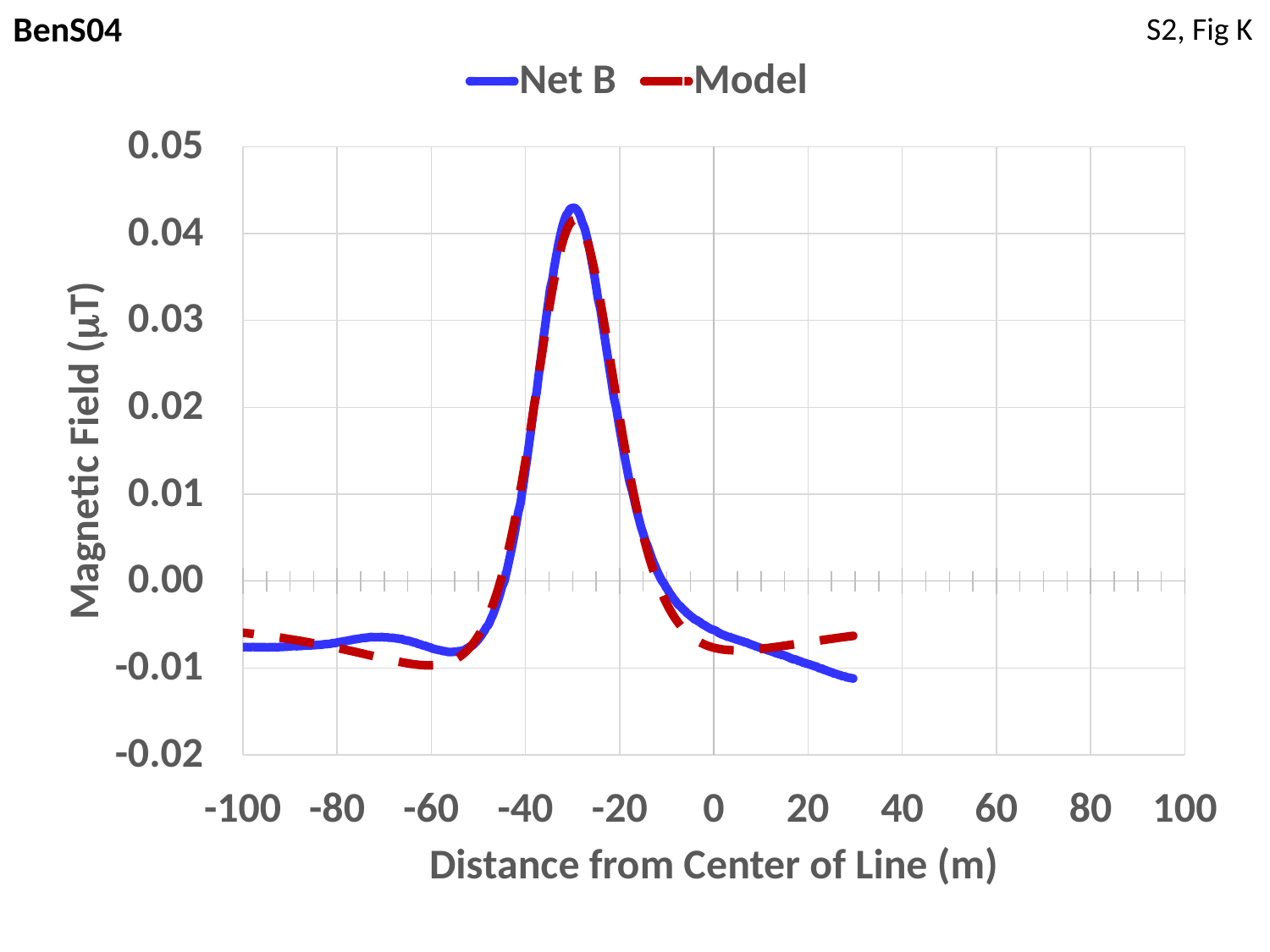

BenS04
S2, Fig K

## Slide 13
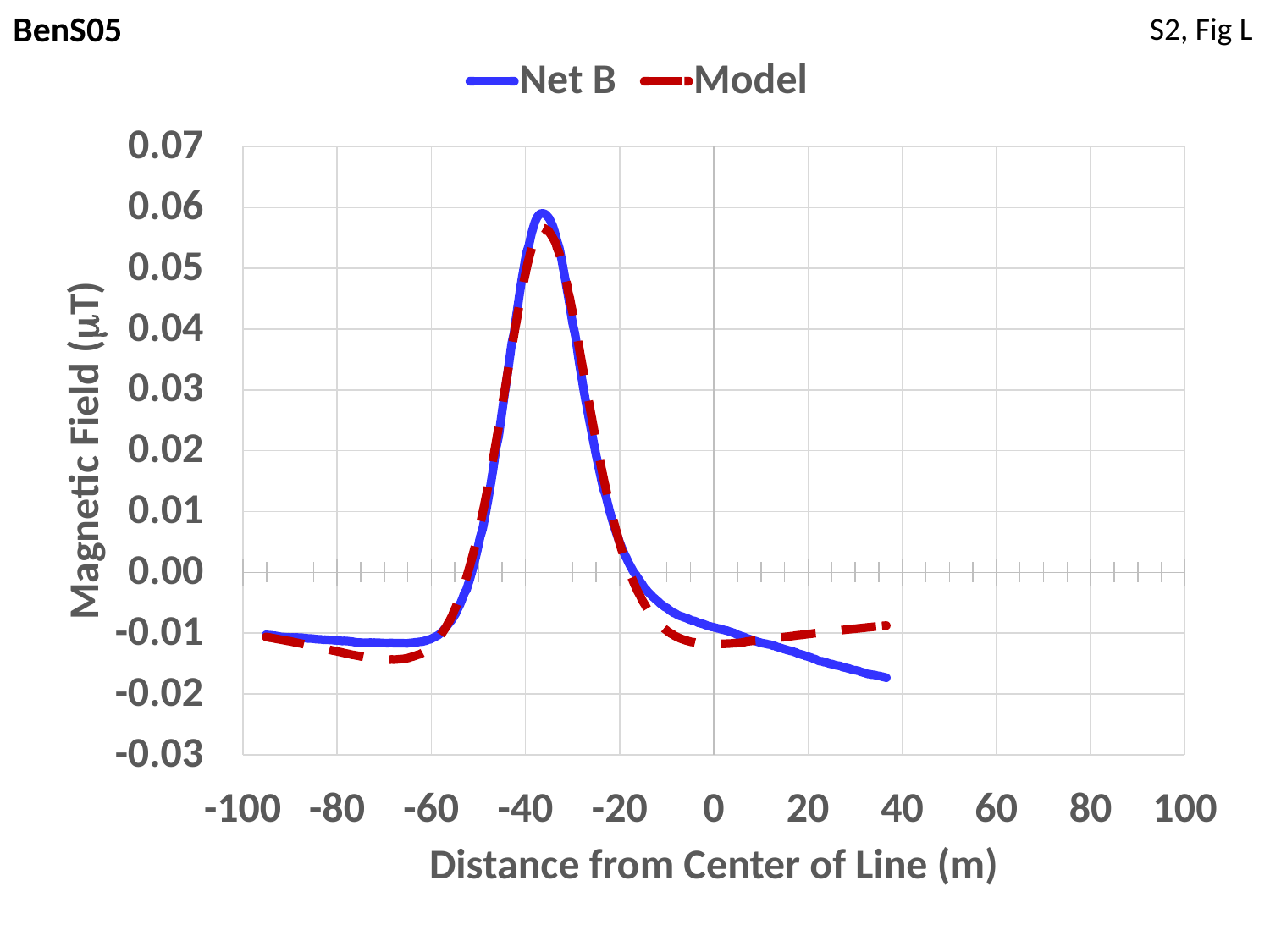

BenS05
S2, Fig L

## Slide 14
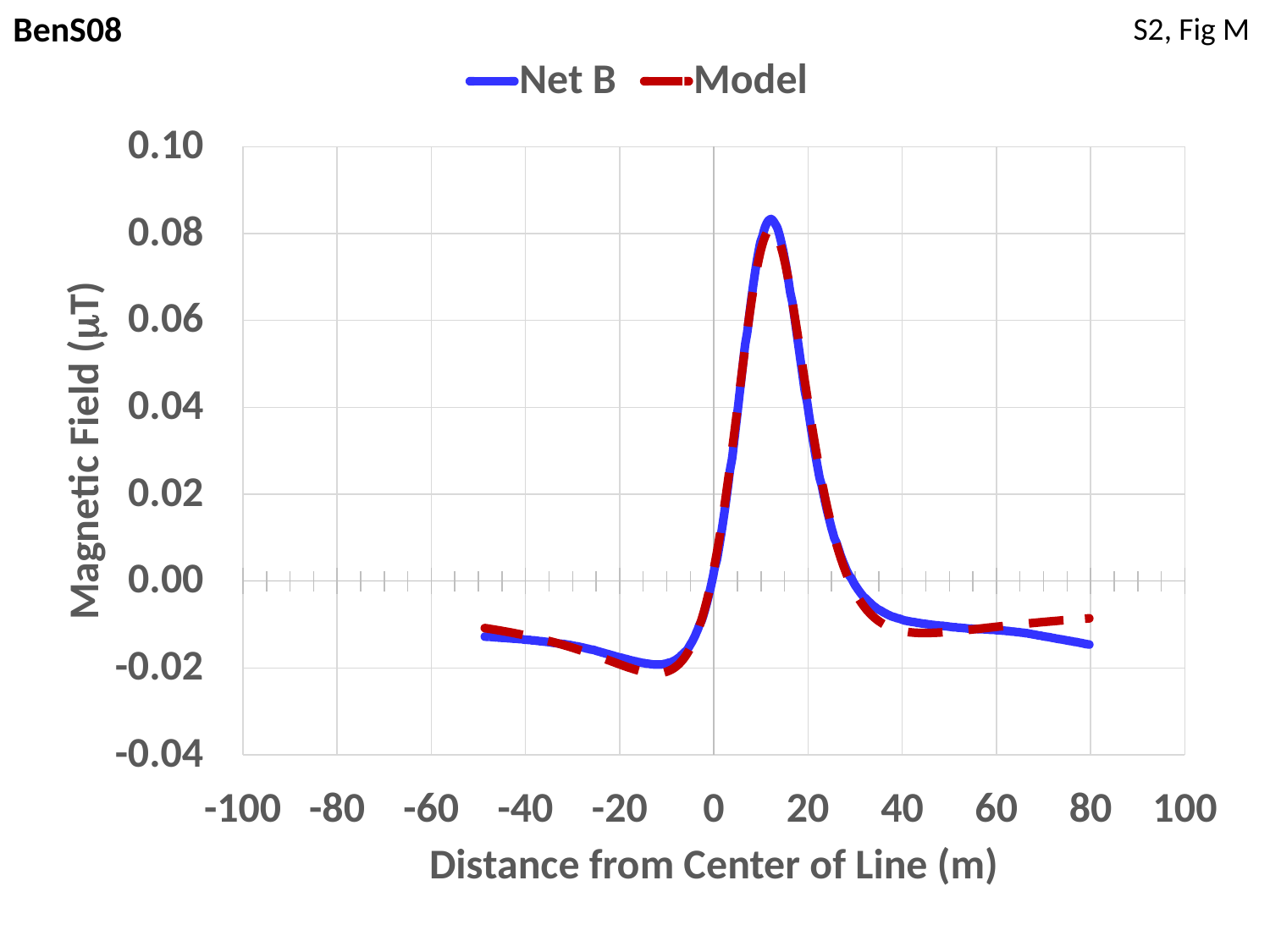

BenS08
S2, Fig M

## Slide 15
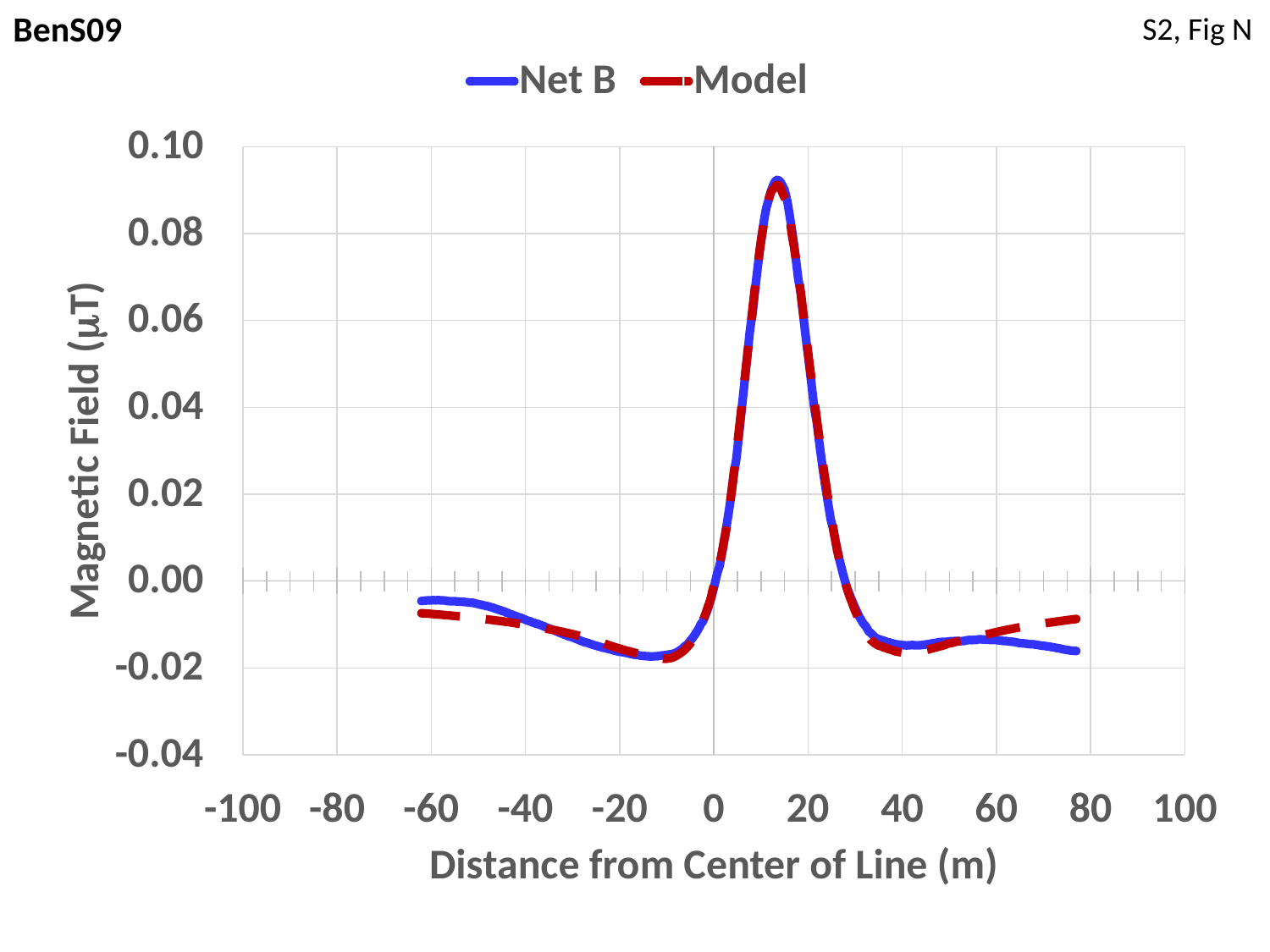

BenS09
S2, Fig N

## Slide 16
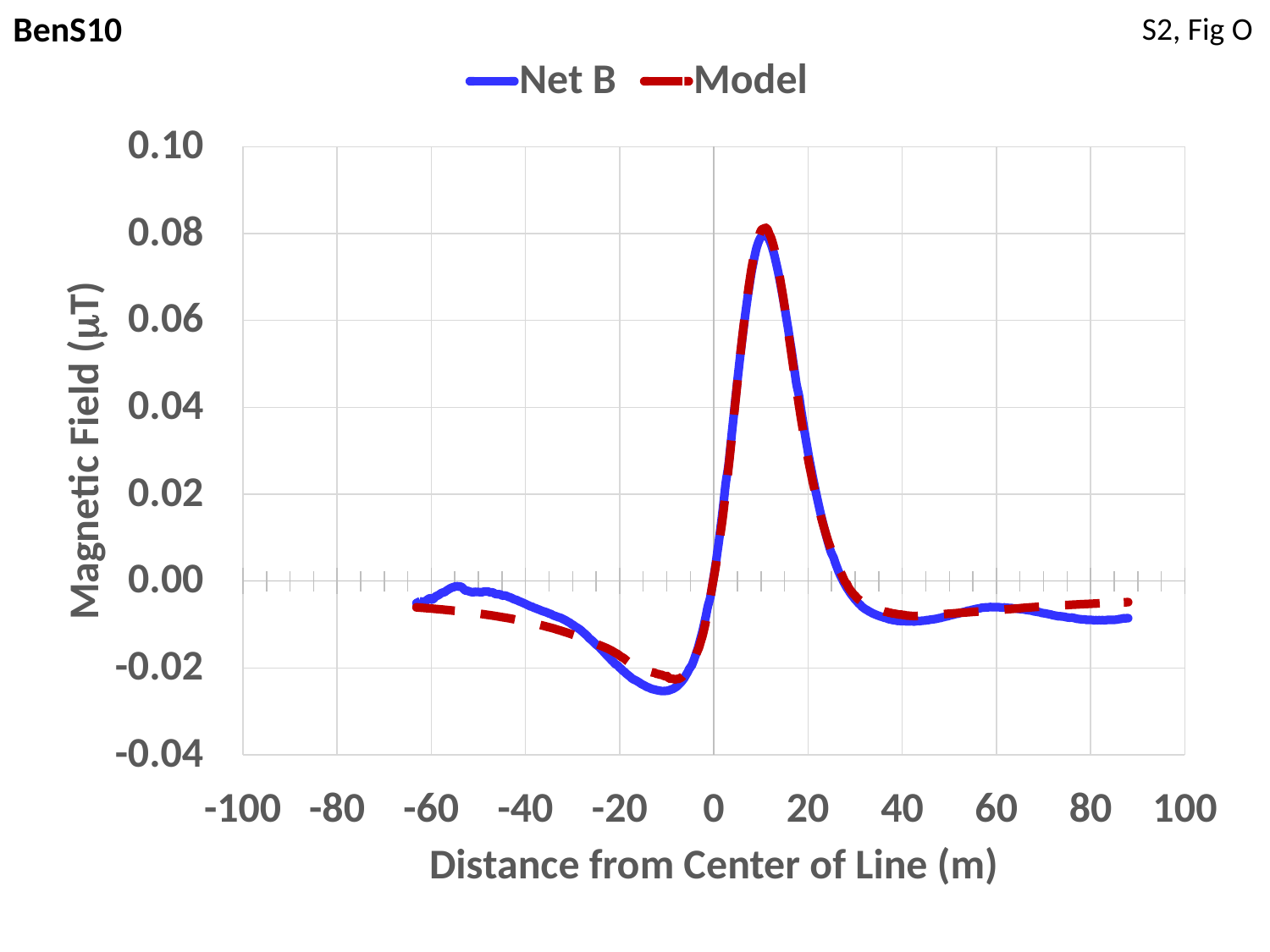

BenS10
S2, Fig O

## Slide 17
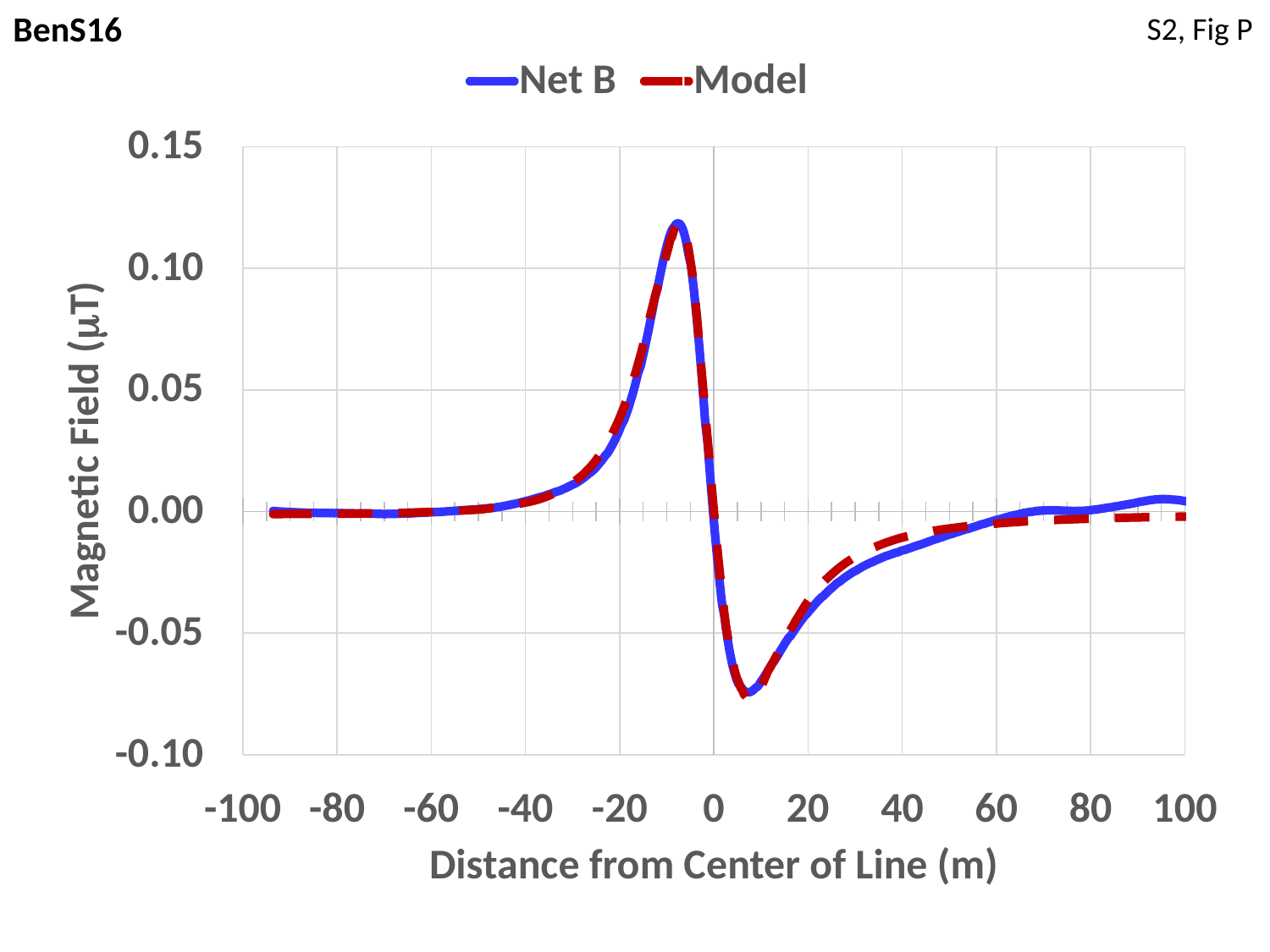

BenS16
S2, Fig P

## Slide 18
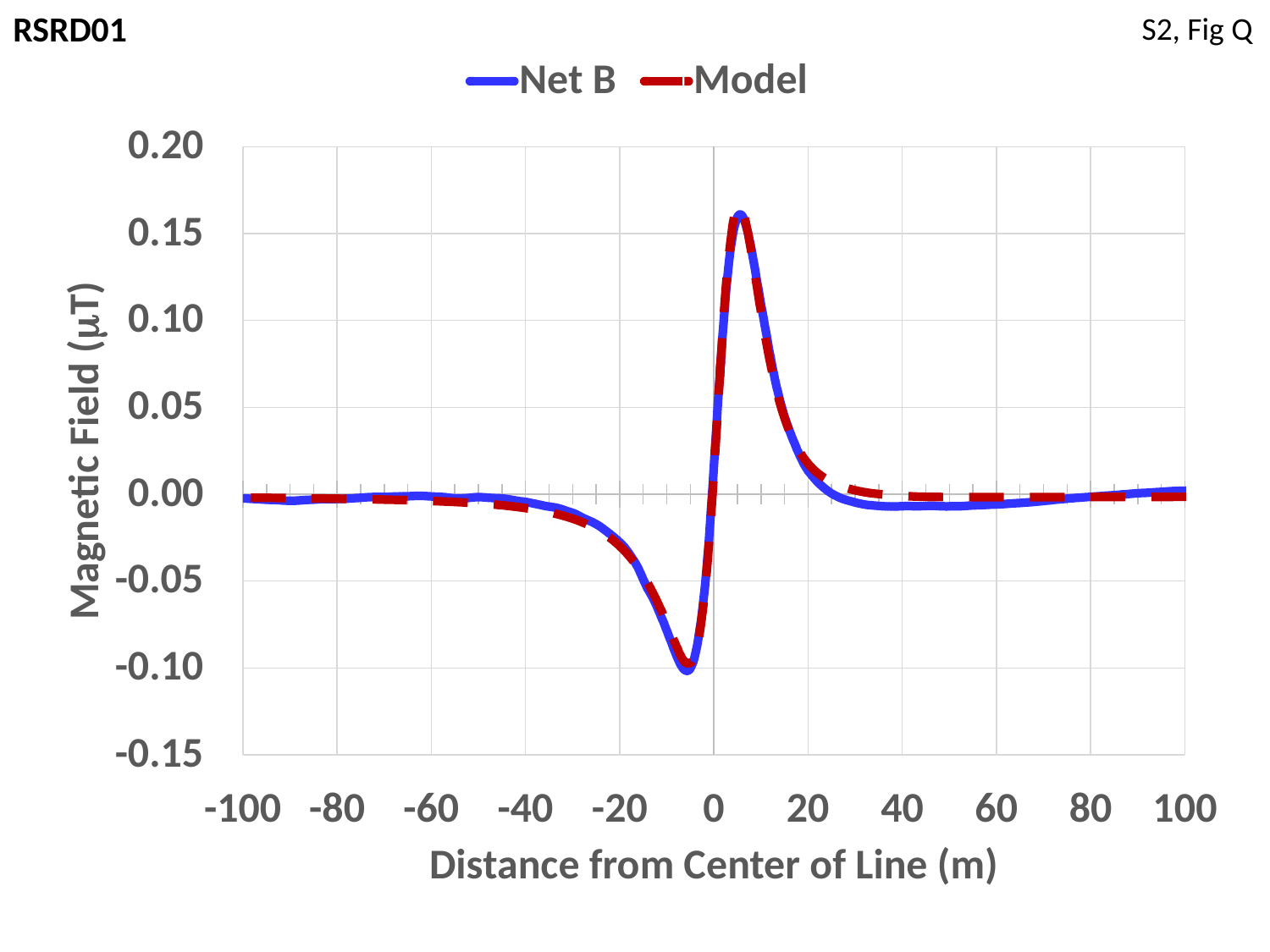

RSRD01
S2, Fig Q

## Slide 19
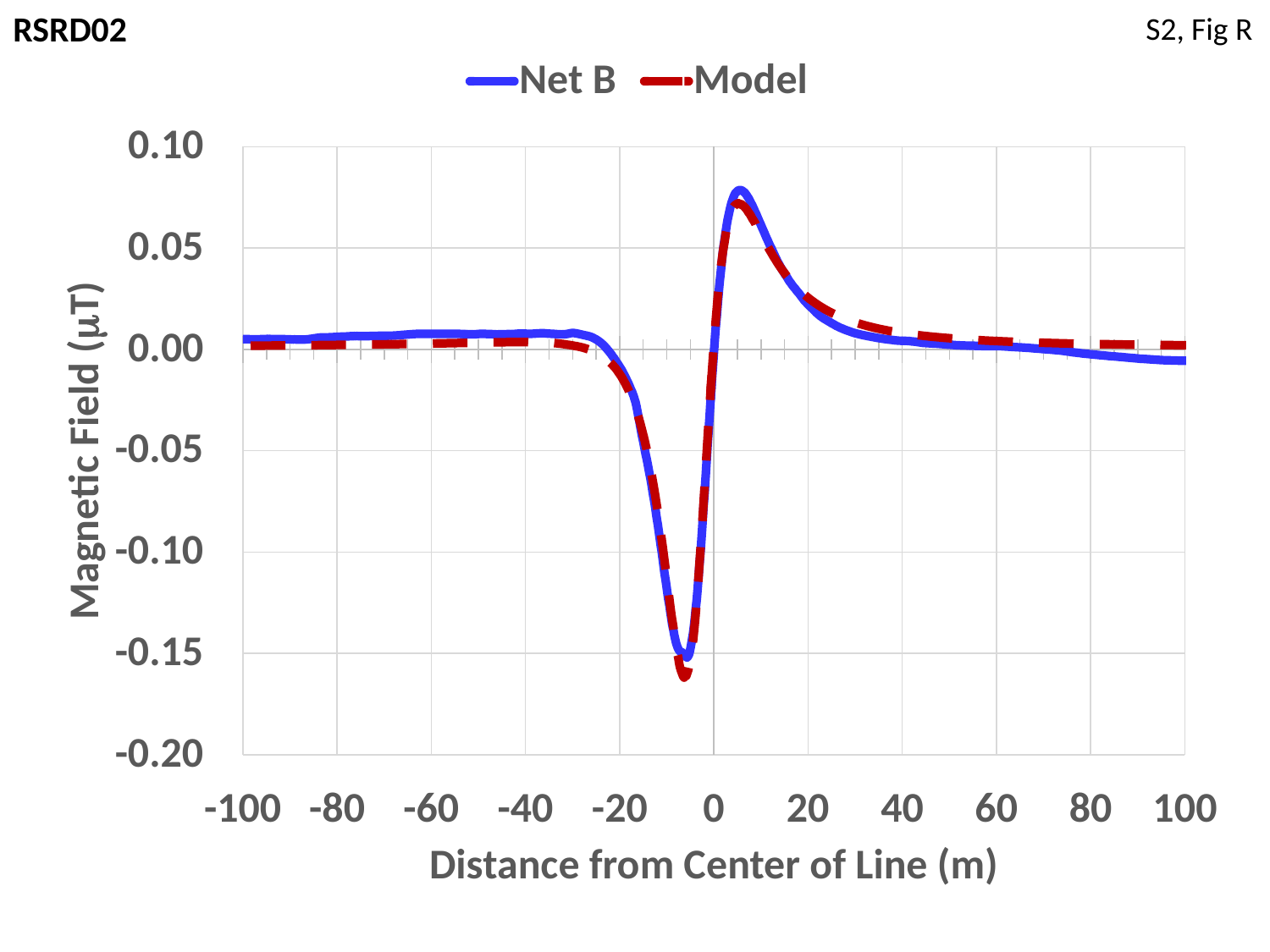

RSRD02
S2, Fig R

## Slide 20
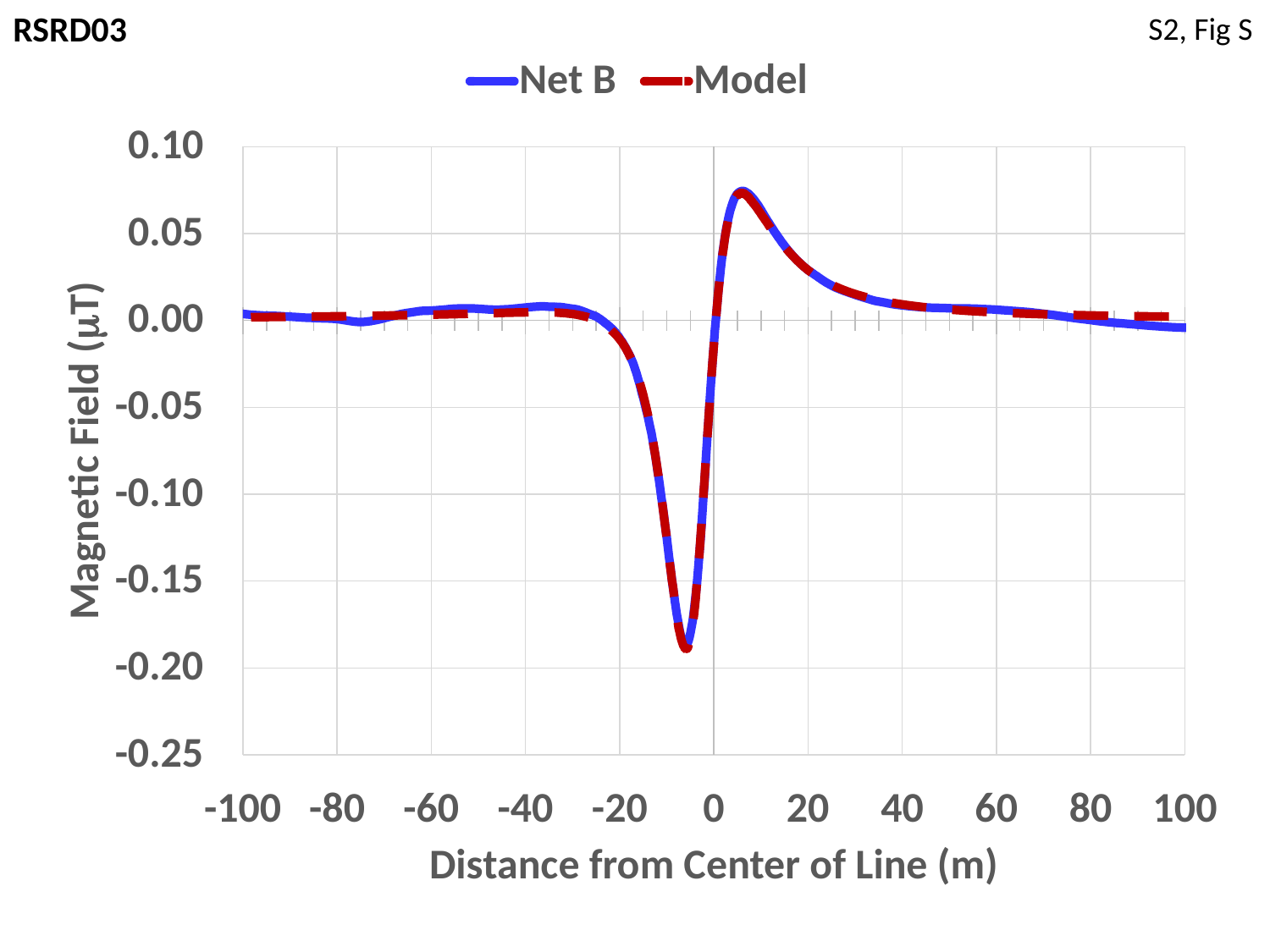

RSRD03
S2, Fig S

## Slide 21
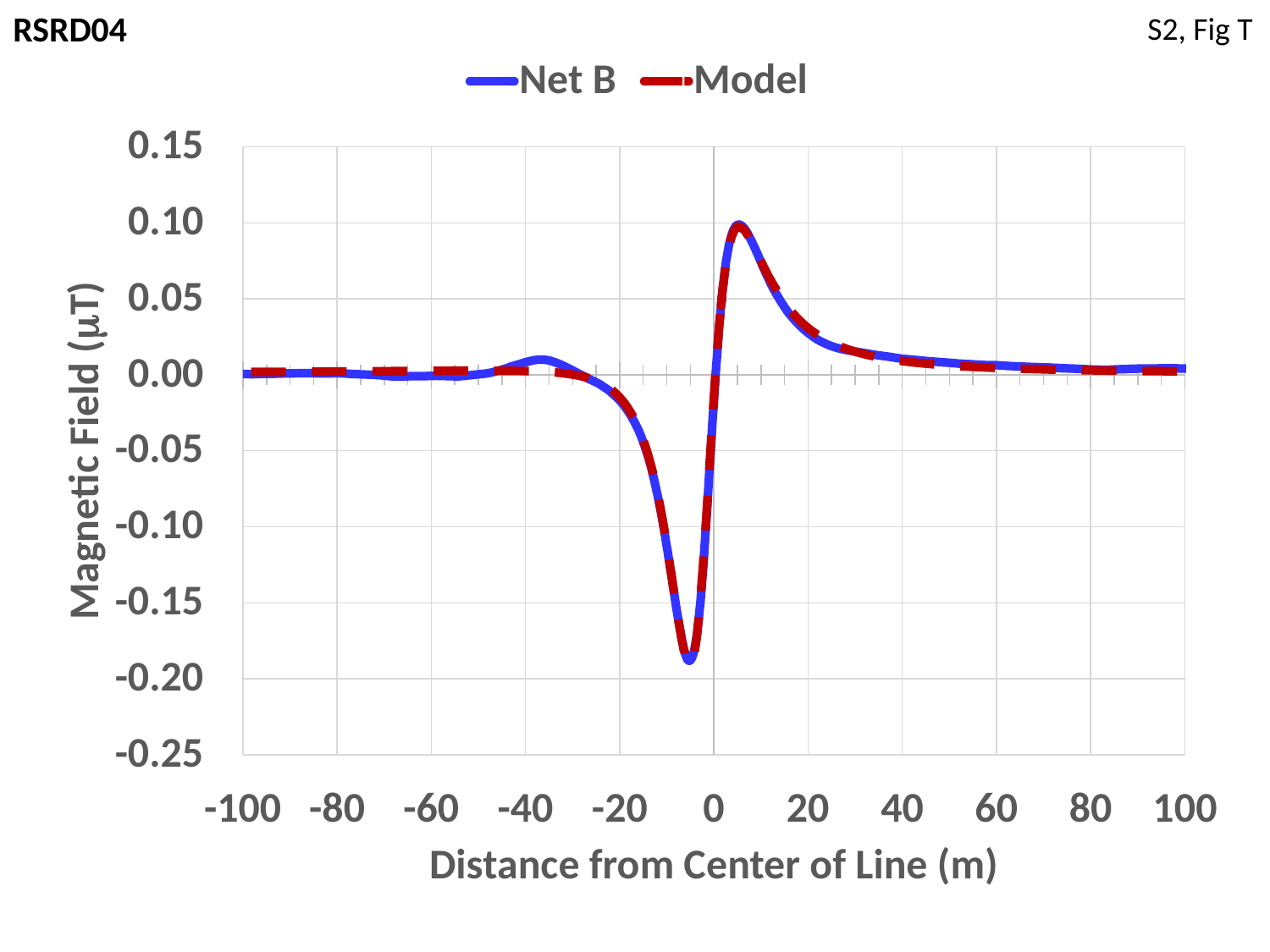

RSRD04
S2, Fig T

## Slide 22
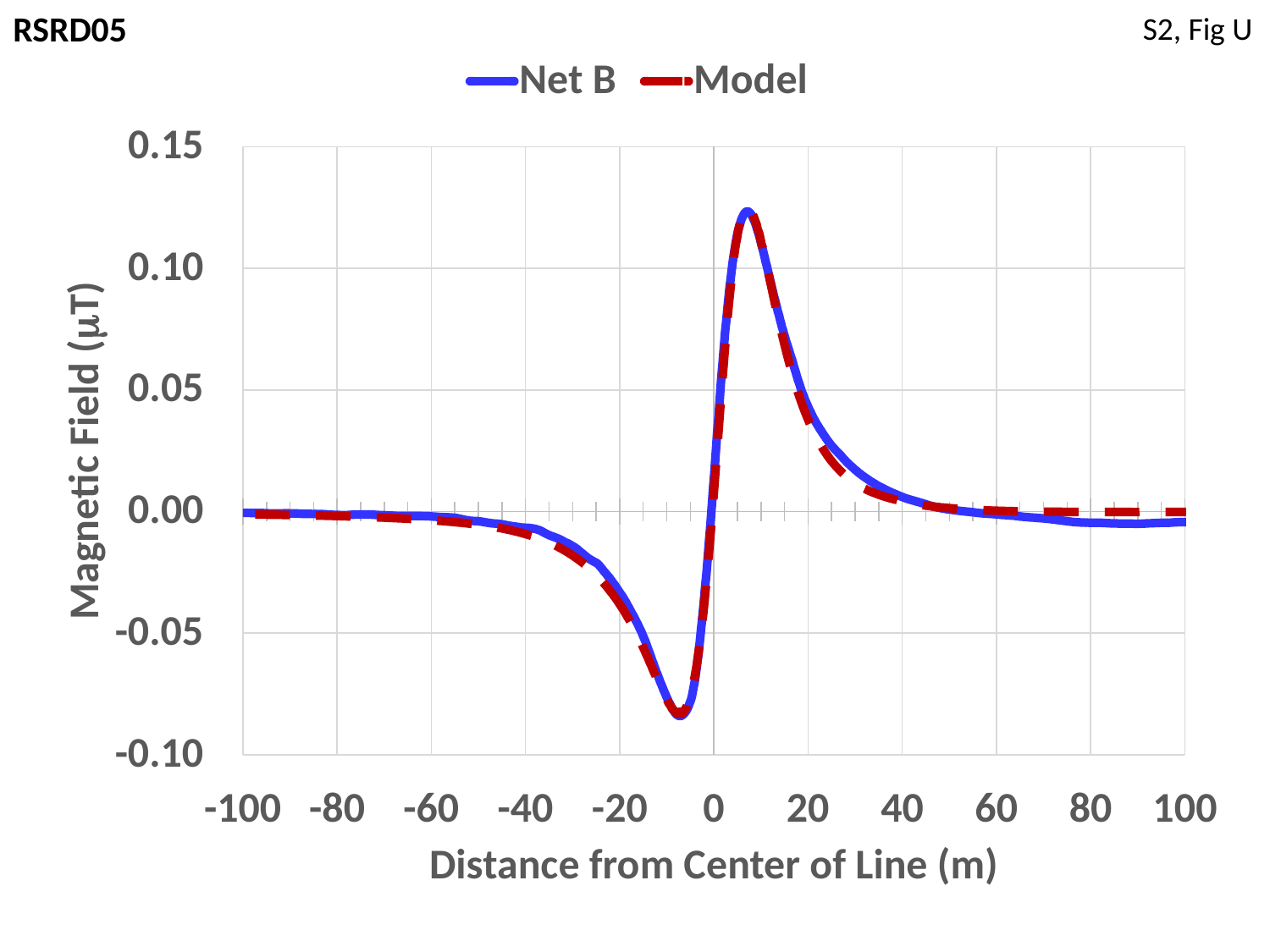

RSRD05
S2, Fig U

## Slide 23
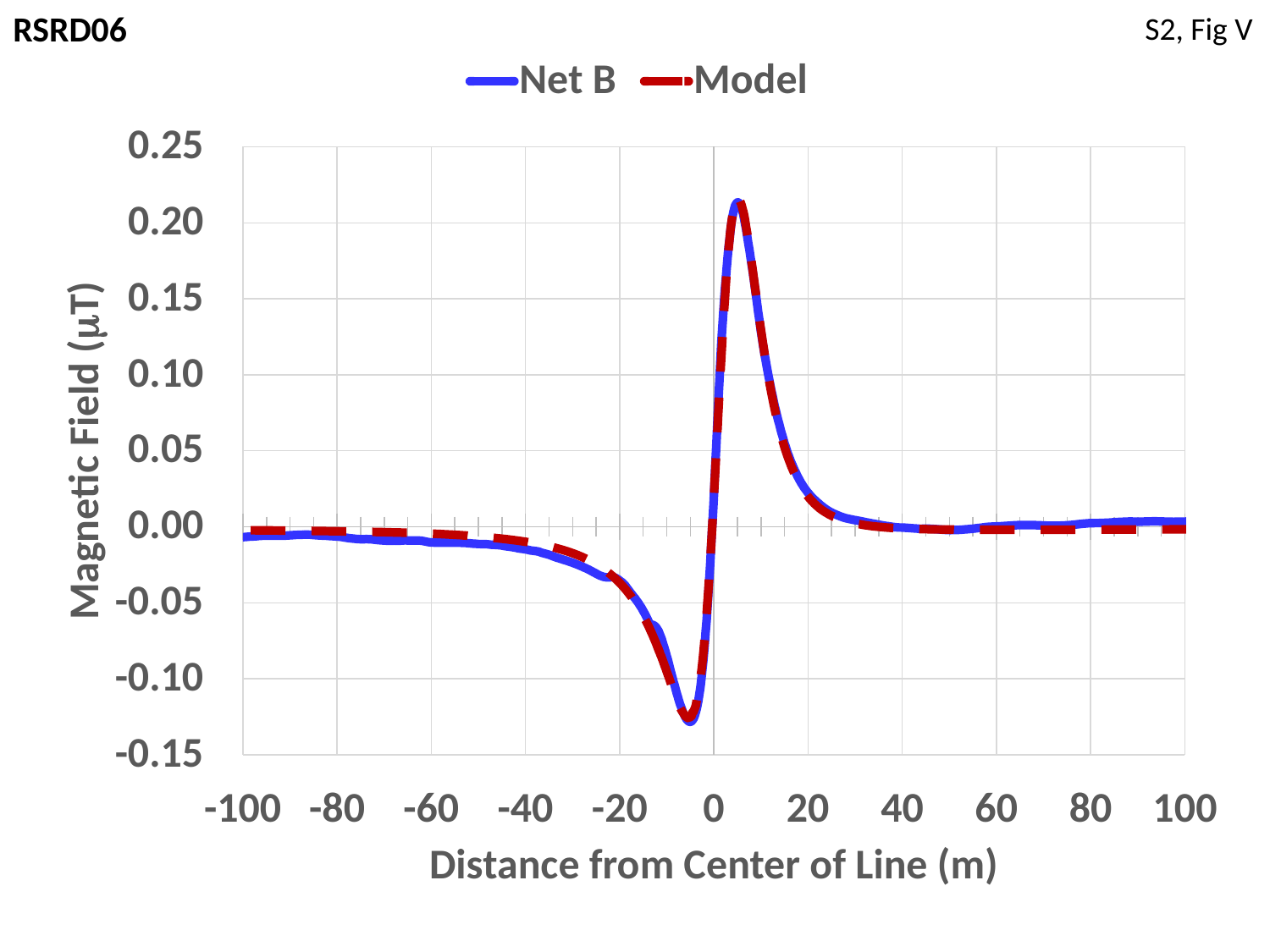

RSRD06
S2, Fig V

## Slide 24
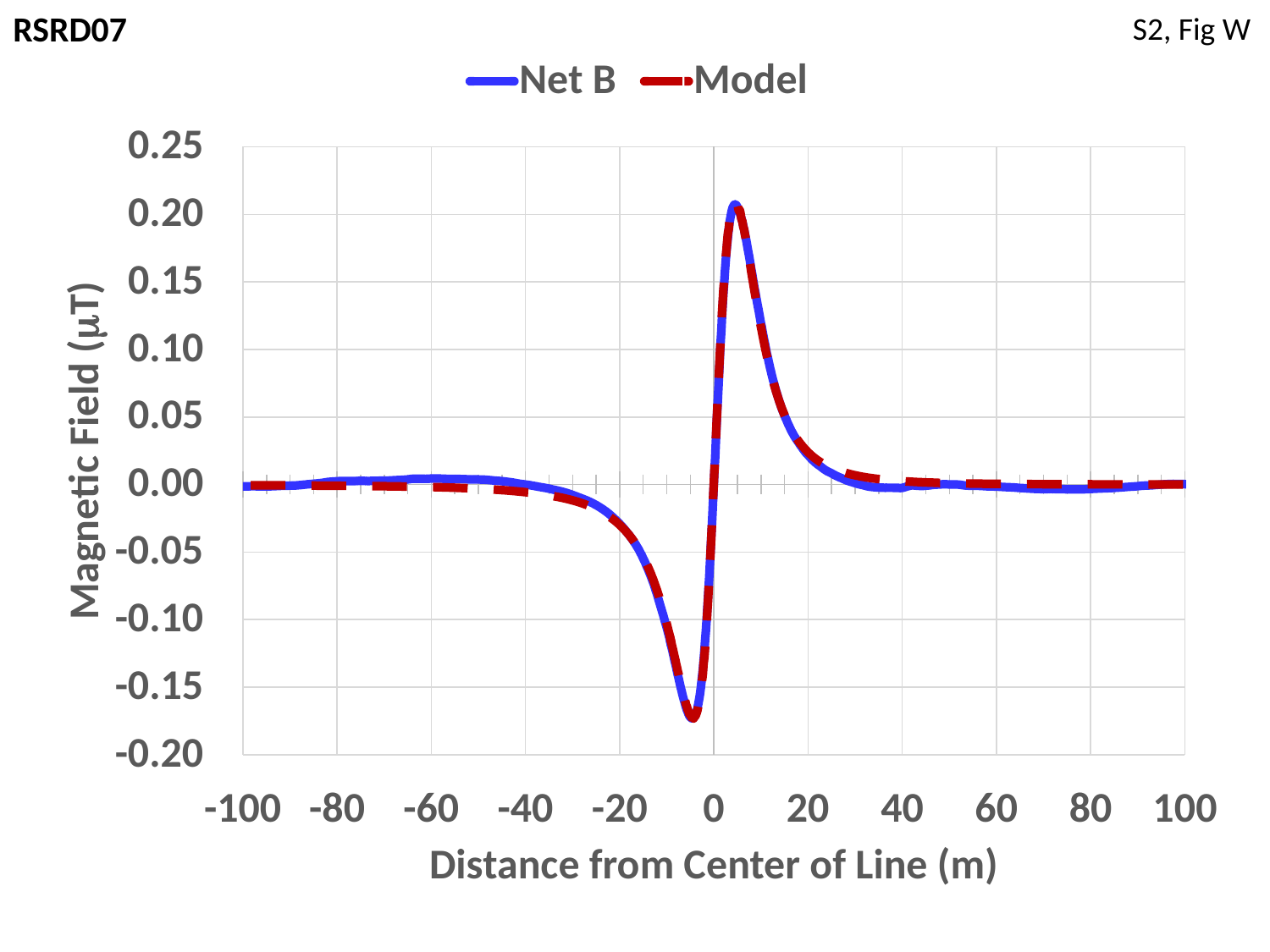

RSRD07
S2, Fig W

## Slide 25
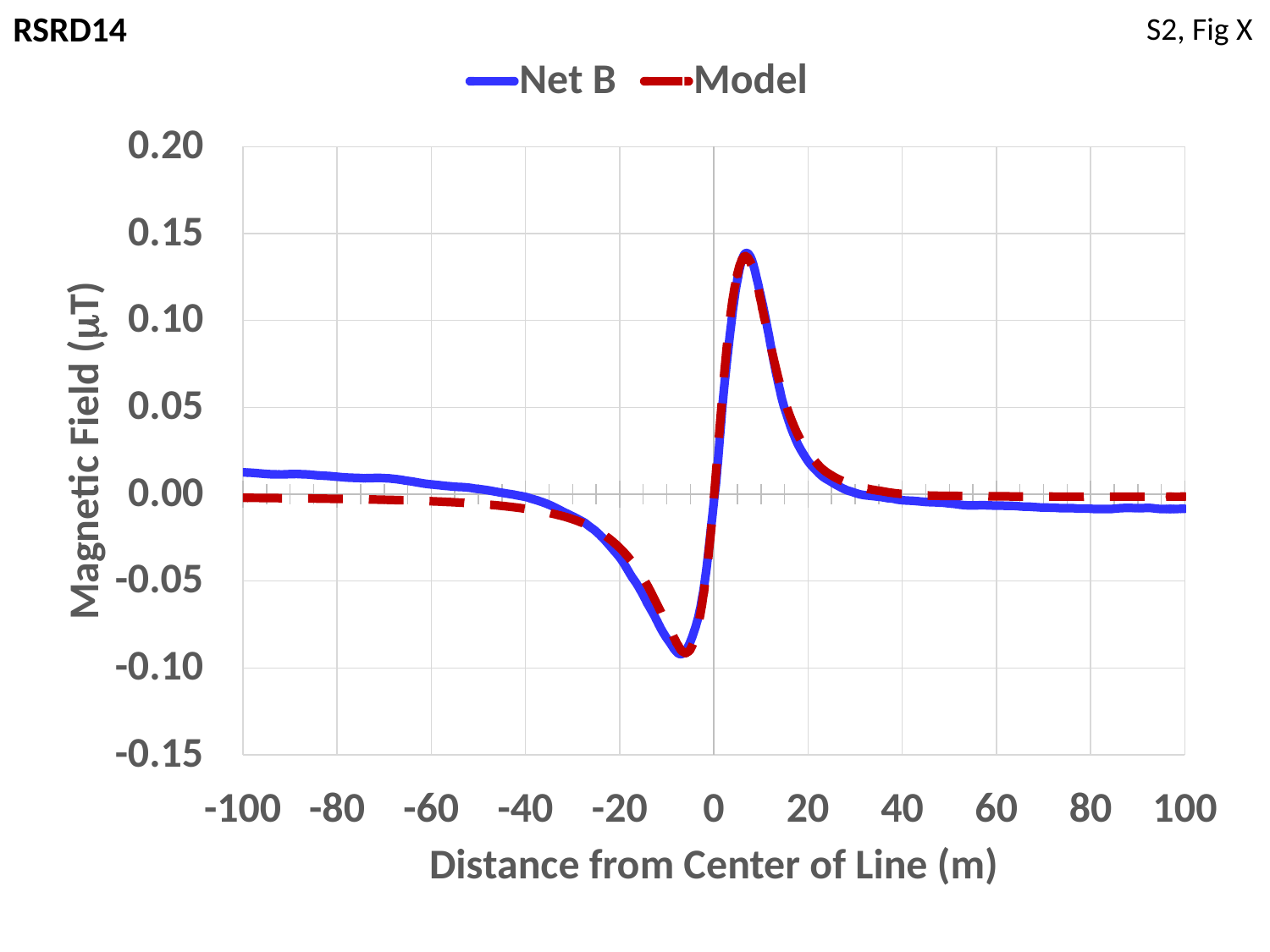

RSRD14
S2, Fig X

## Slide 26
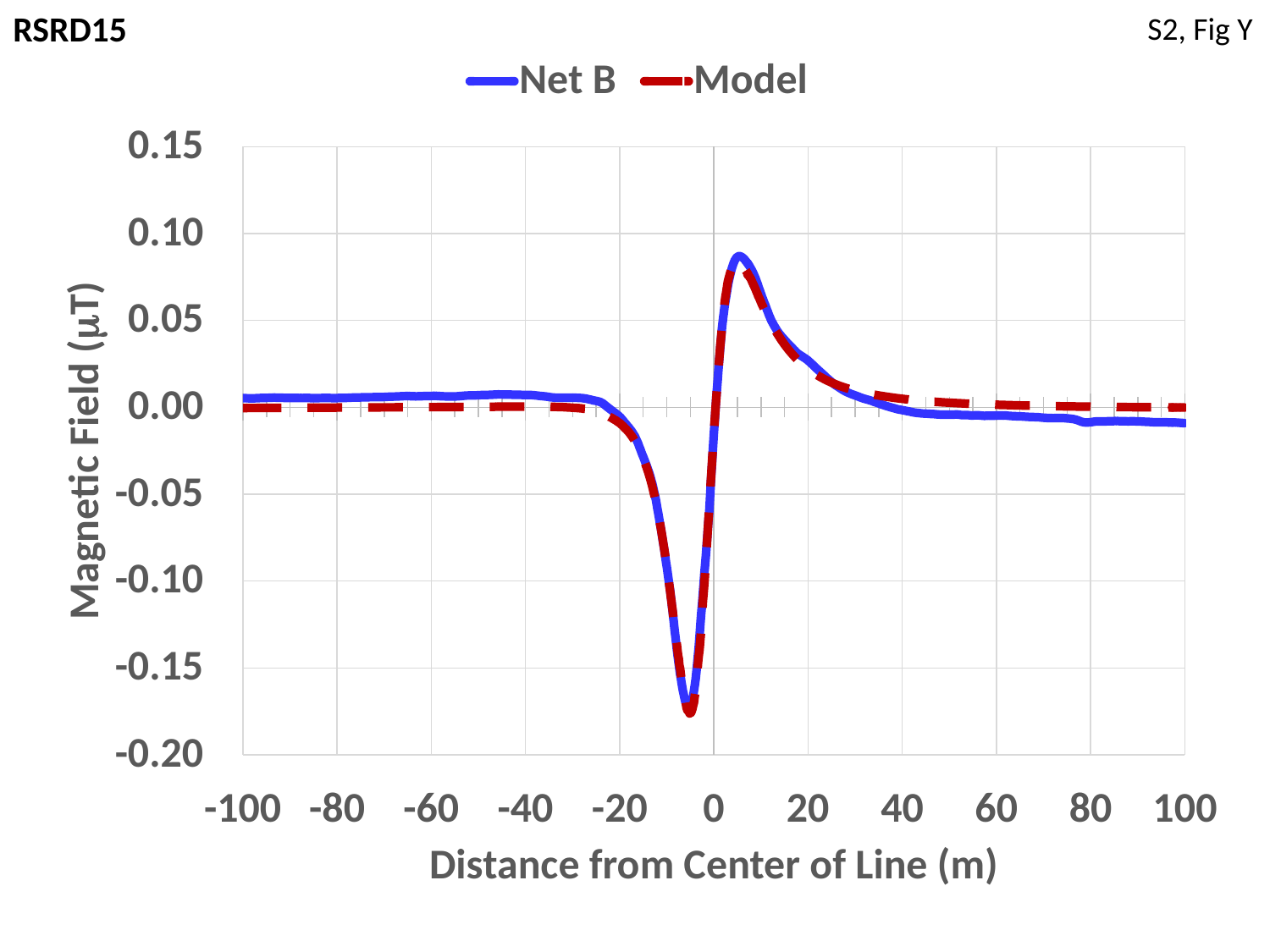

RSRD15
S2, Fig Y

## Slide 27
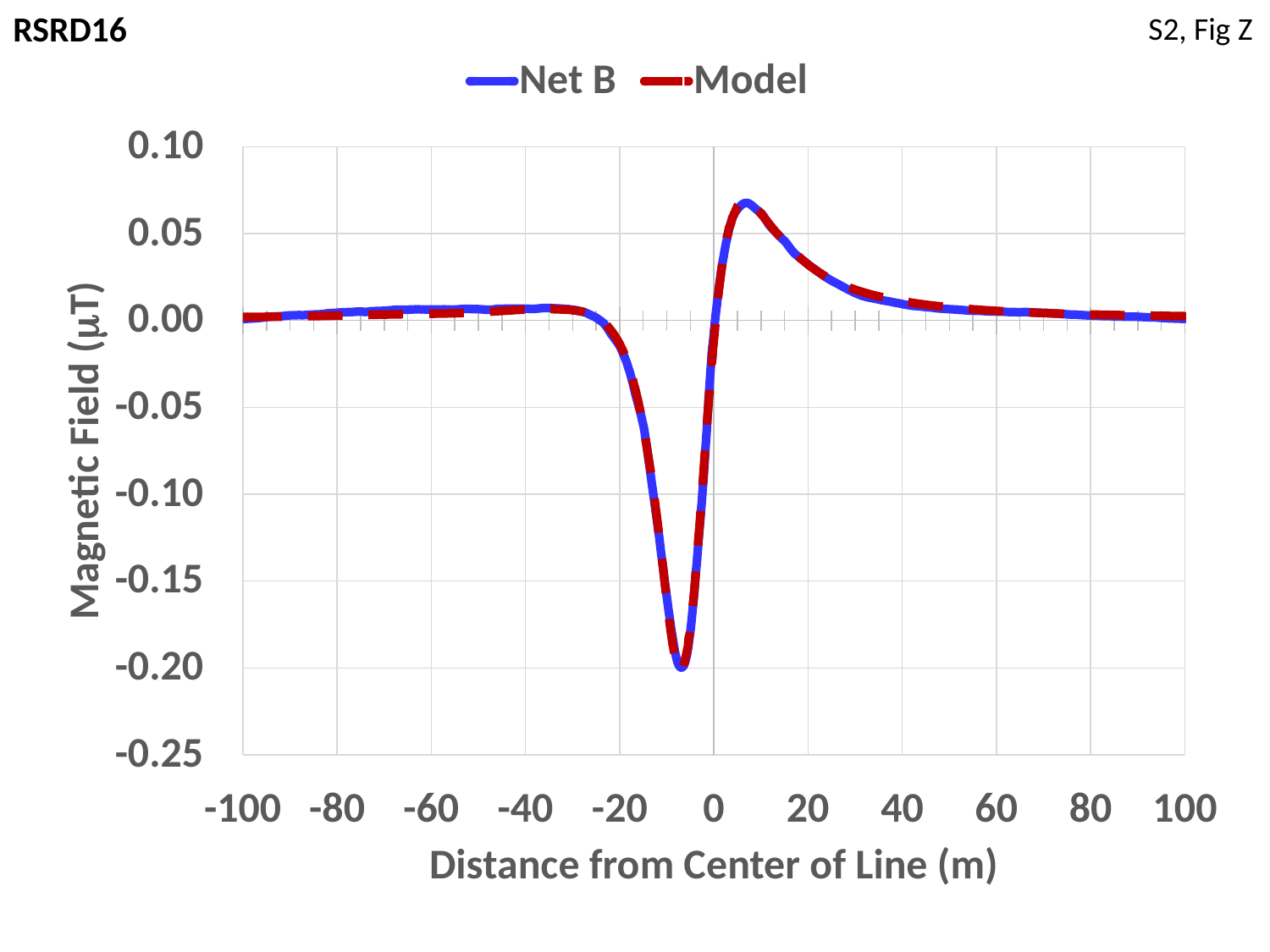

RSRD16
S2, Fig Z

## Slide 28
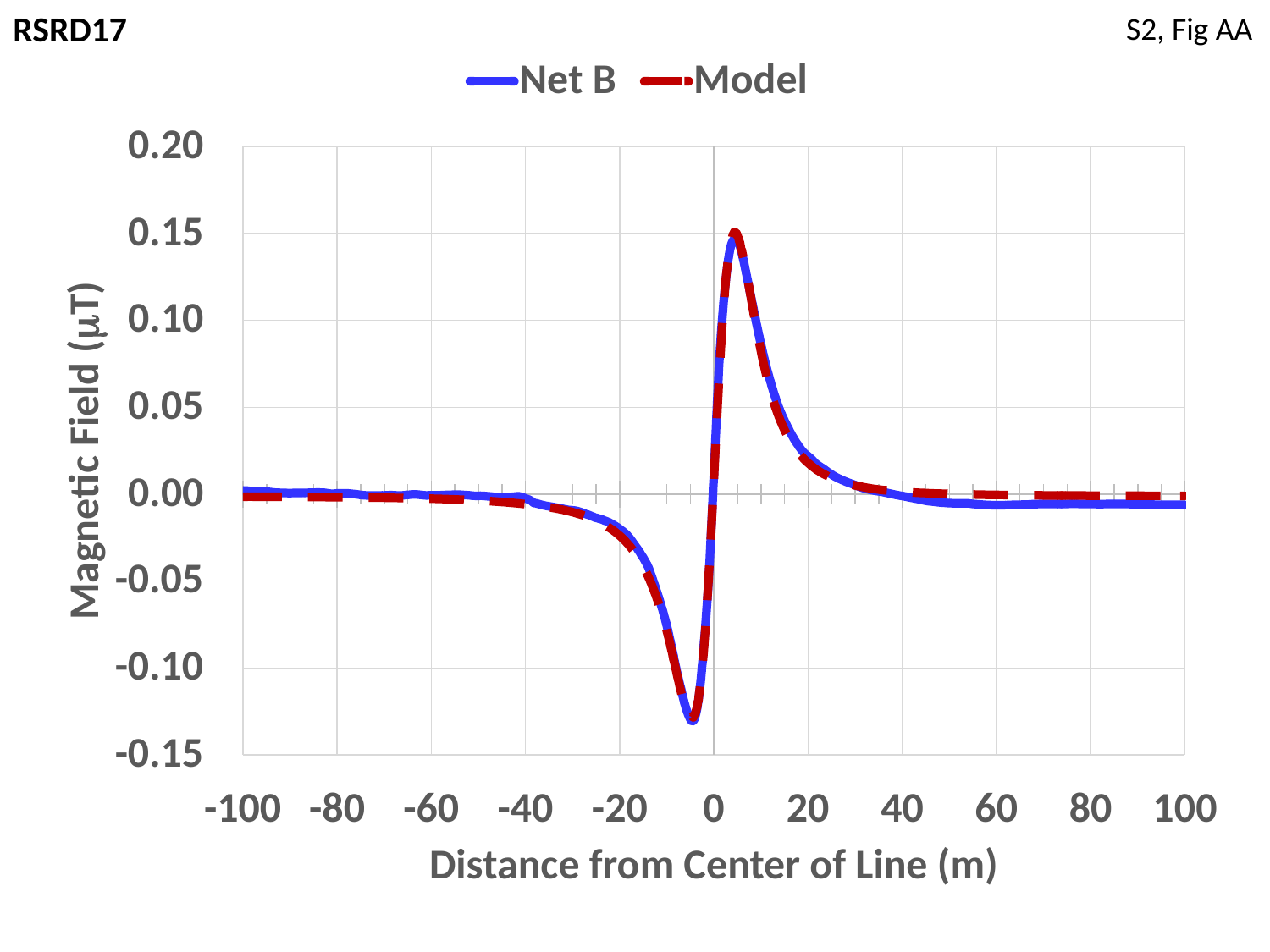

RSRD17
S2, Fig AA

## Slide 29
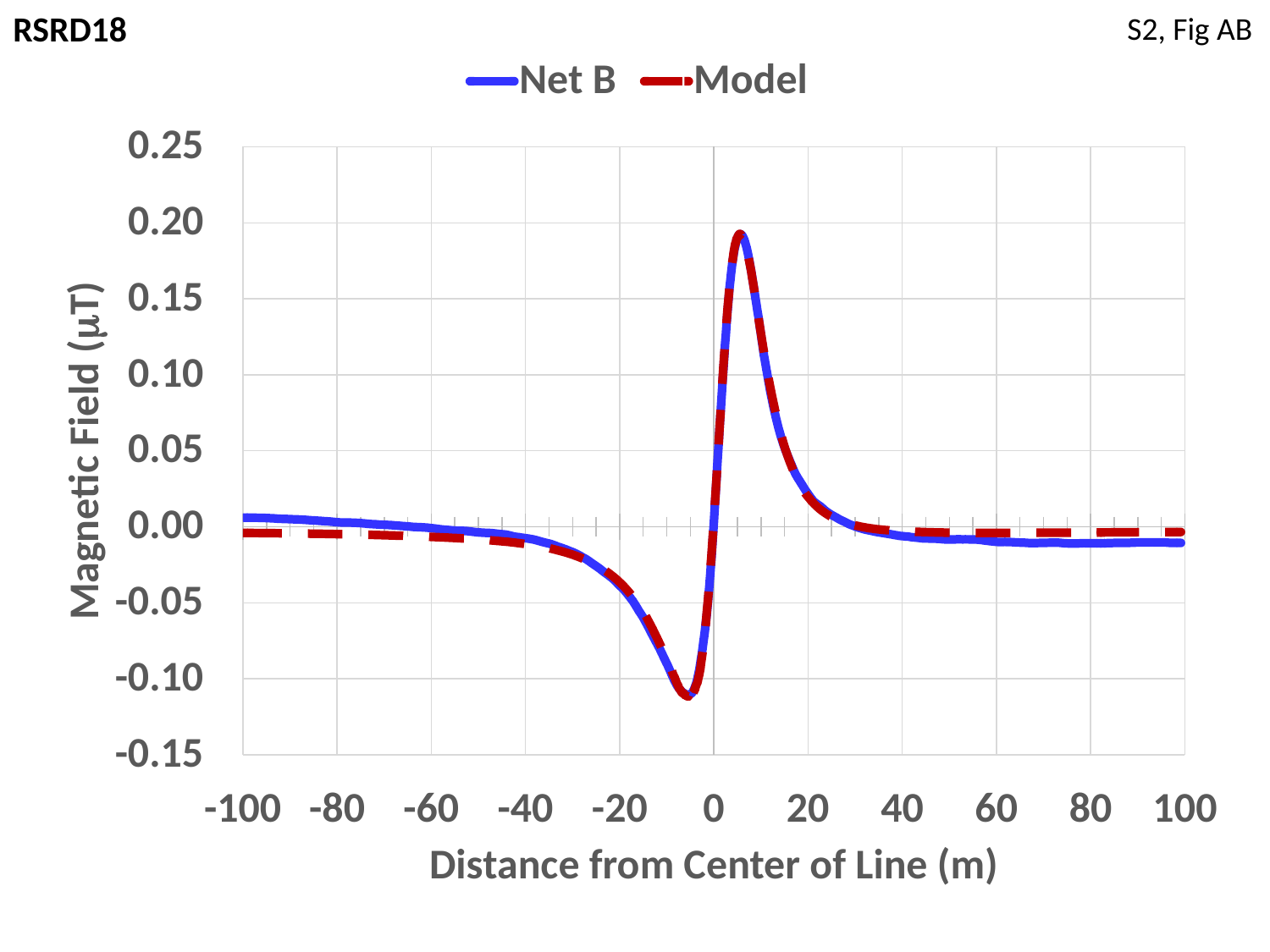

RSRD18
S2, Fig AB

## Slide 30
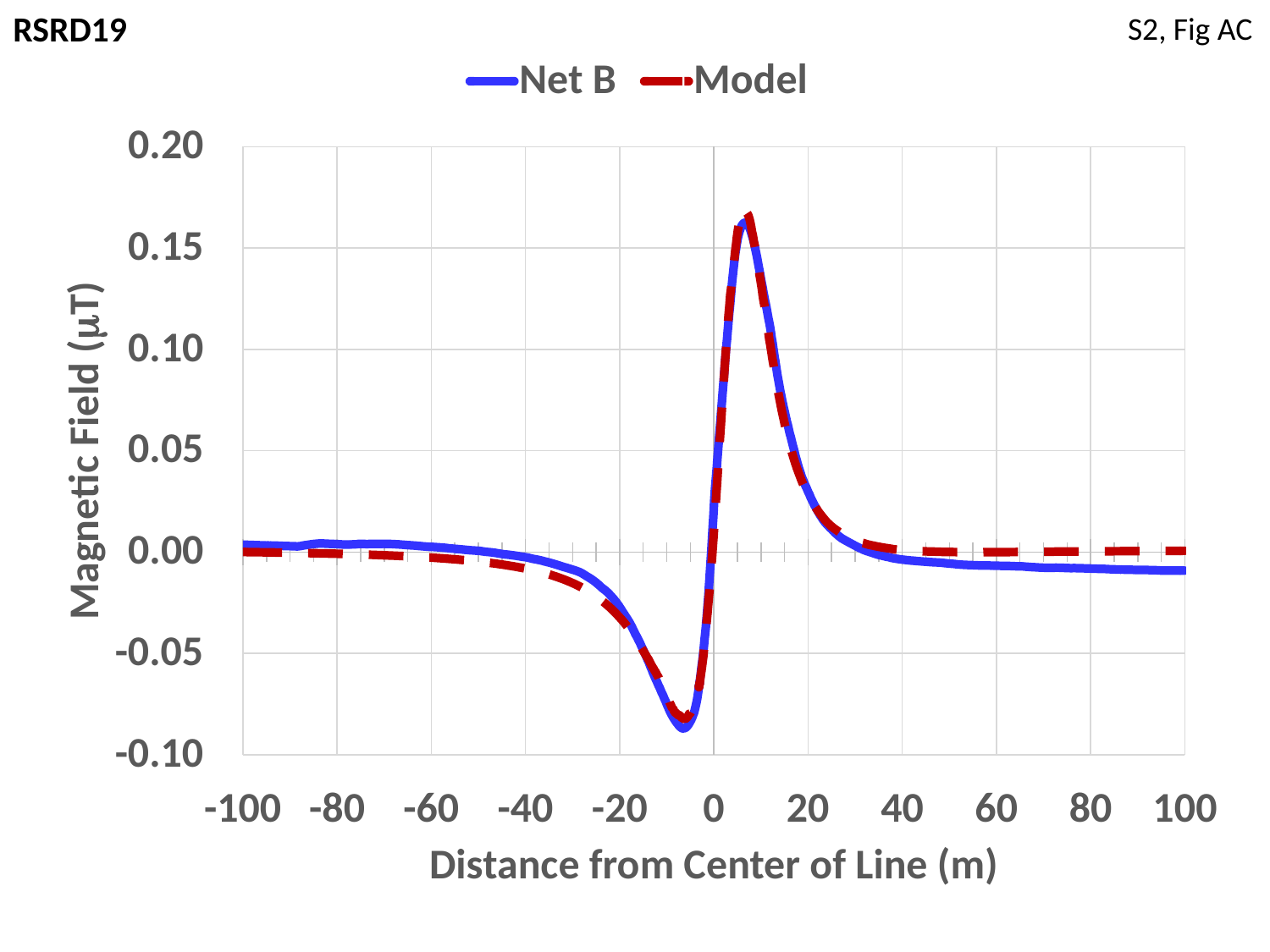

RSRD19
S2, Fig AC

## Slide 31
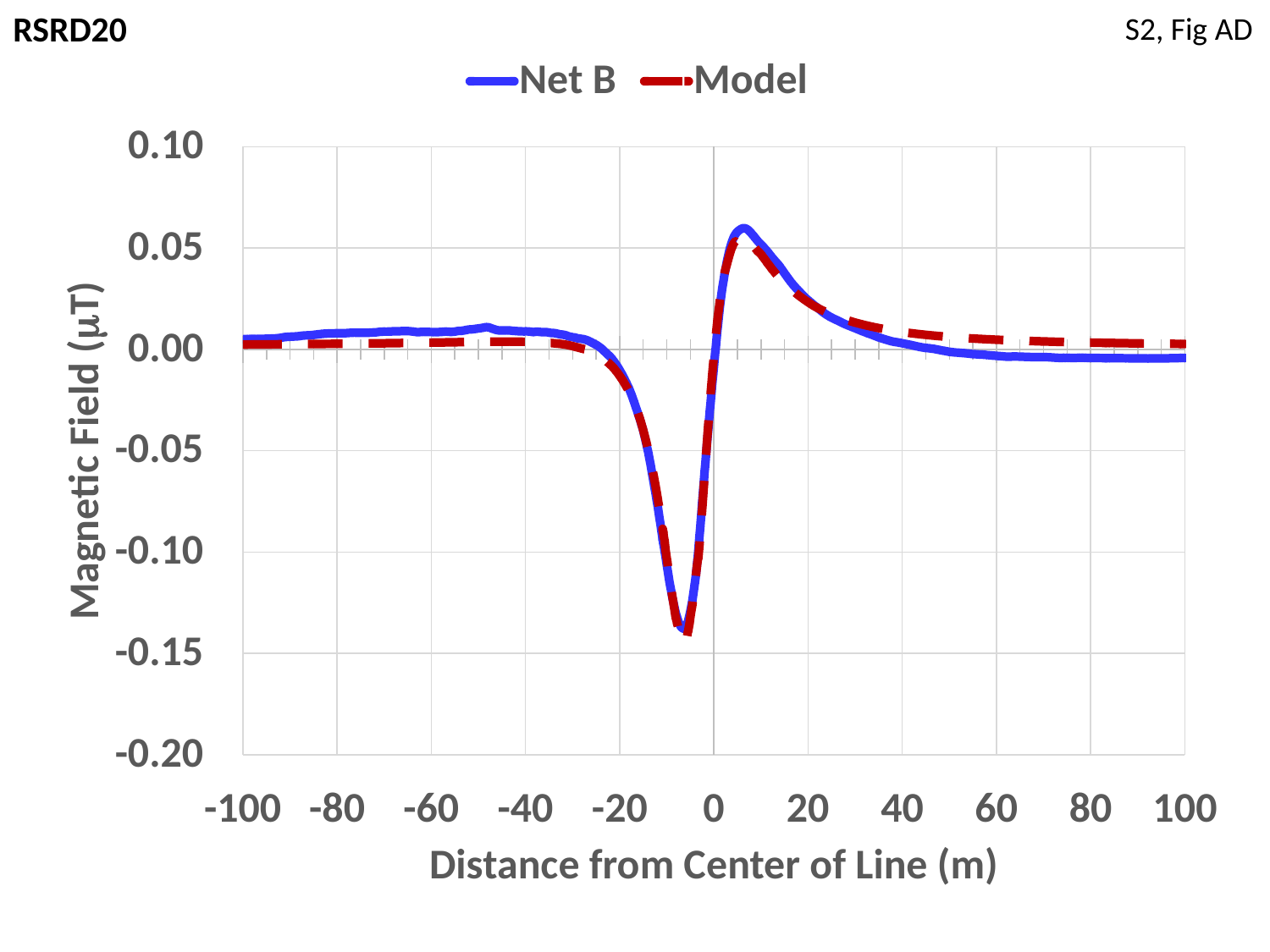

RSRD20
S2, Fig AD

## Slide 32
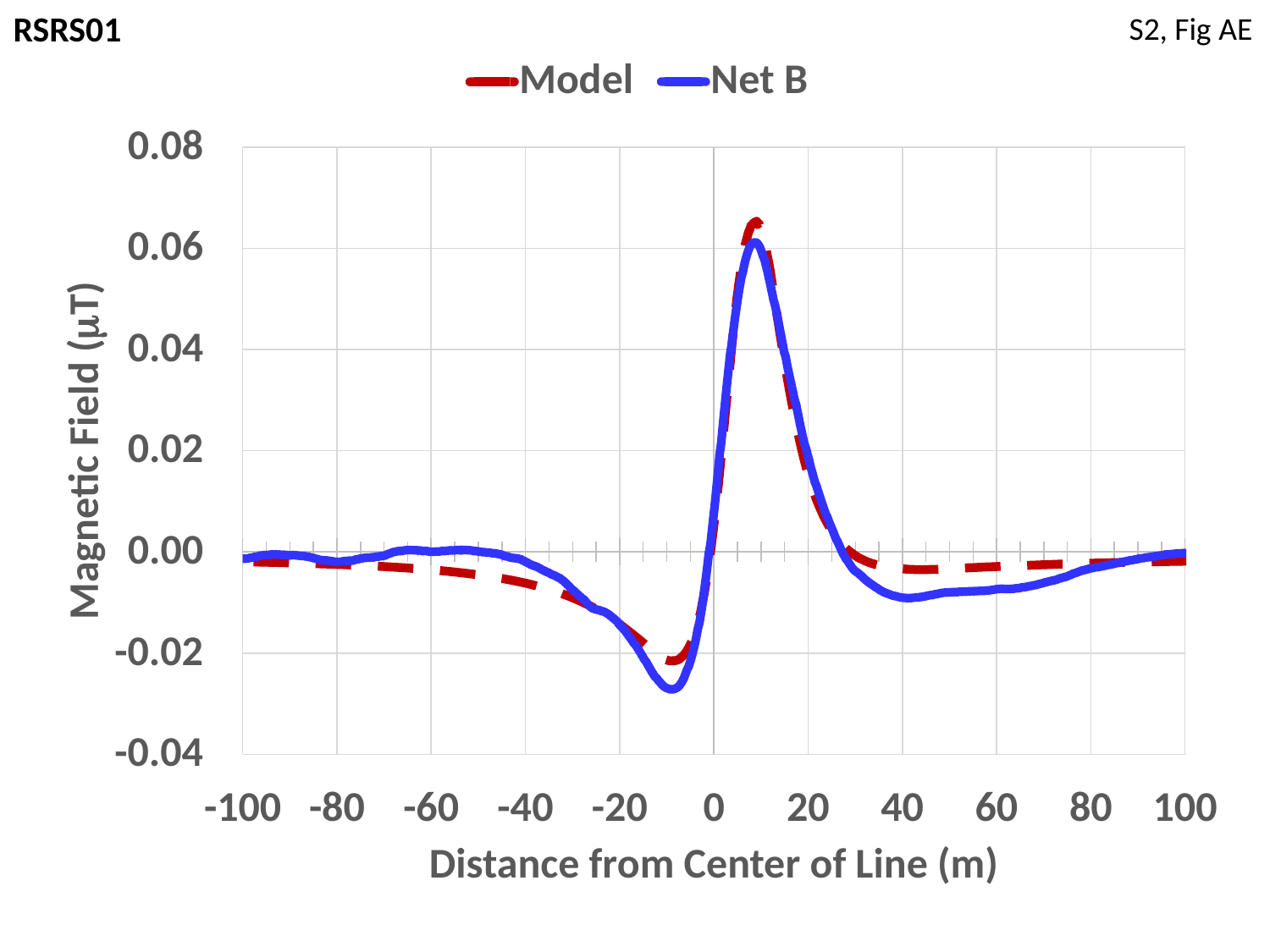

RSRS01
S2, Fig AE

## Slide 33
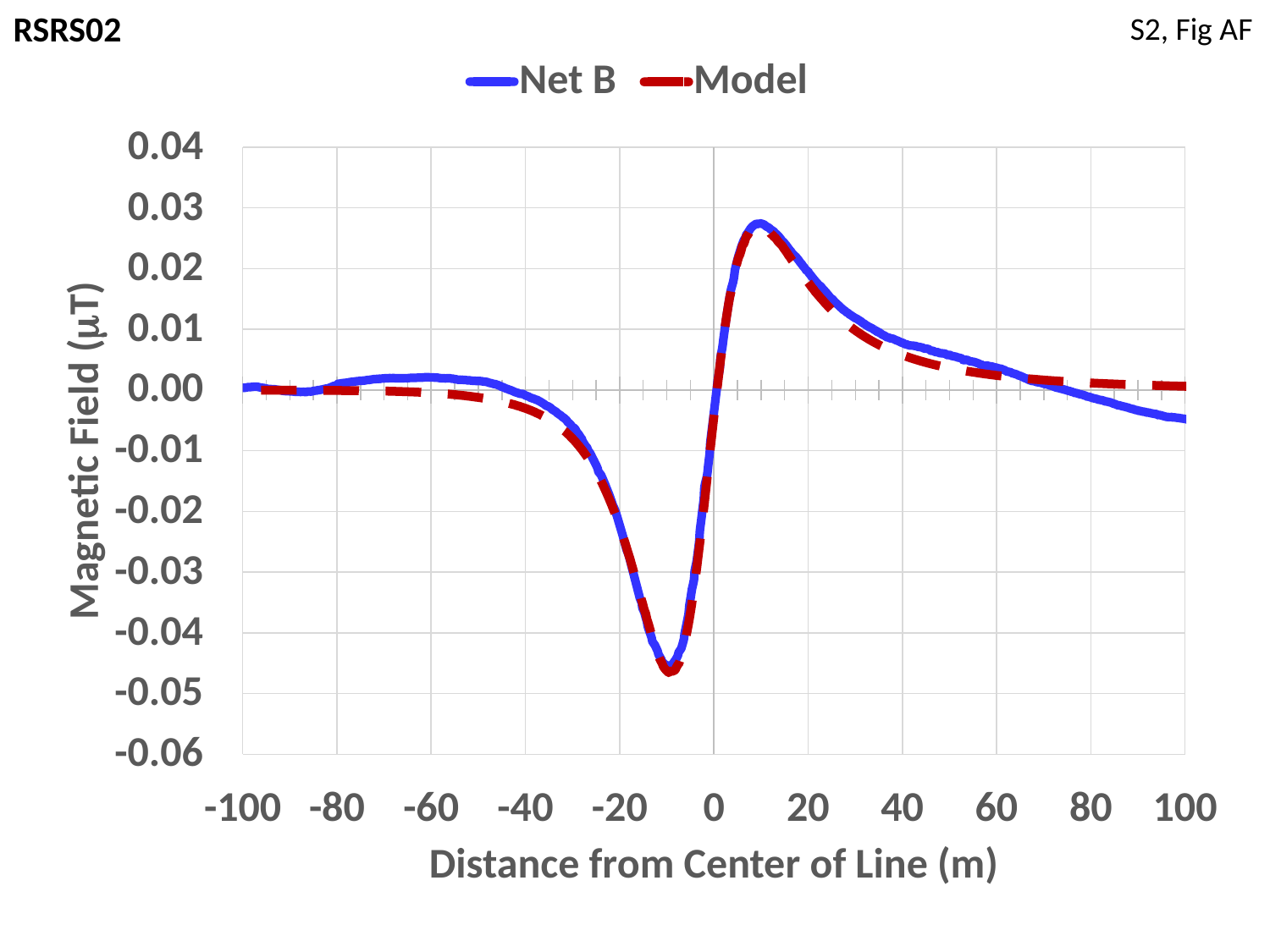

RSRS02
S2, Fig AF

## Slide 34
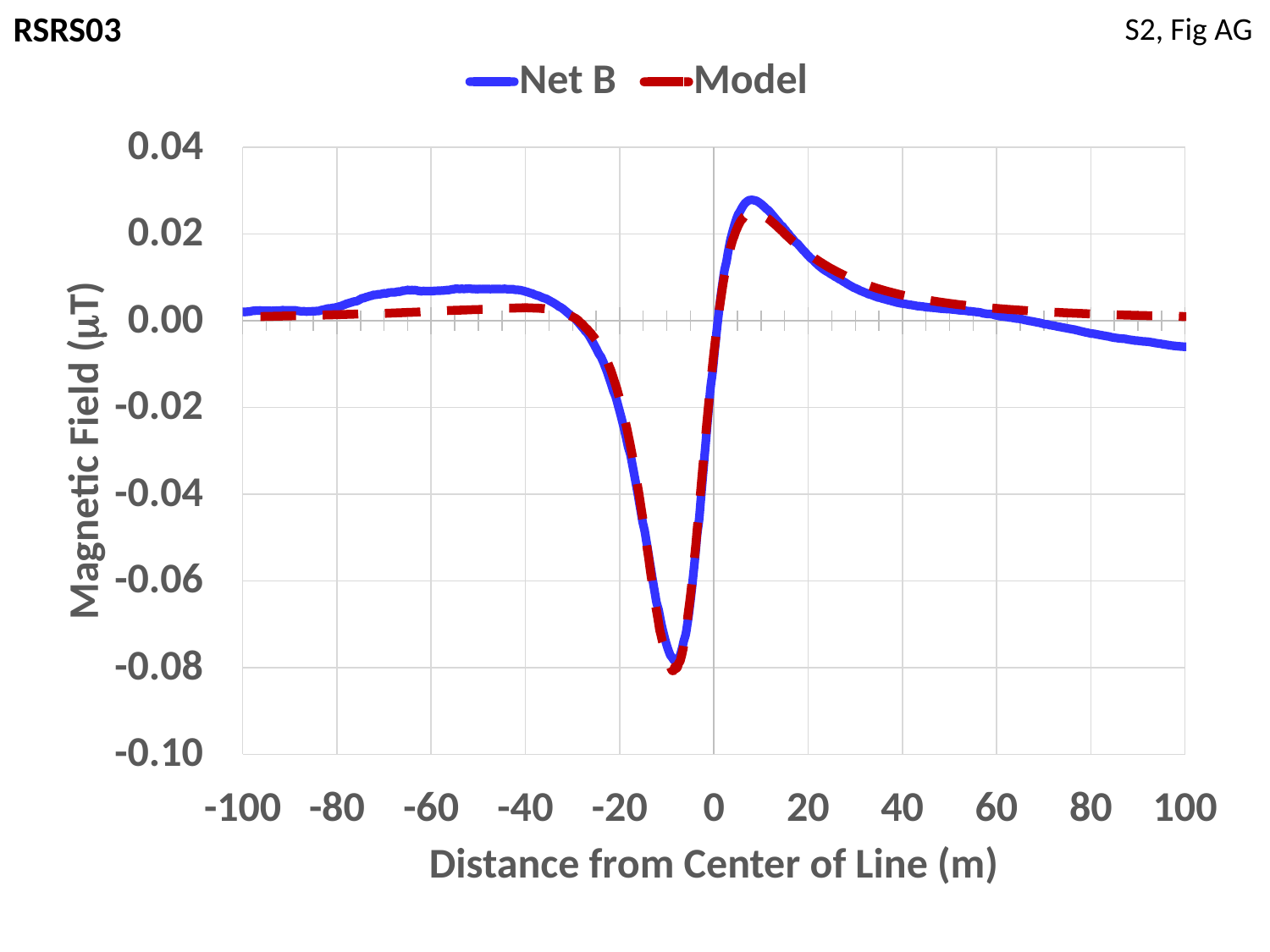

RSRS03
S2, Fig AG

## Slide 35
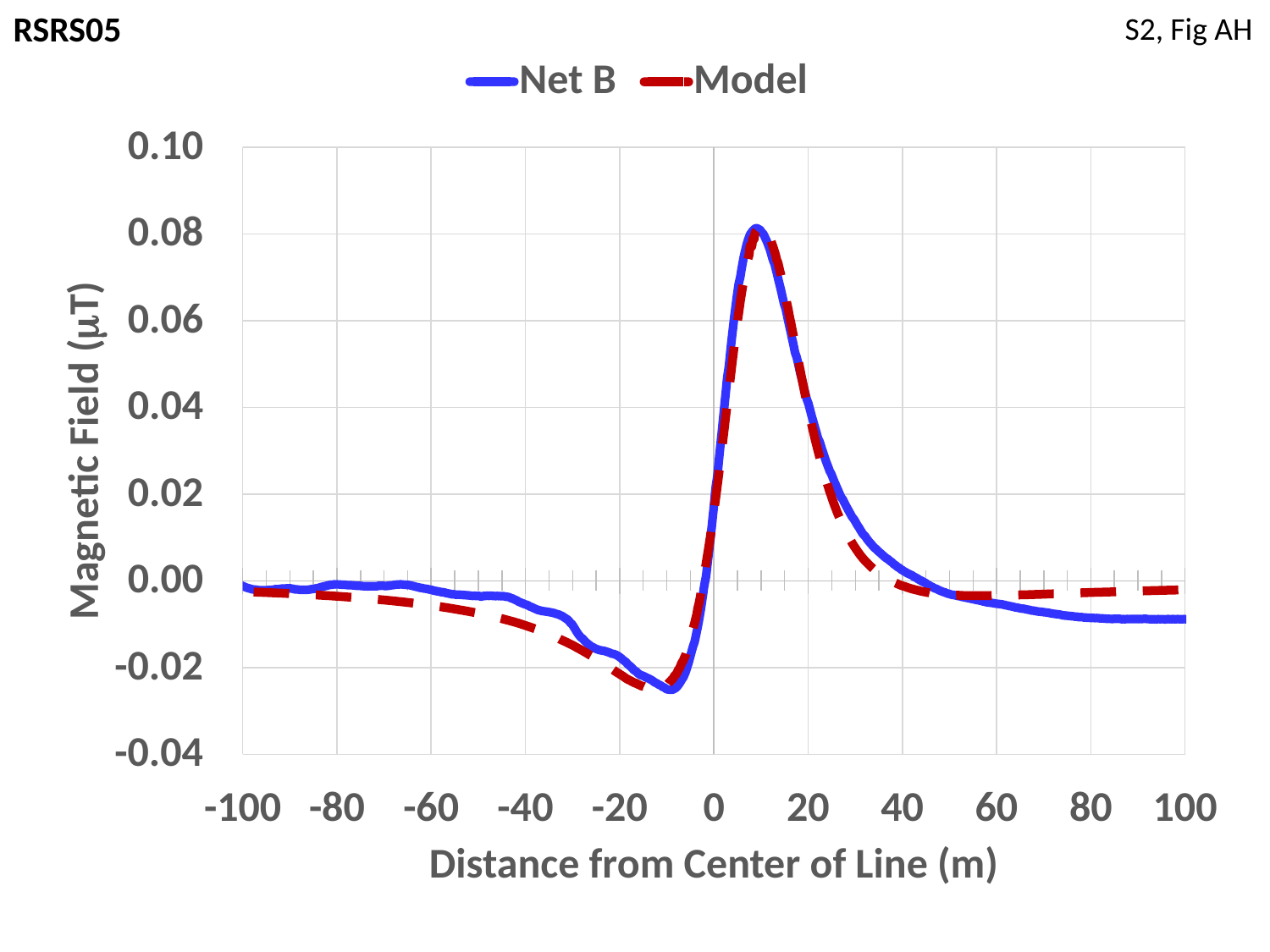

RSRS05
S2, Fig AH

## Slide 36
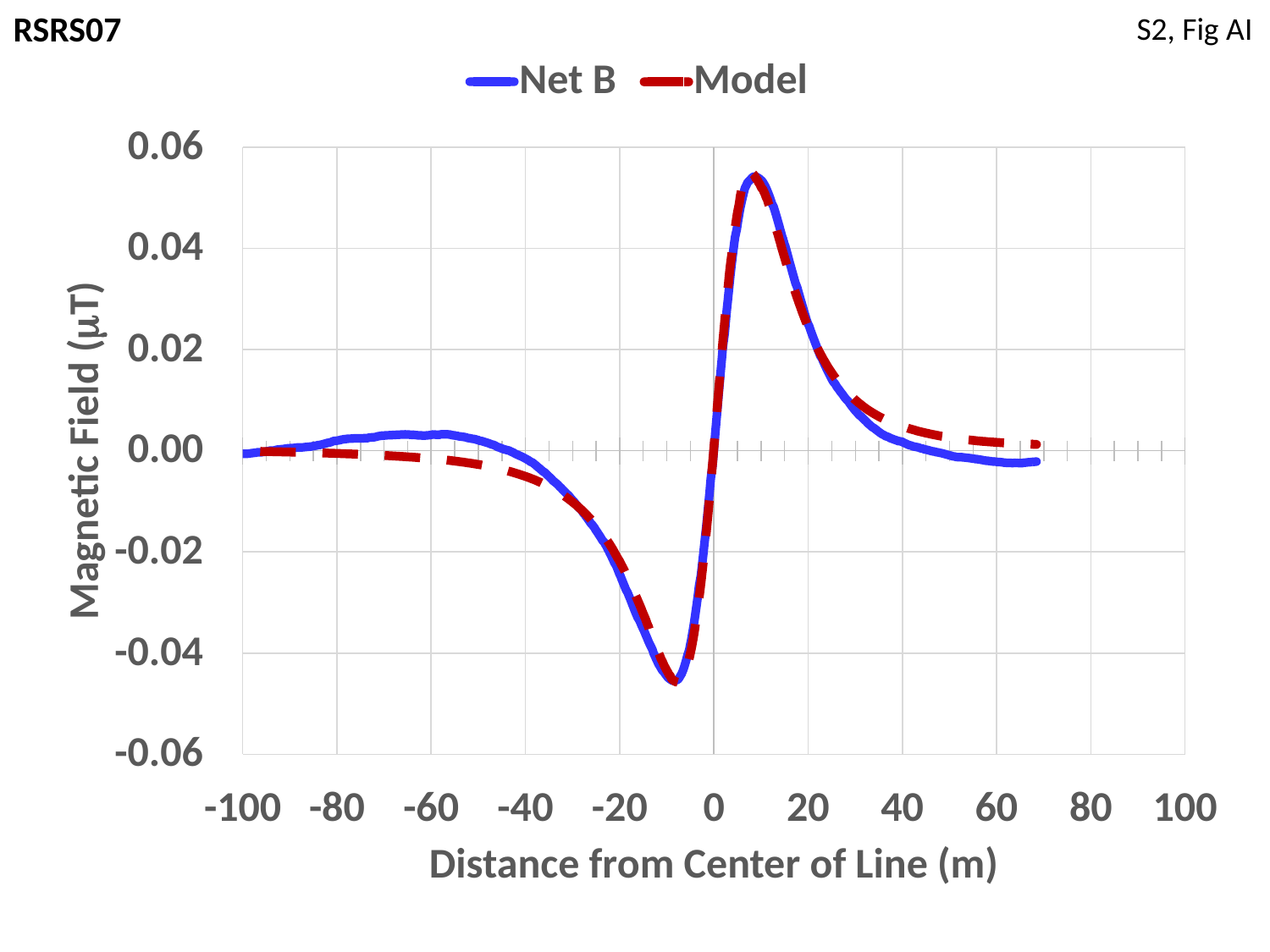

RSRS07
S2, Fig AI

## Slide 37
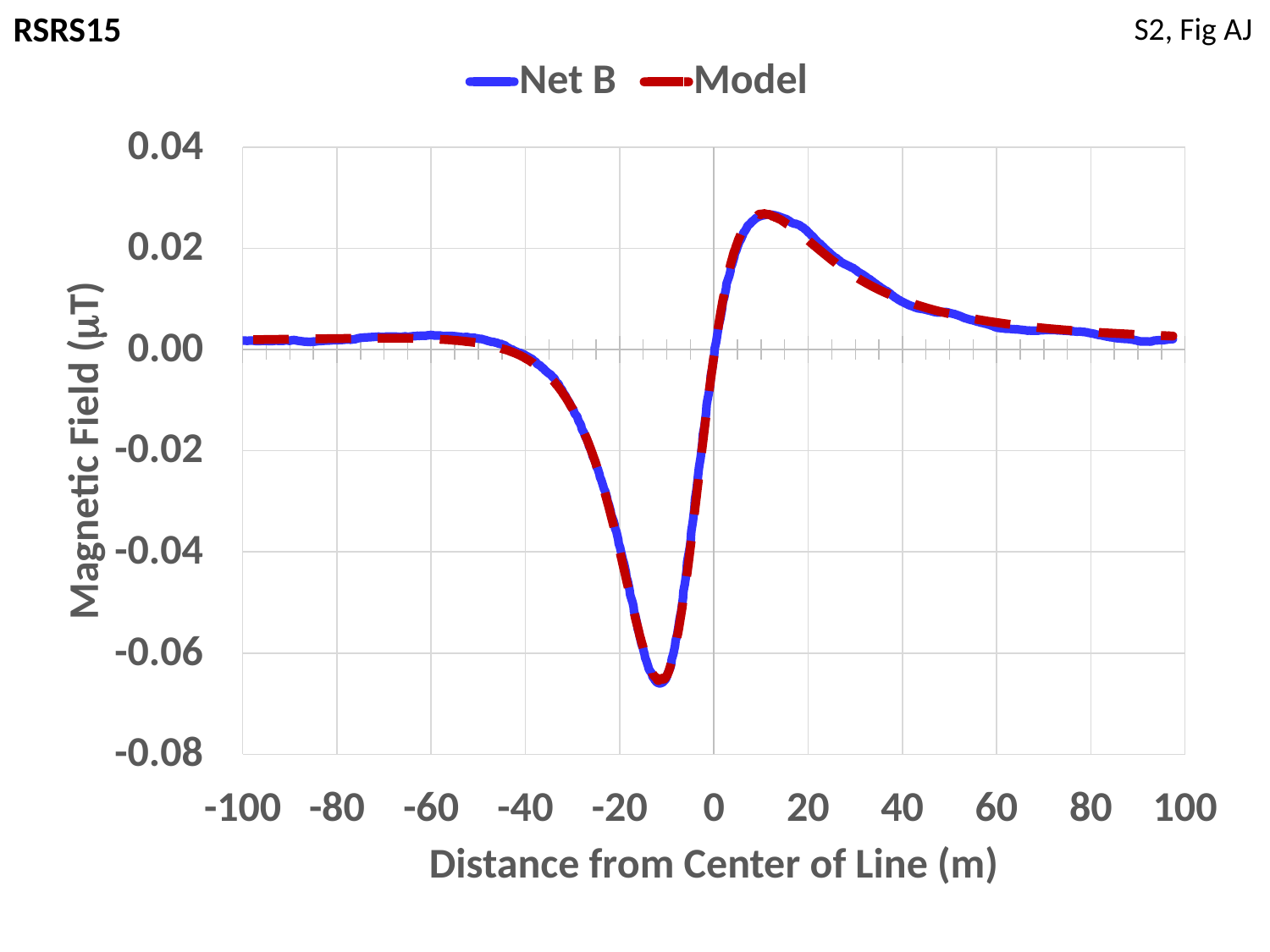

RSRS15
S2, Fig AJ

## Slide 38
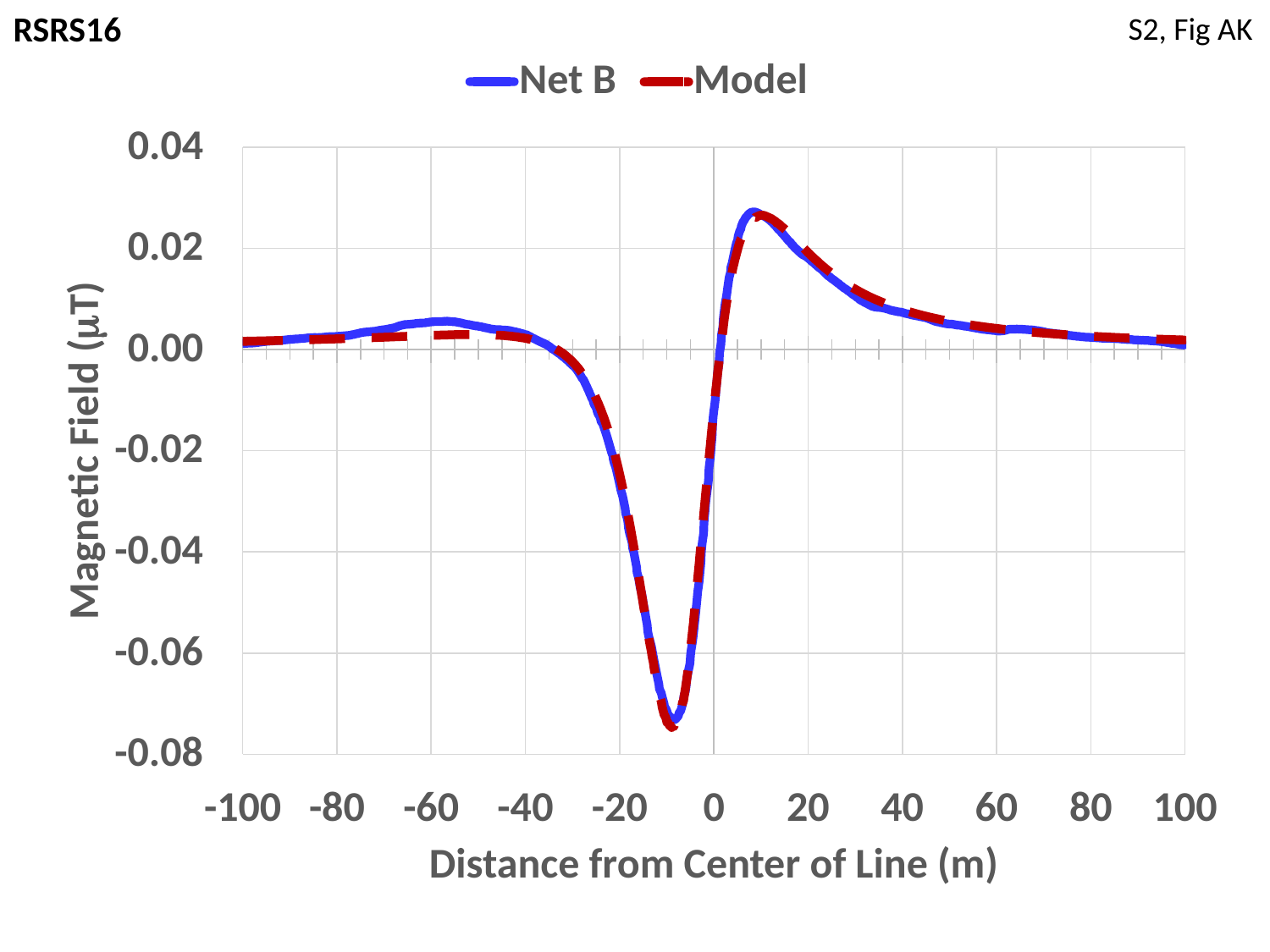

RSRS16
S2, Fig AK

## Slide 39
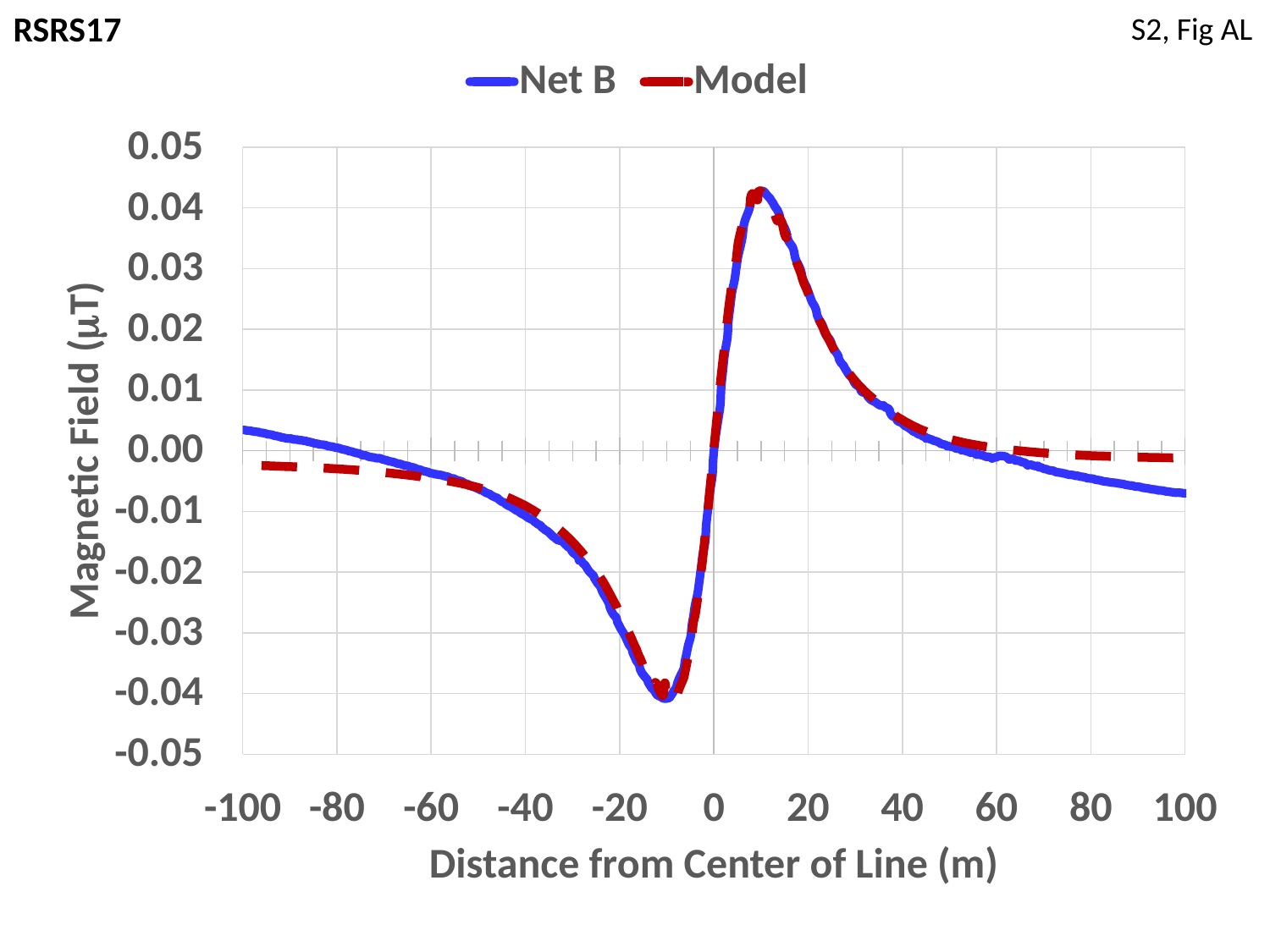

RSRS17
S2, Fig AL

## Slide 40
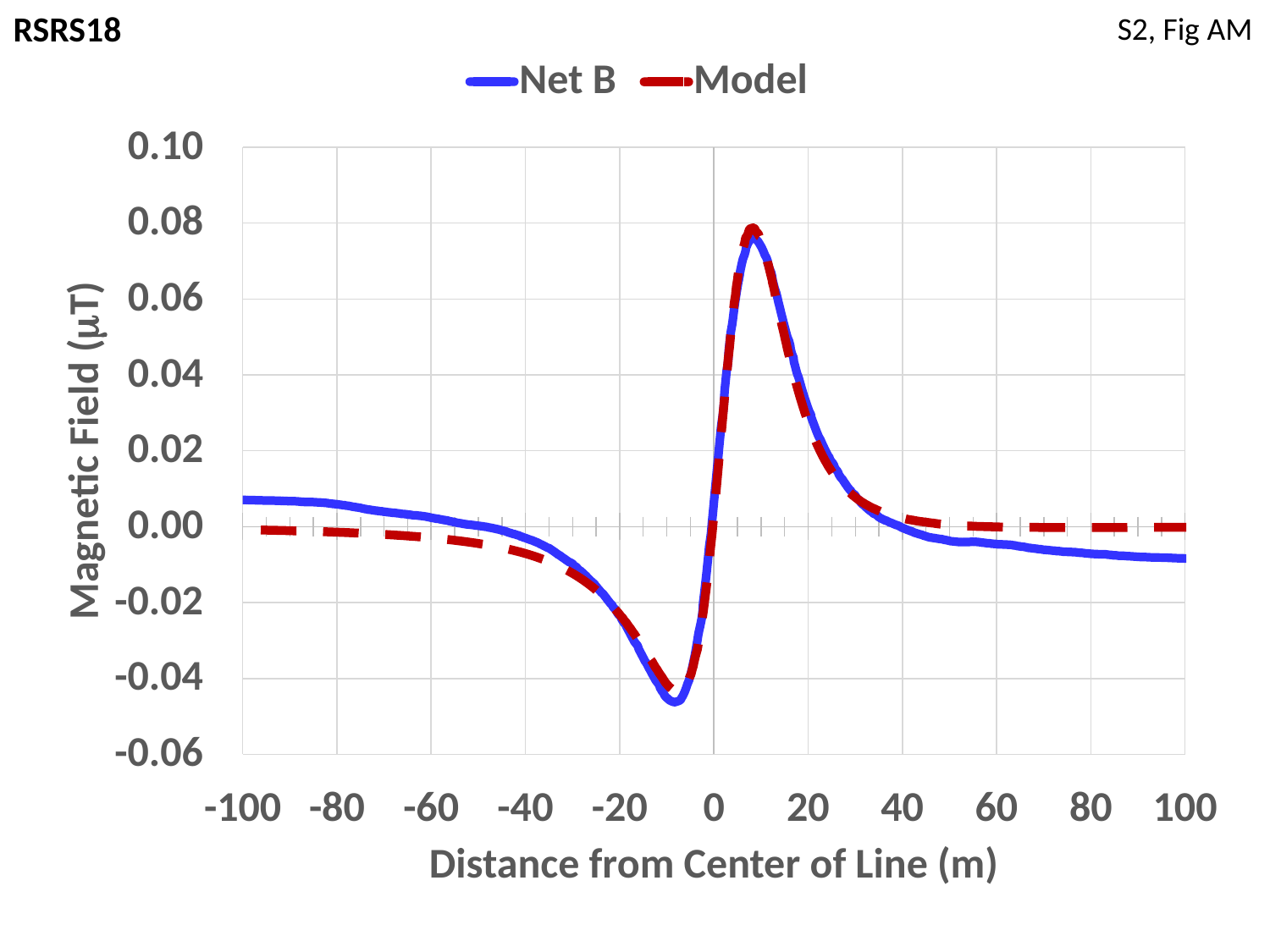

RSRS18
S2, Fig AM

## Slide 41
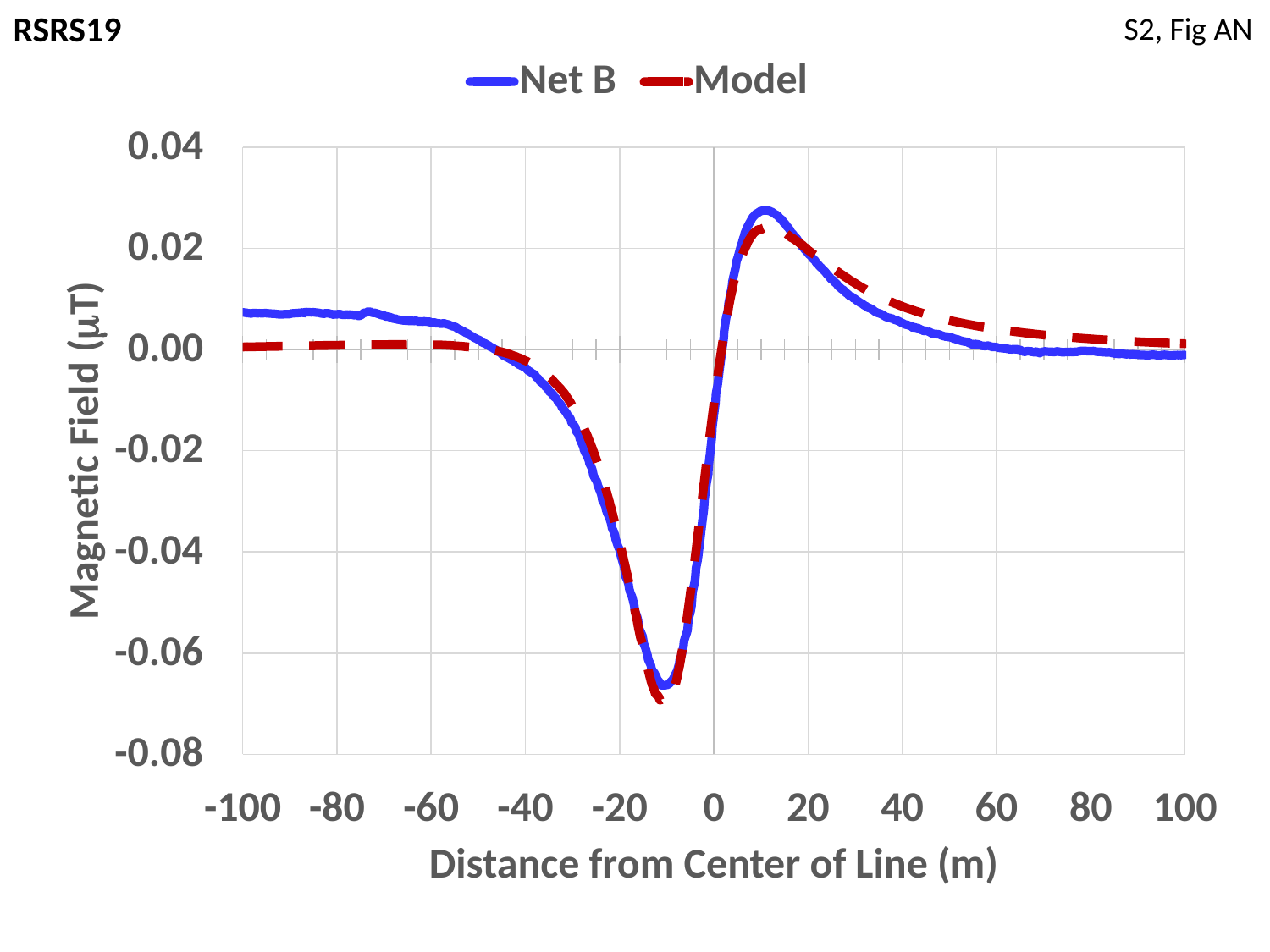

RSRS19
S2, Fig AN

## Slide 42
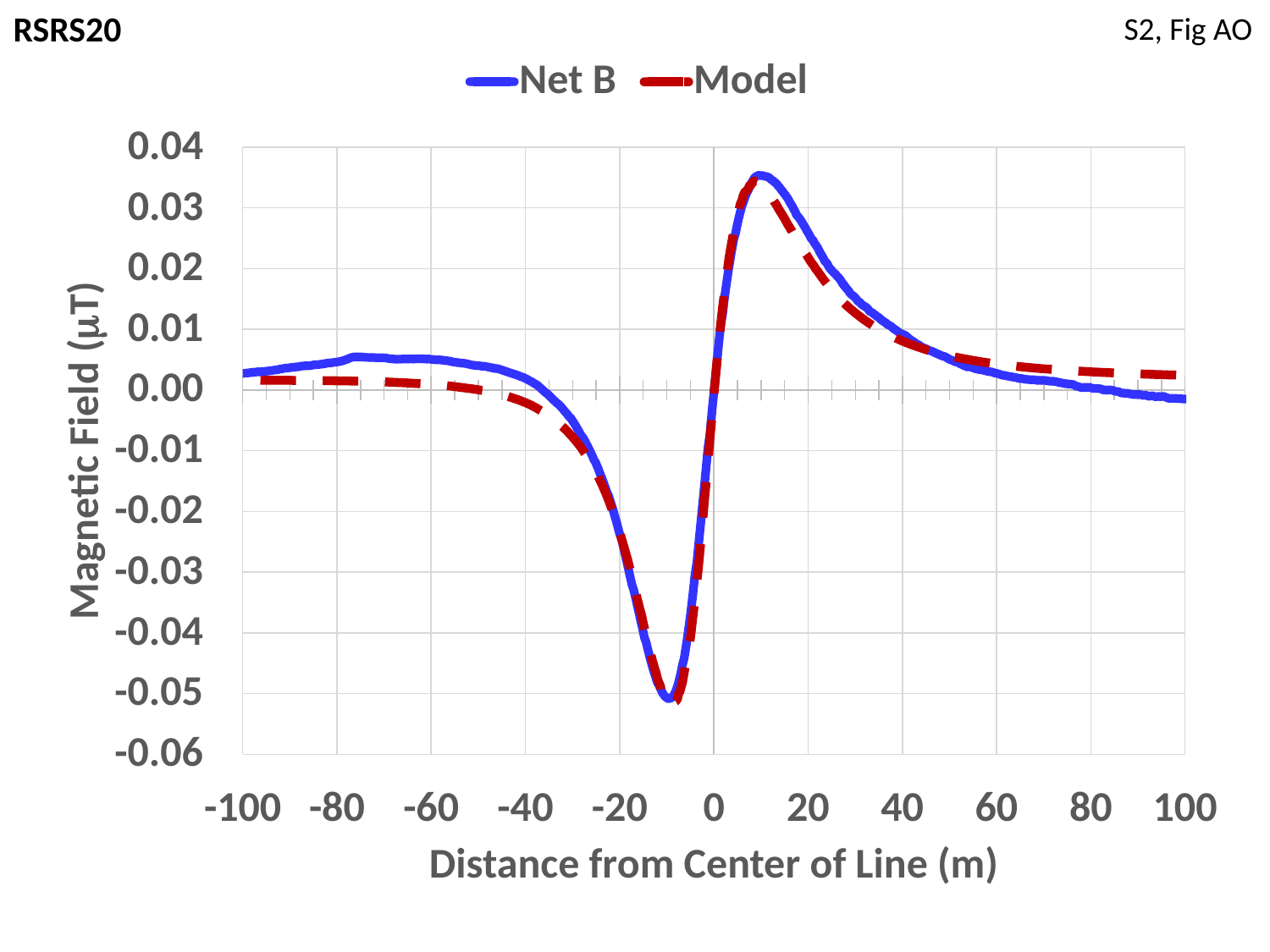

RSRS20
S2, Fig AO

## Slide 43
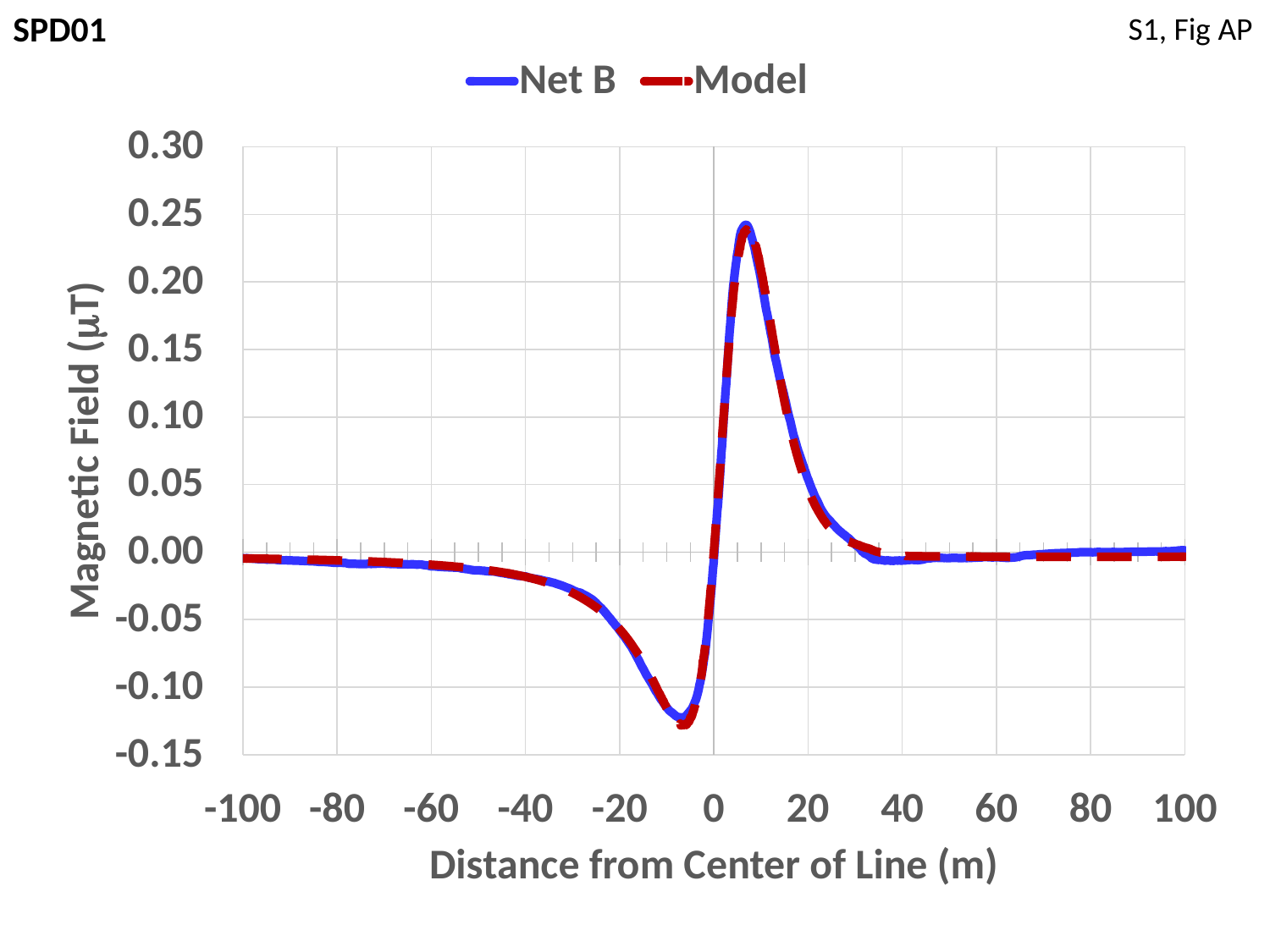

SPD01
S1, Fig AP

## Slide 44
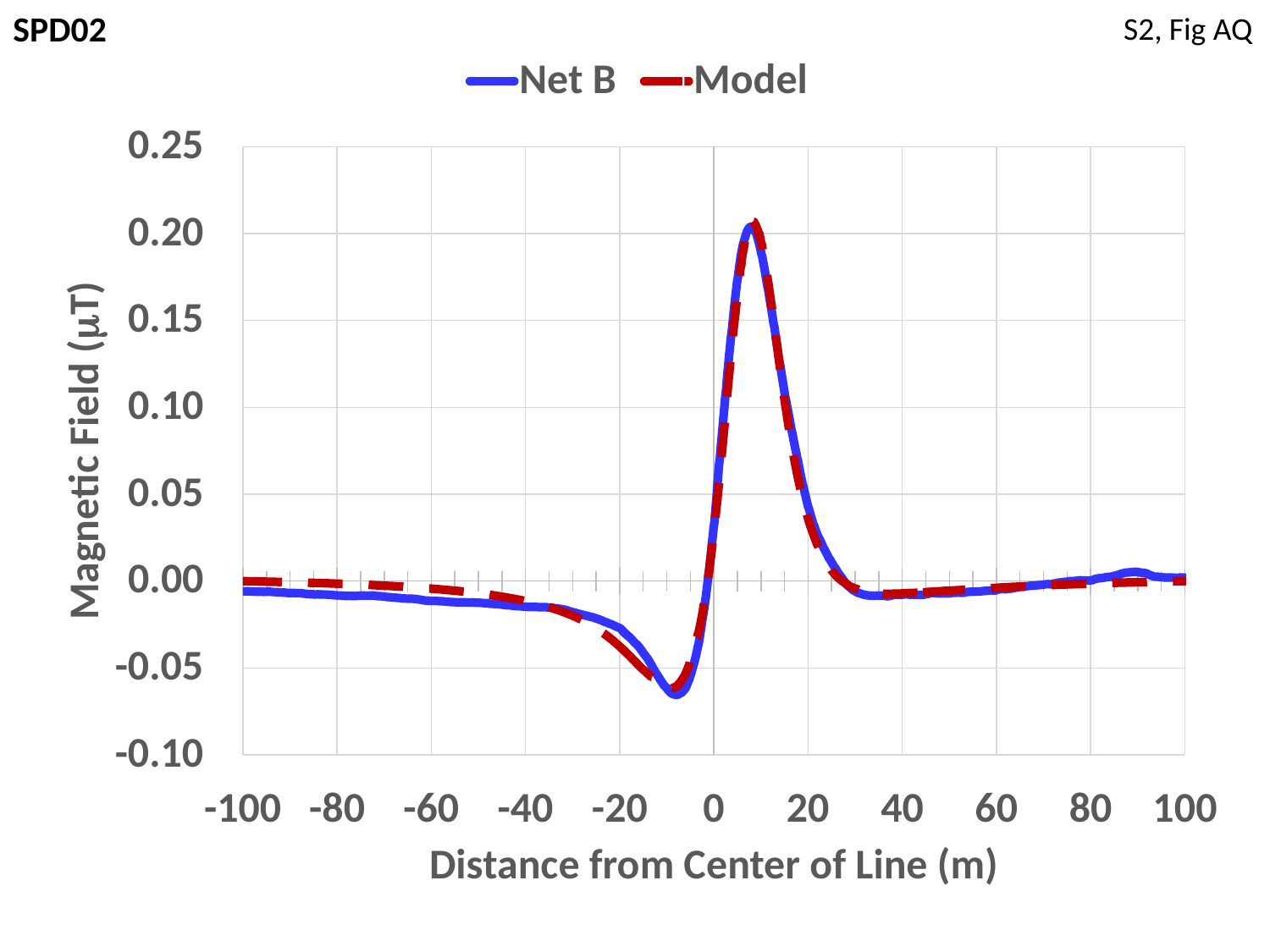

SPD02
S2, Fig AQ

## Slide 45
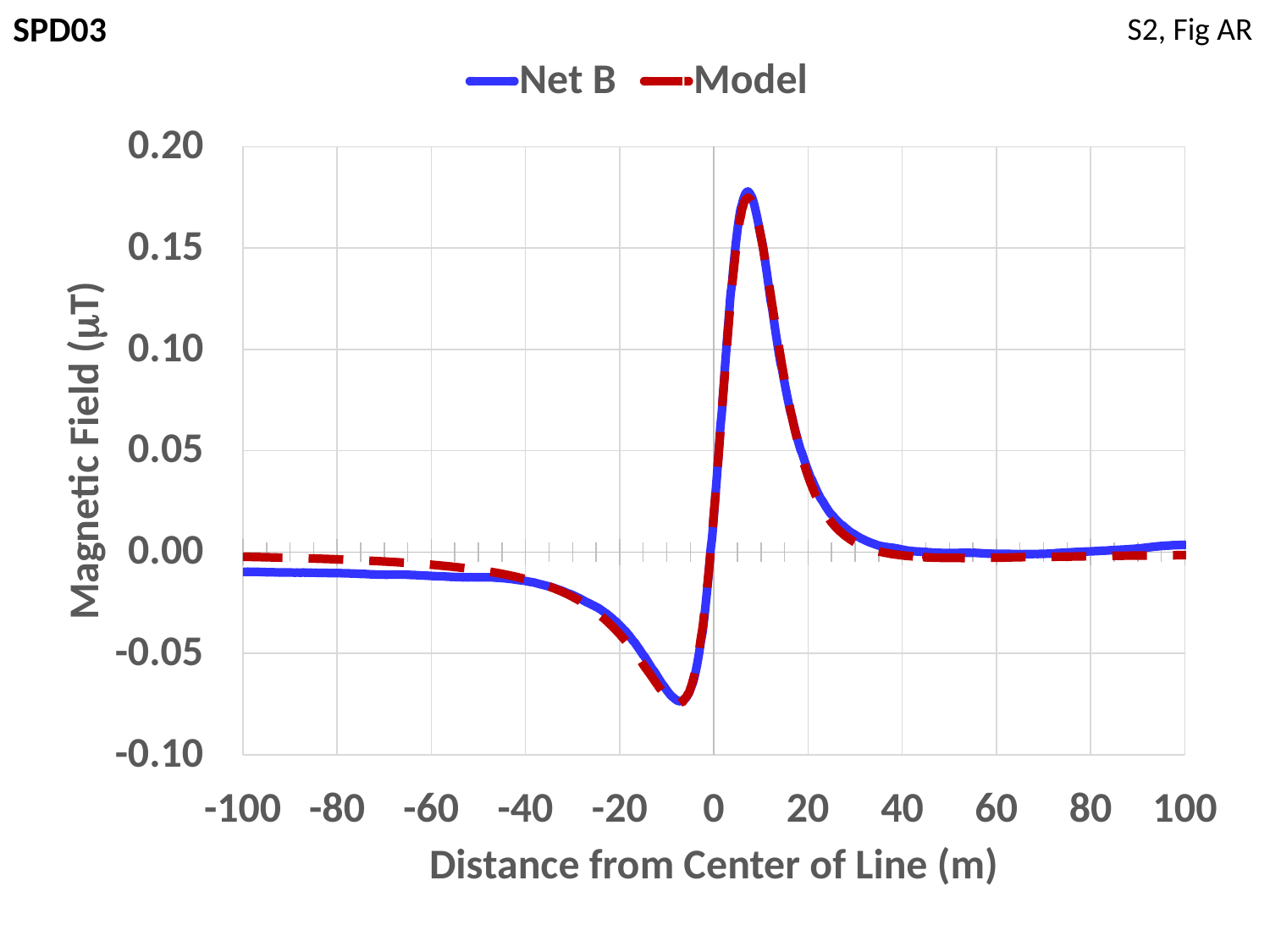

SPD03
S2, Fig AR

## Slide 46
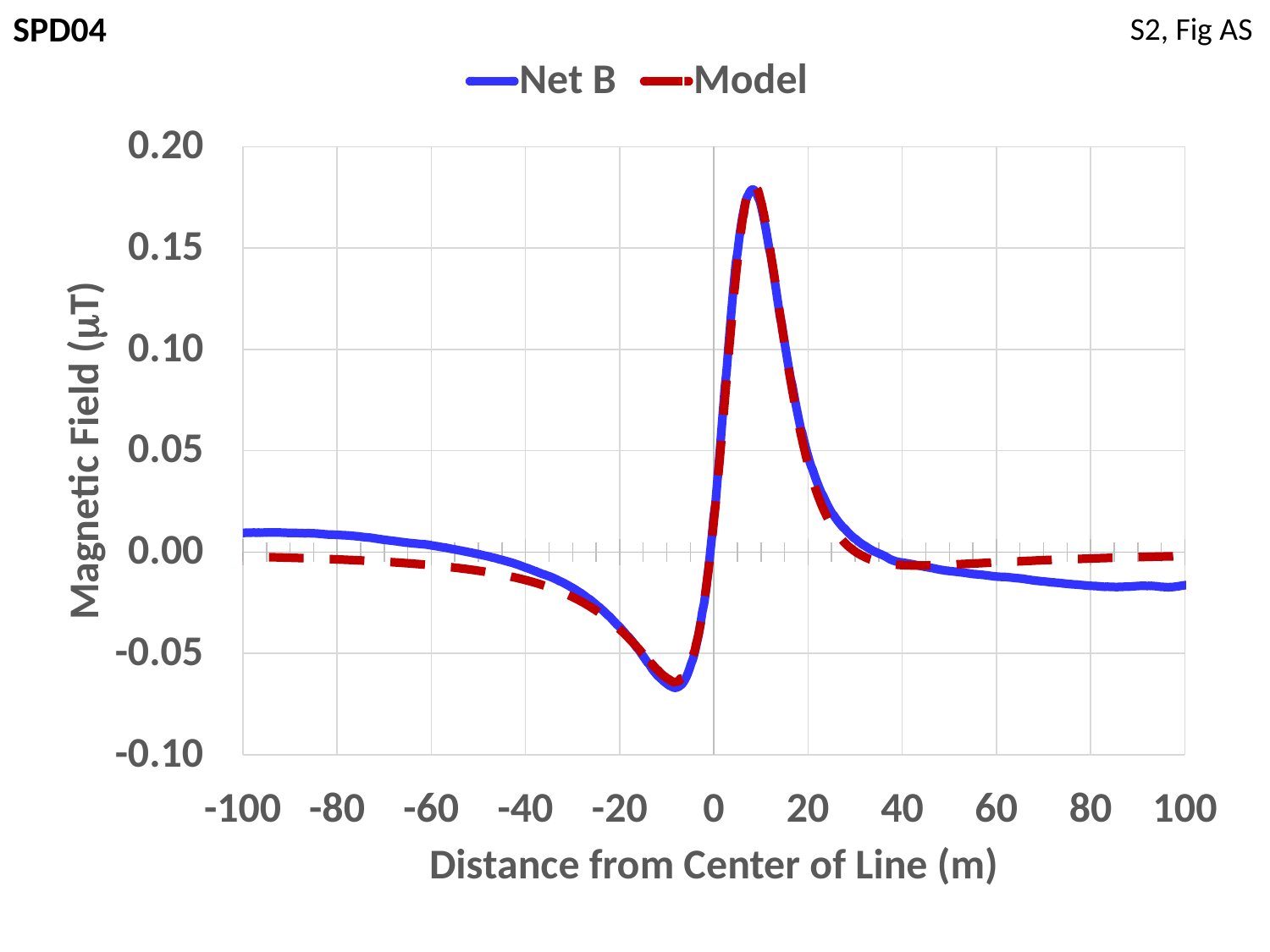

SPD04
S2, Fig AS

## Slide 47
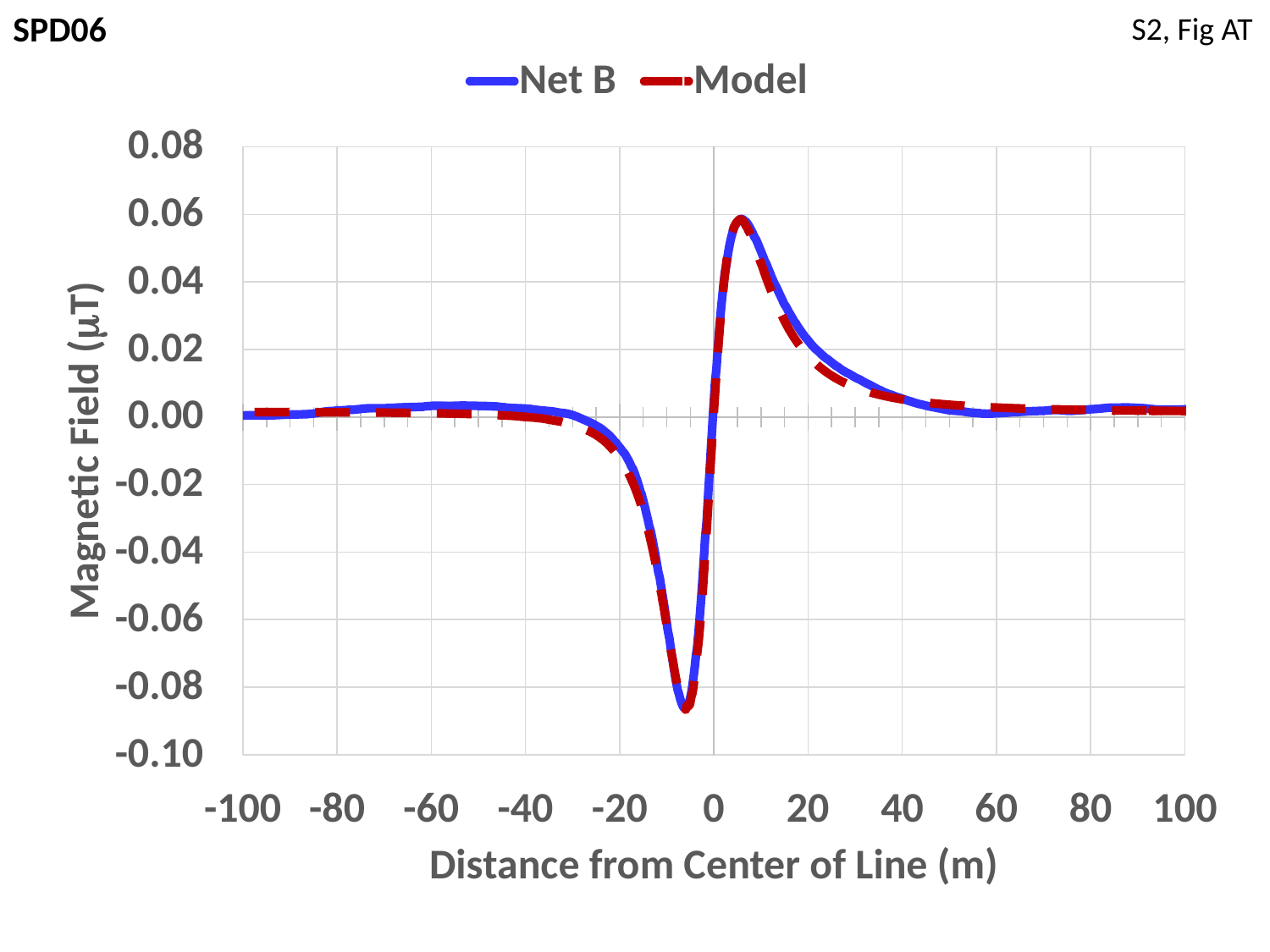

SPD06
S2, Fig AT

## Slide 48
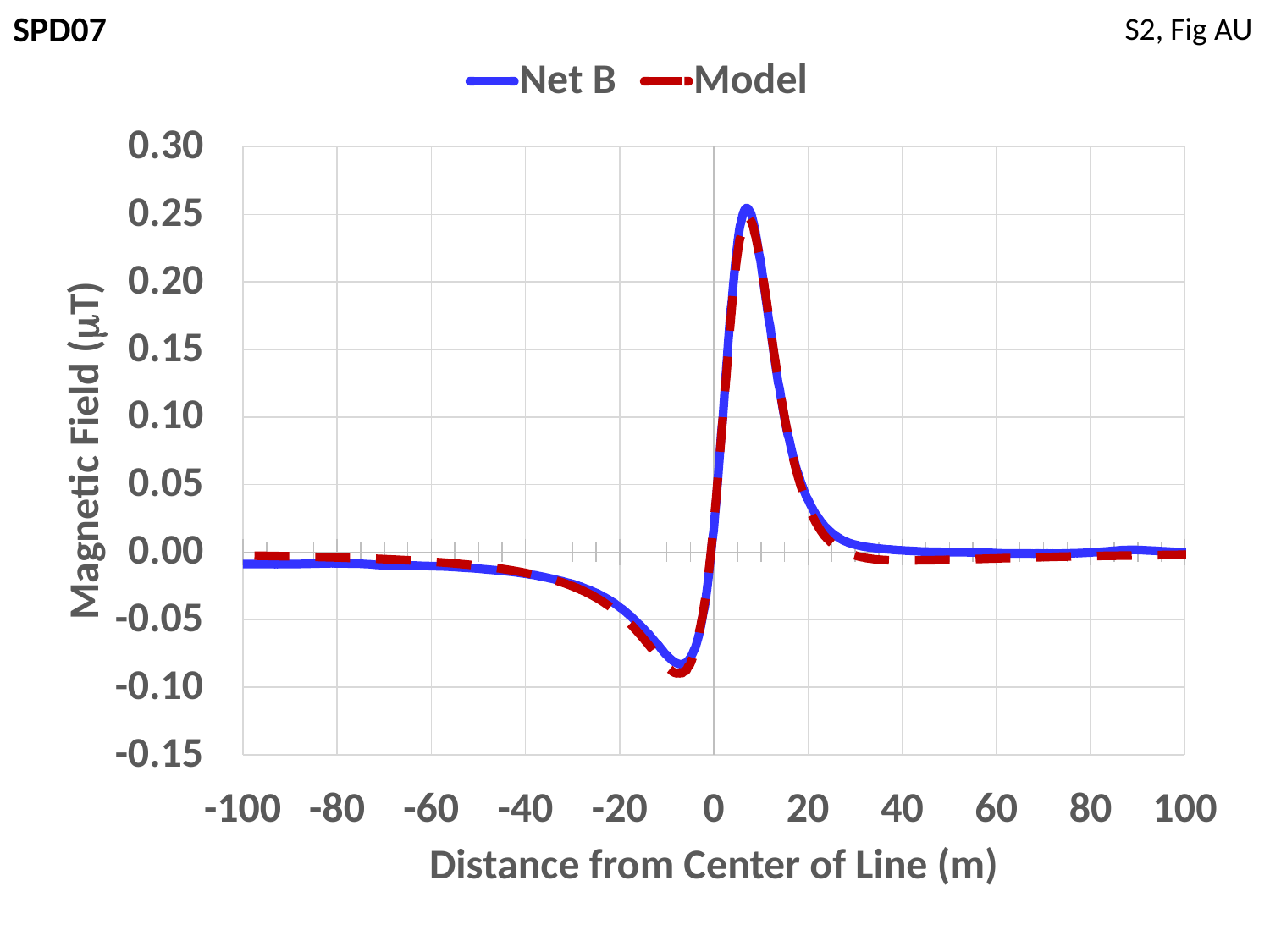

SPD07
S2, Fig AU

## Slide 49
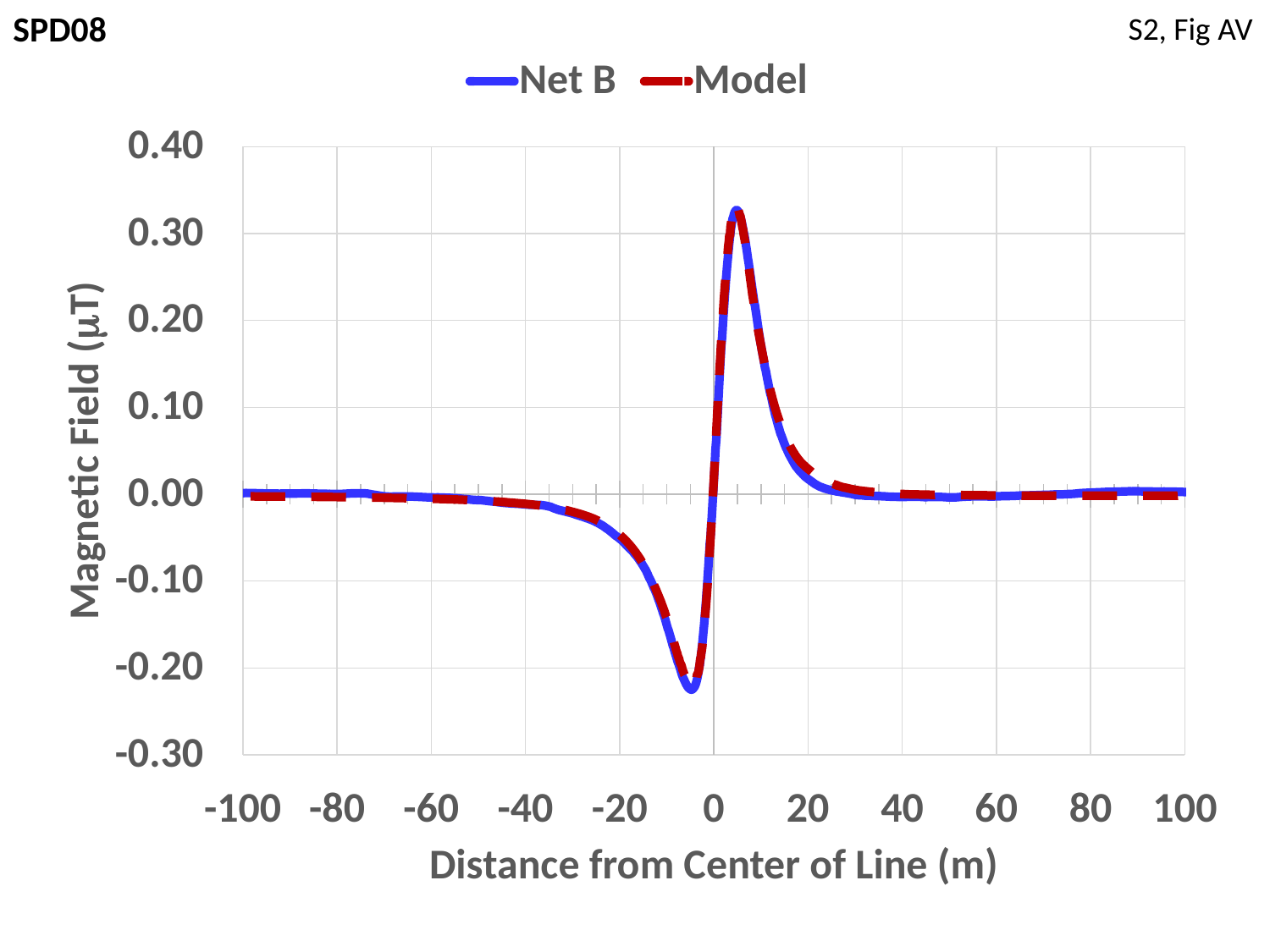

SPD08
S2, Fig AV

## Slide 50
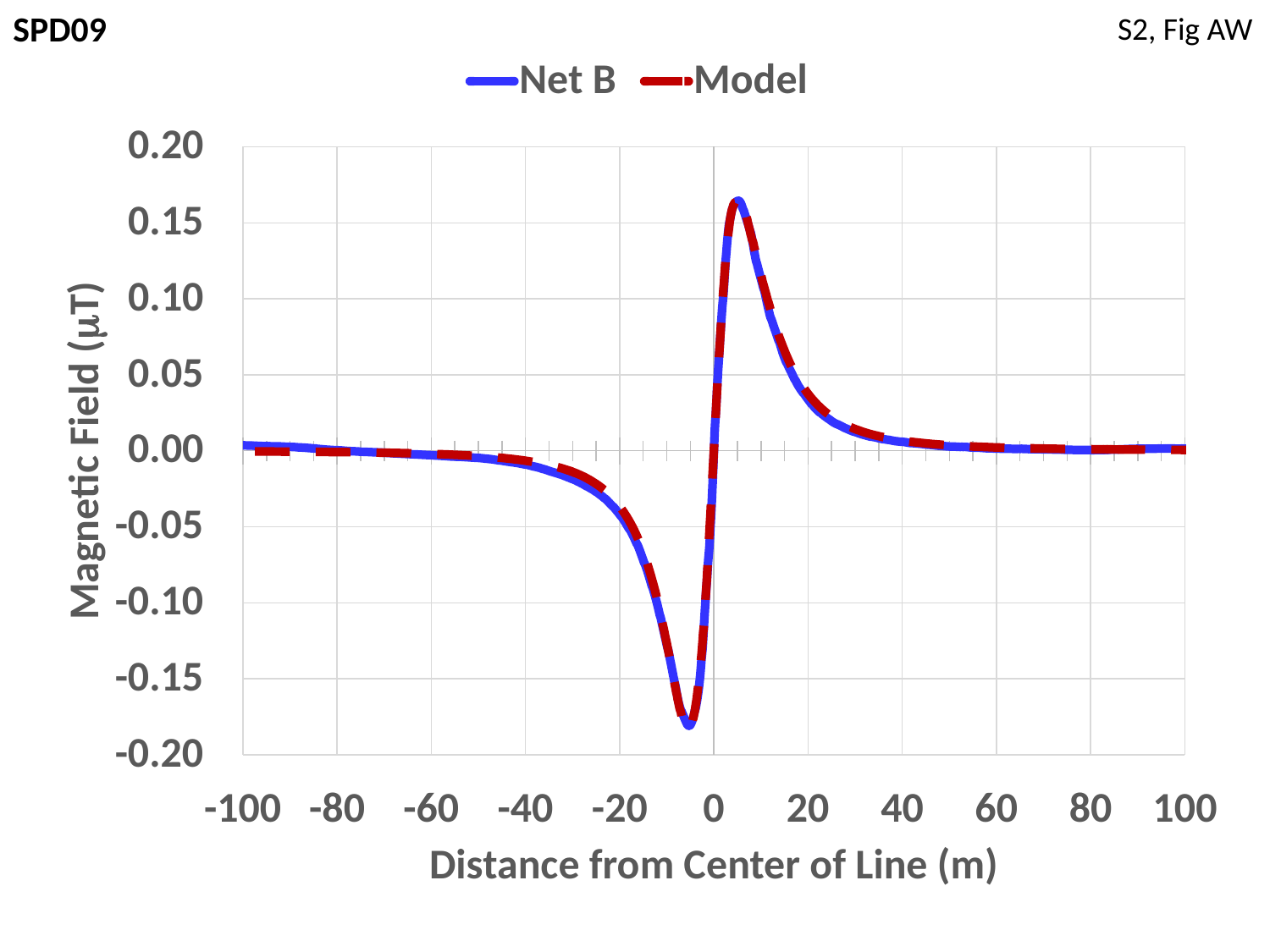

SPD09
S2, Fig AW

## Slide 51
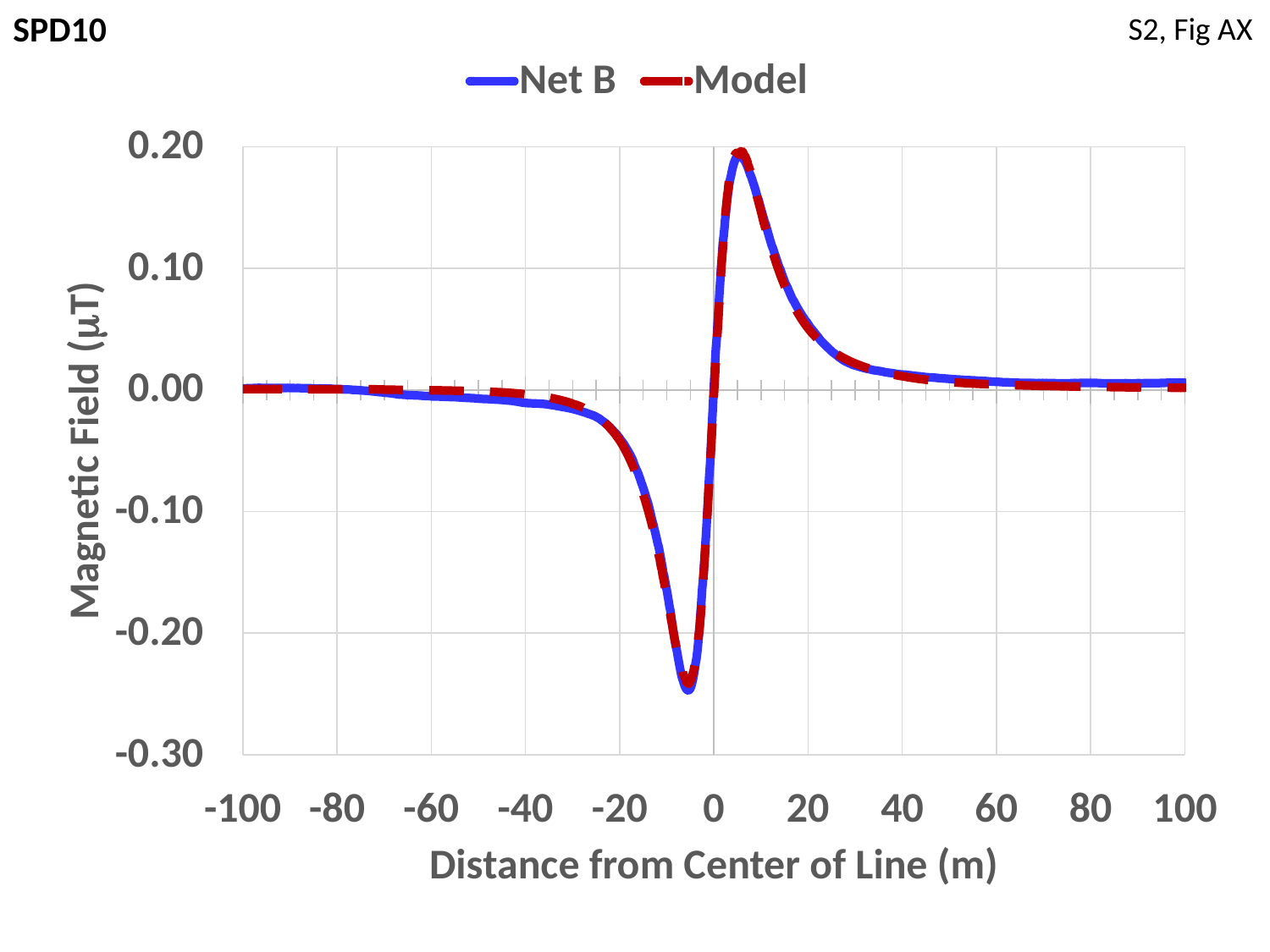

SPD10
S2, Fig AX

## Slide 52
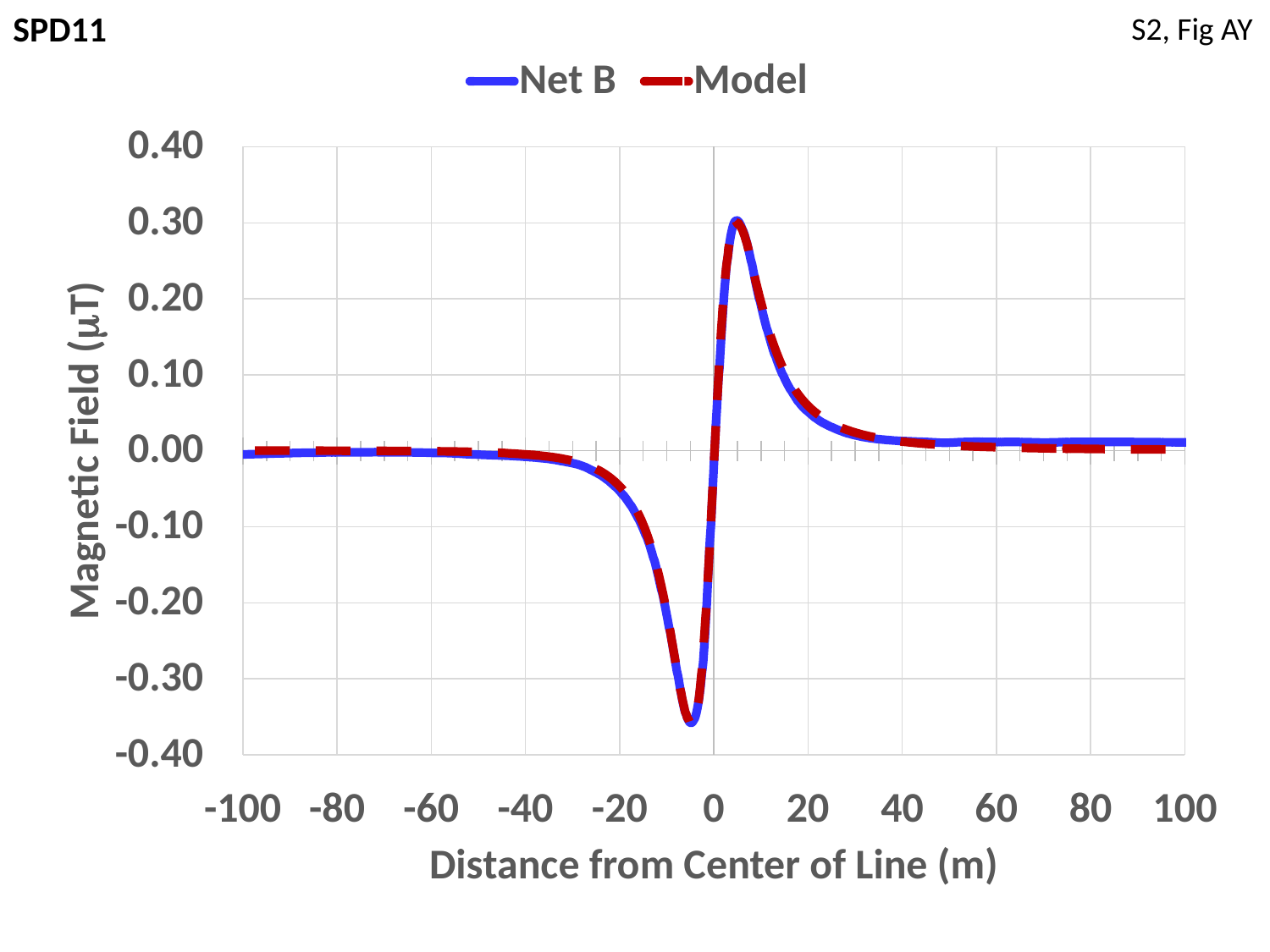

SPD11
S2, Fig AY

## Slide 53
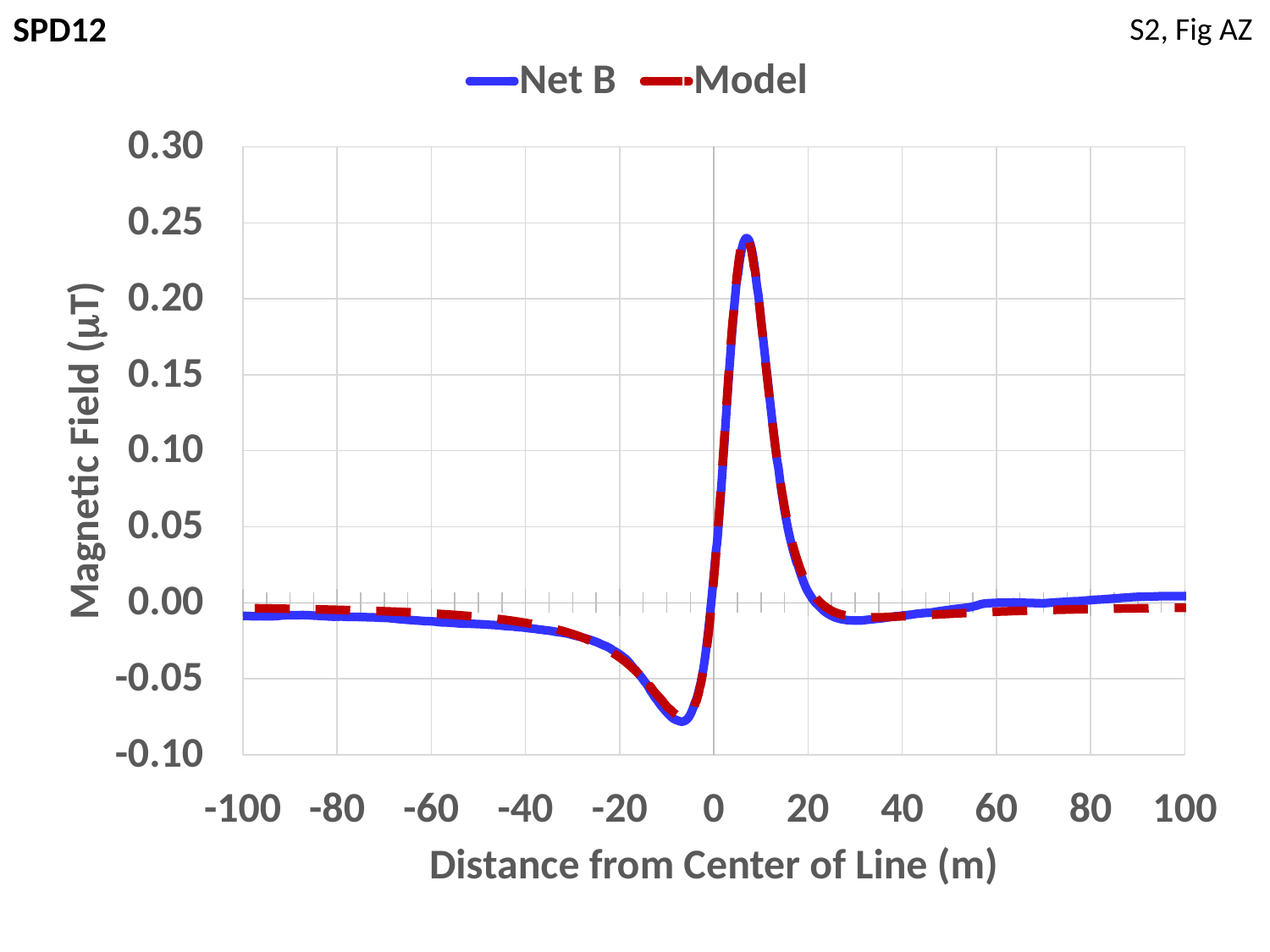

SPD12
S2, Fig AZ

## Slide 54
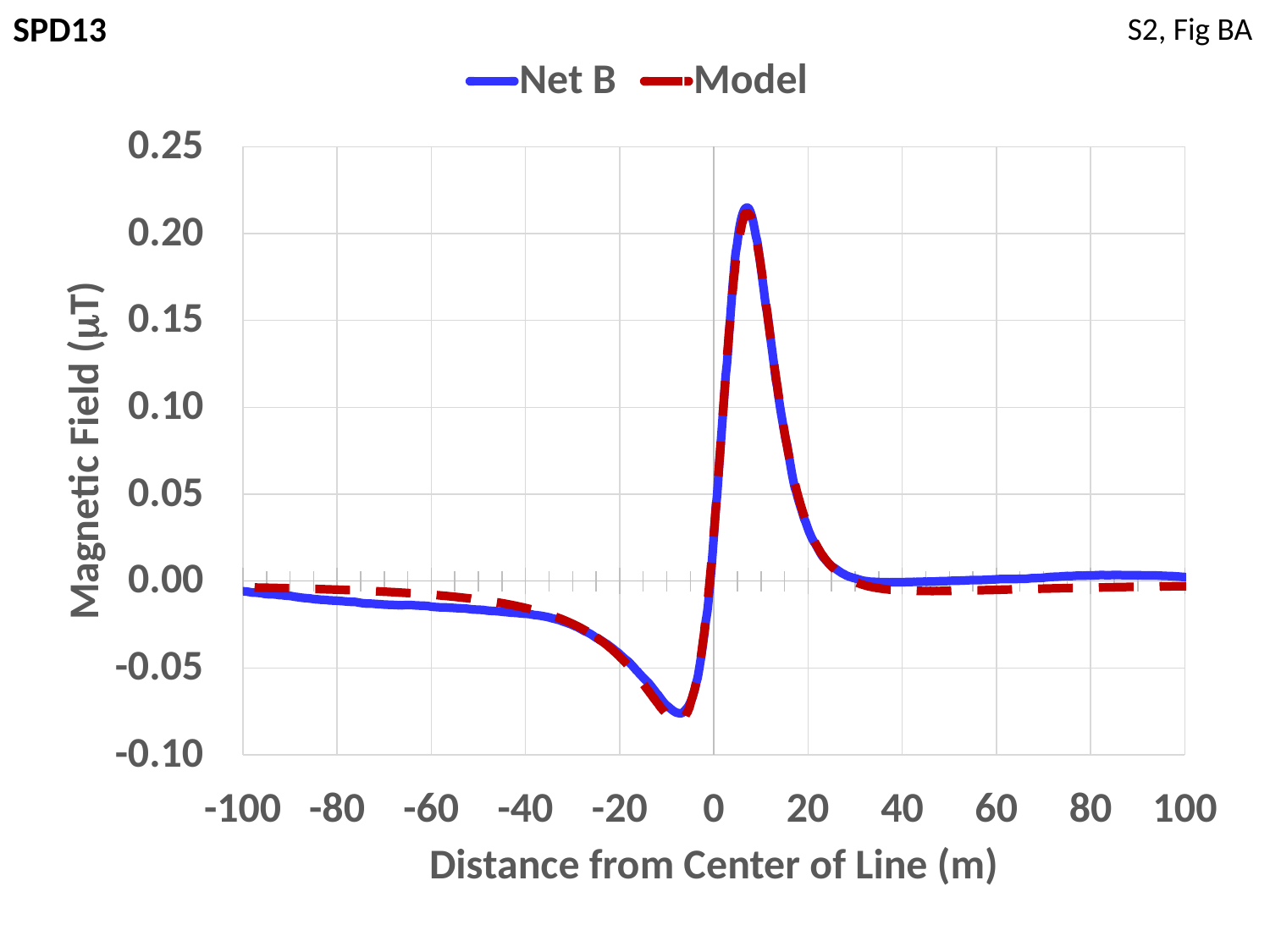

SPD13
S2, Fig BA

## Slide 55
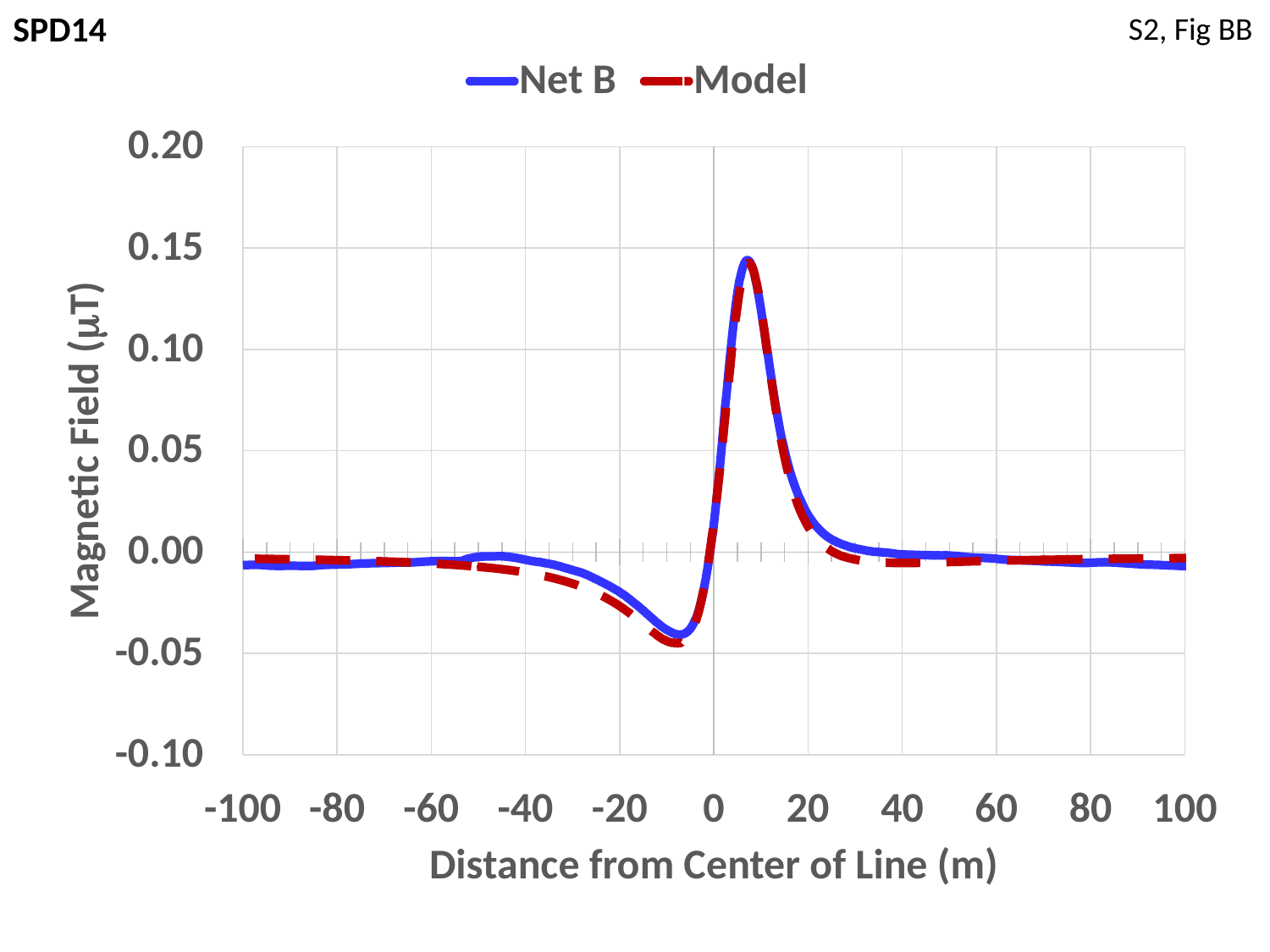

SPD14
S2, Fig BB

## Slide 56
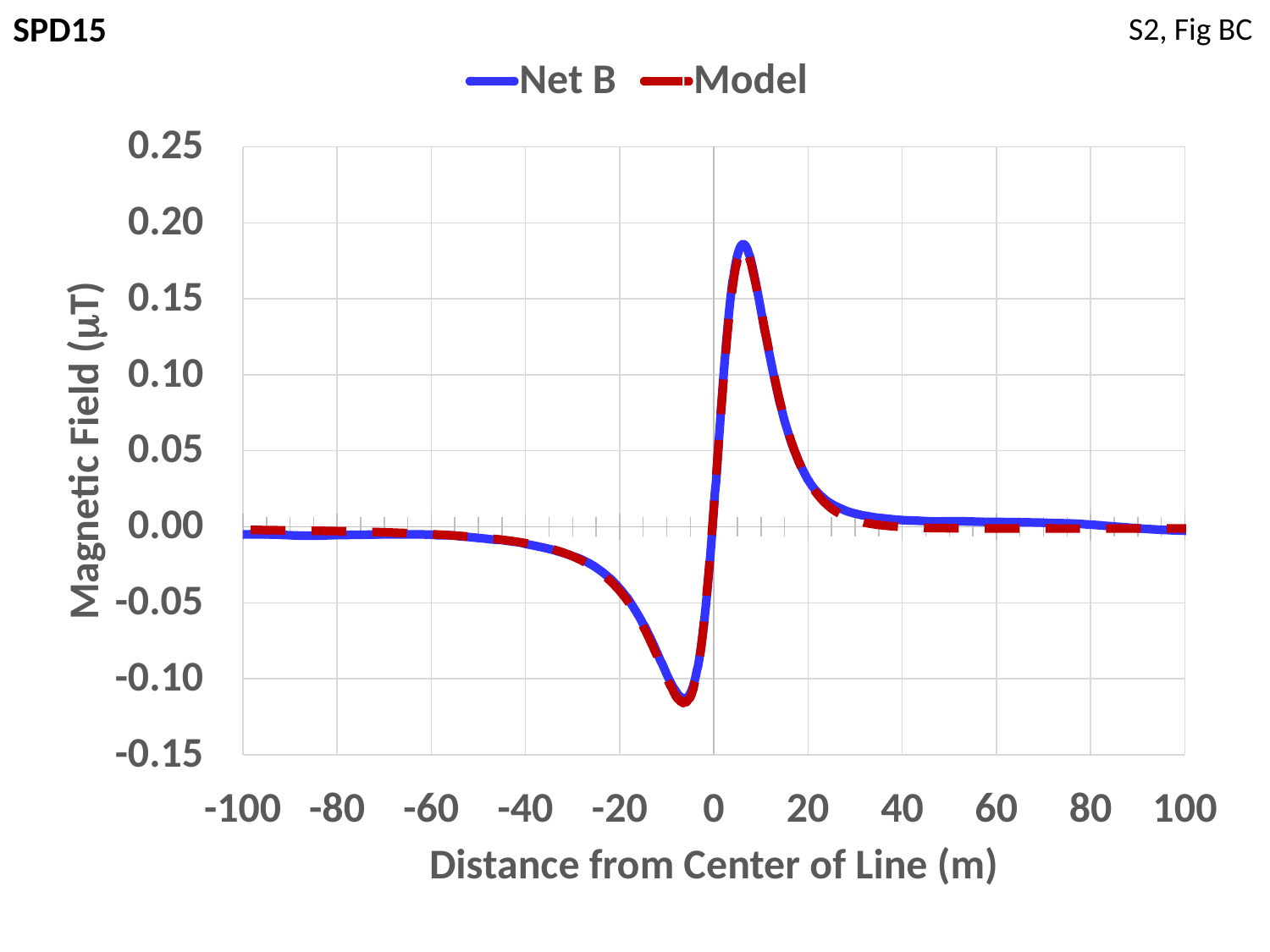

SPD15
S2, Fig BC

## Slide 57
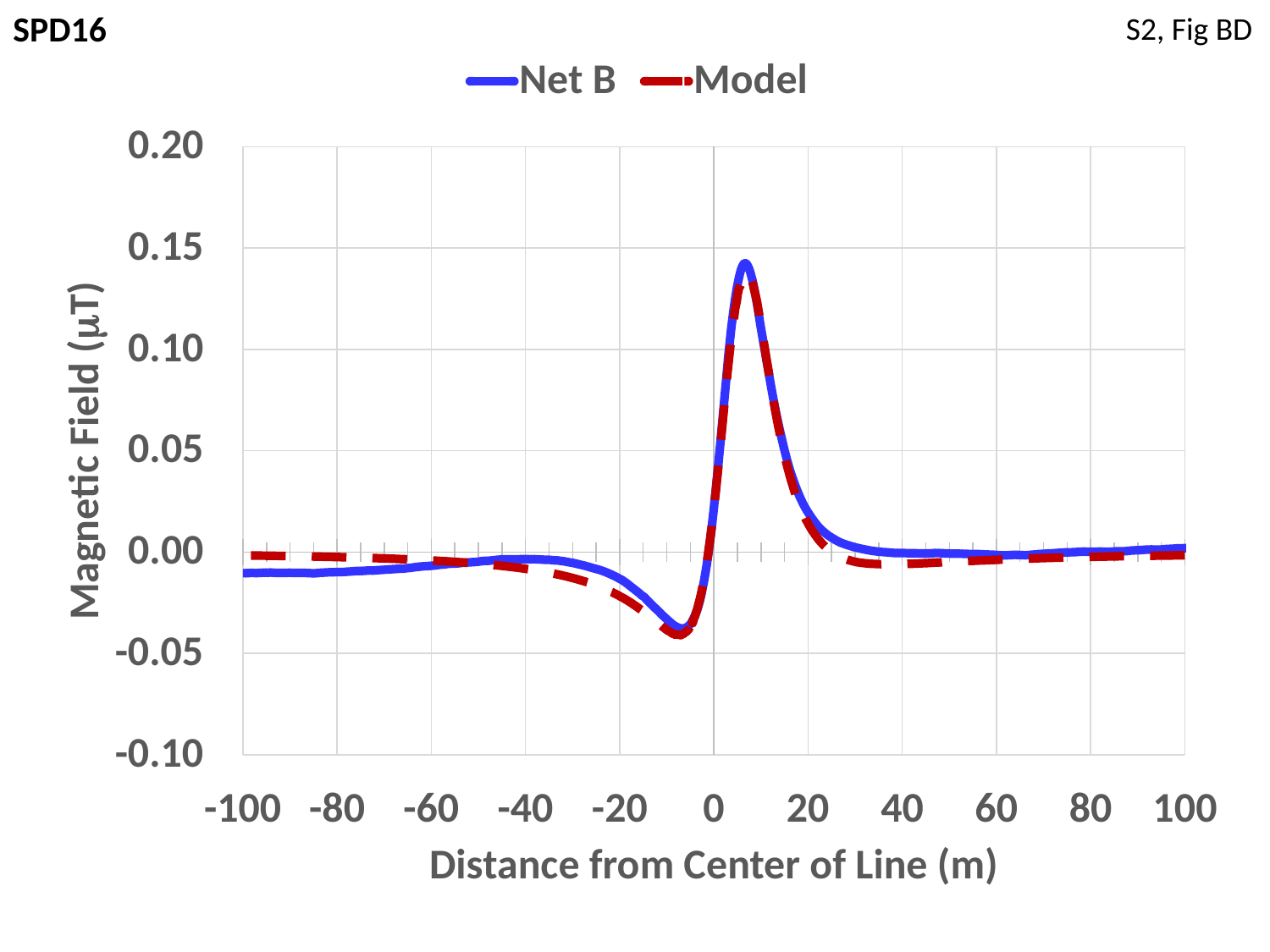

SPD16
S2, Fig BD

## Slide 58
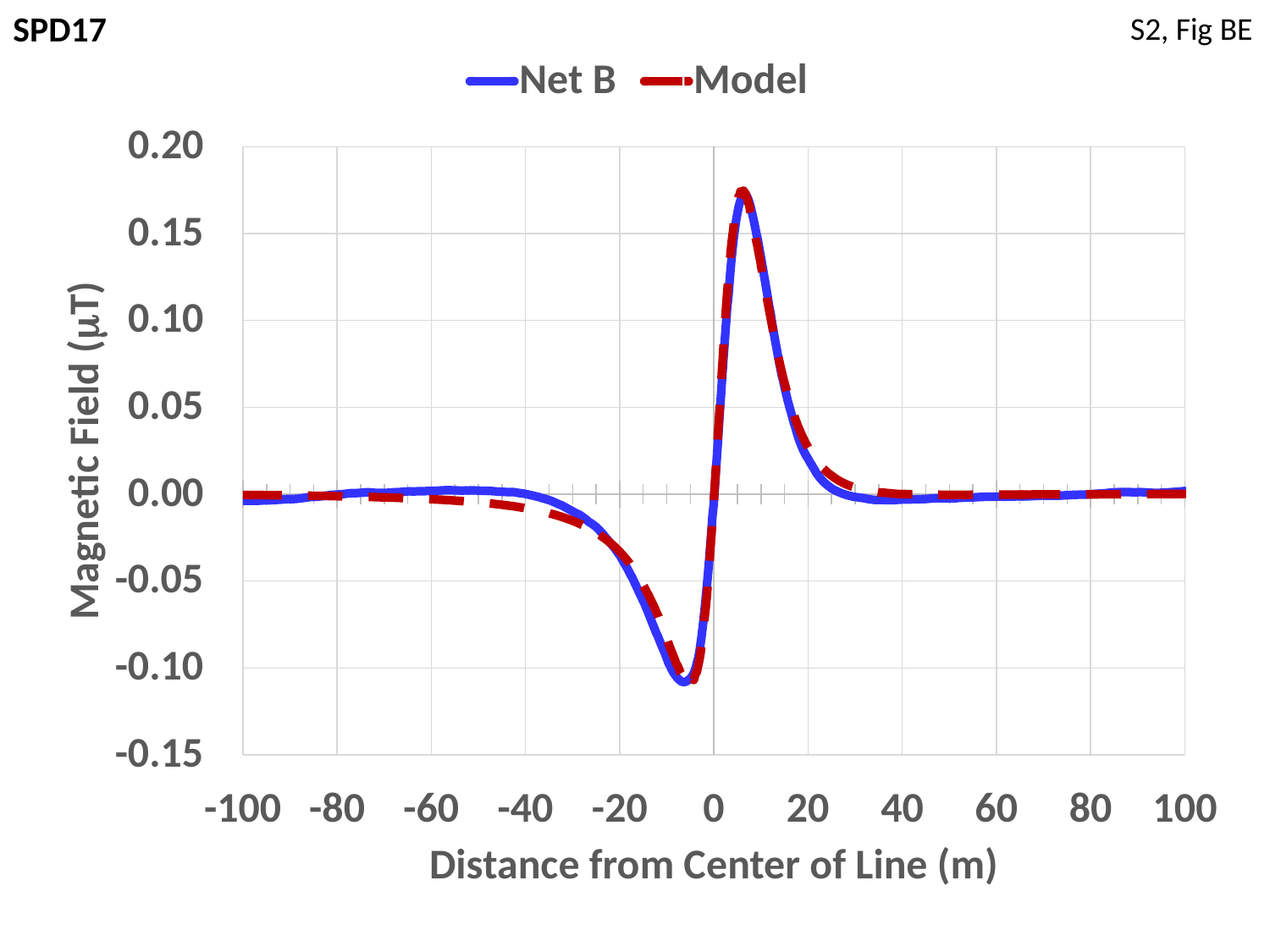

SPD17
S2, Fig BE

## Slide 59
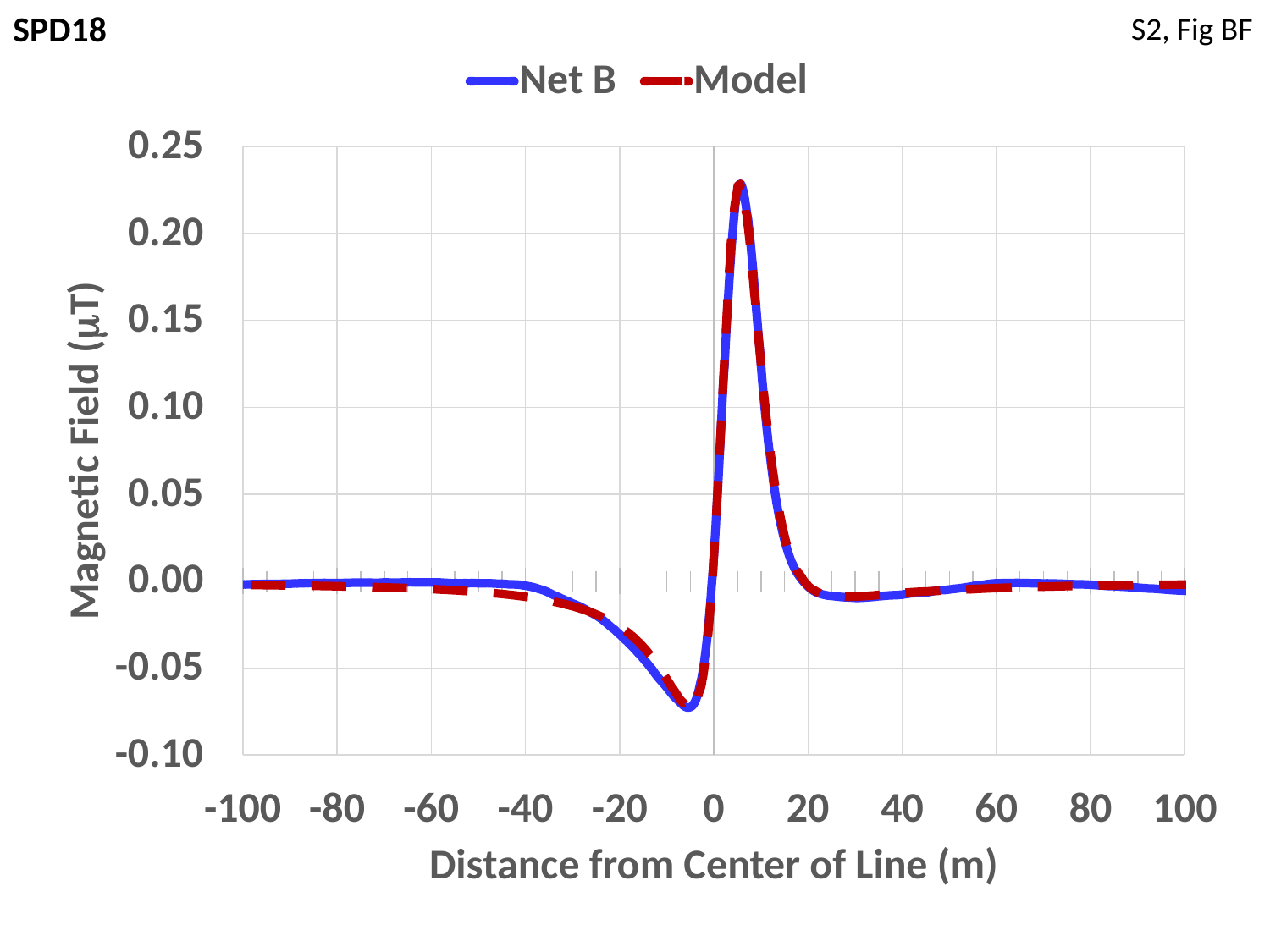

SPD18
S2, Fig BF

## Slide 60
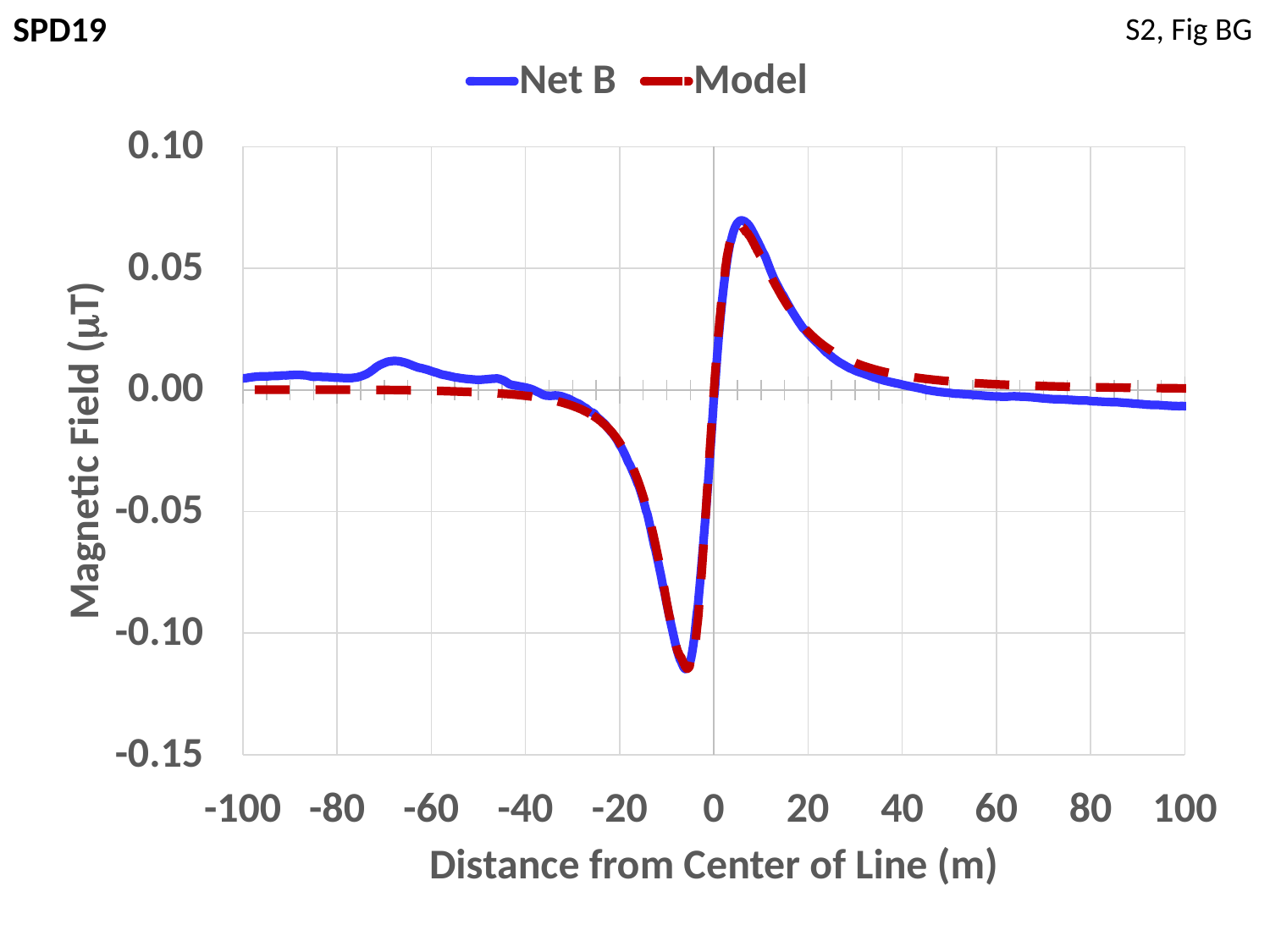

SPD19
S2, Fig BG

## Slide 61
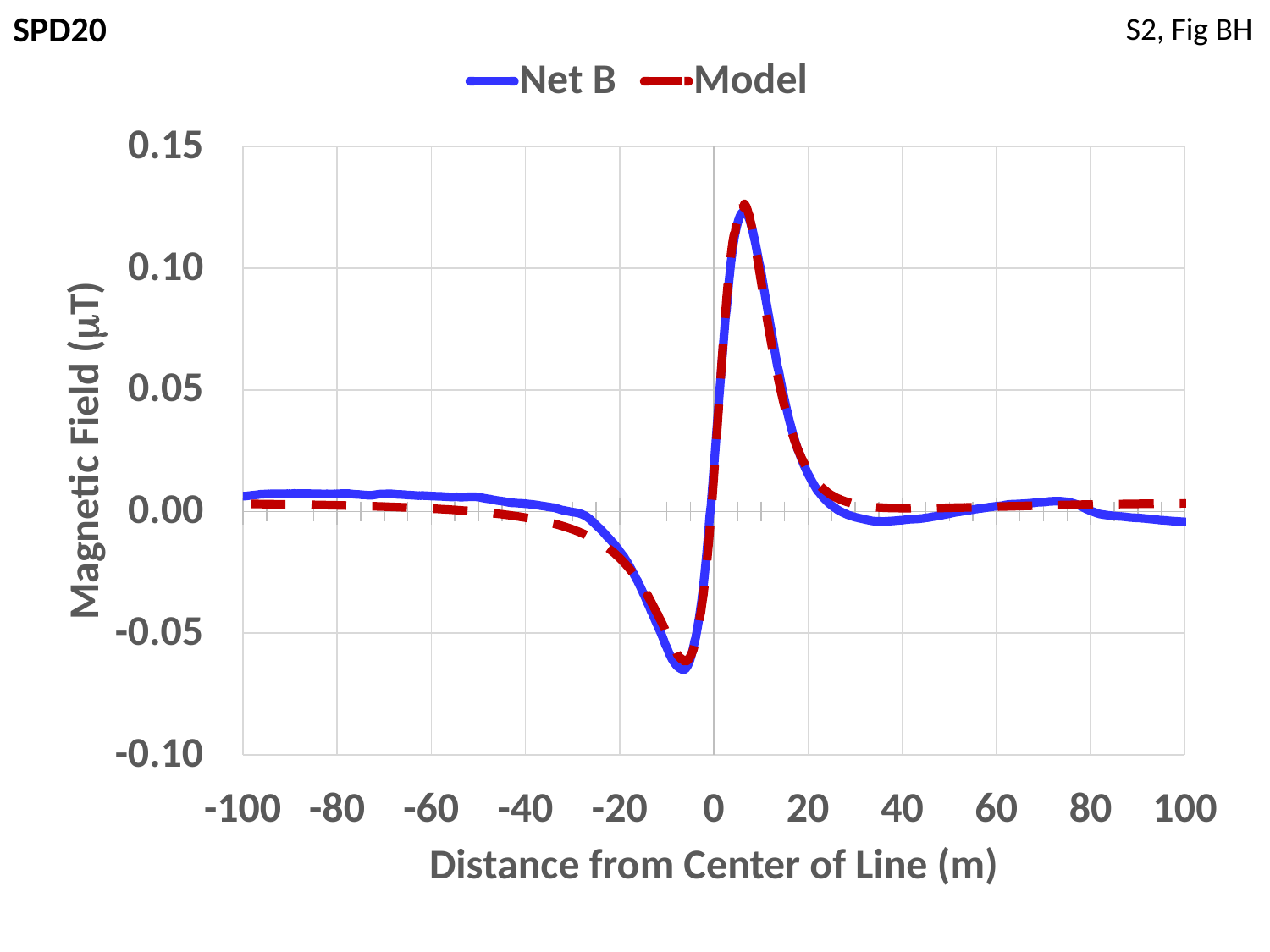

SPD20
S2, Fig BH

## Slide 62
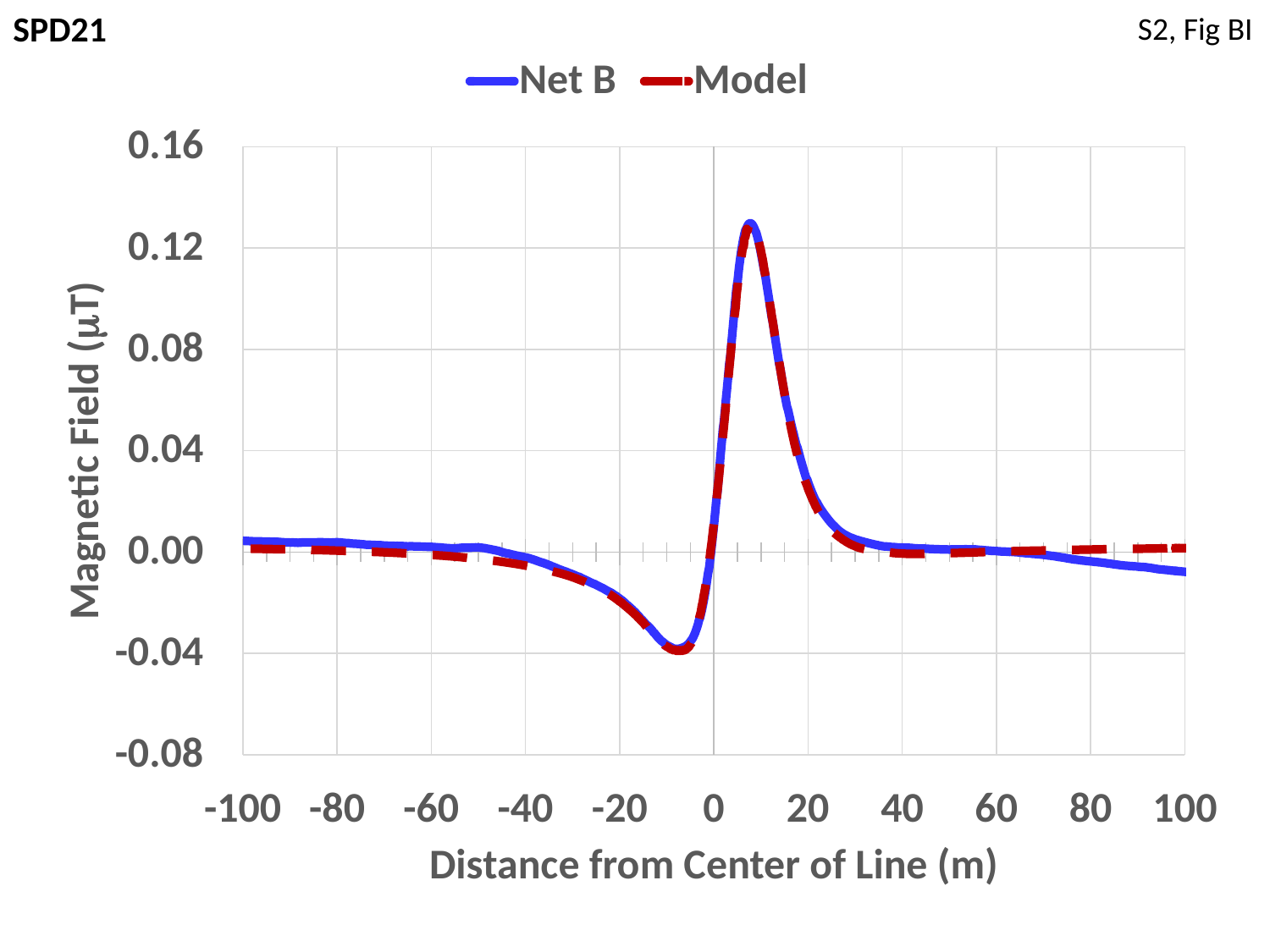

SPD21
S2, Fig BI

## Slide 63
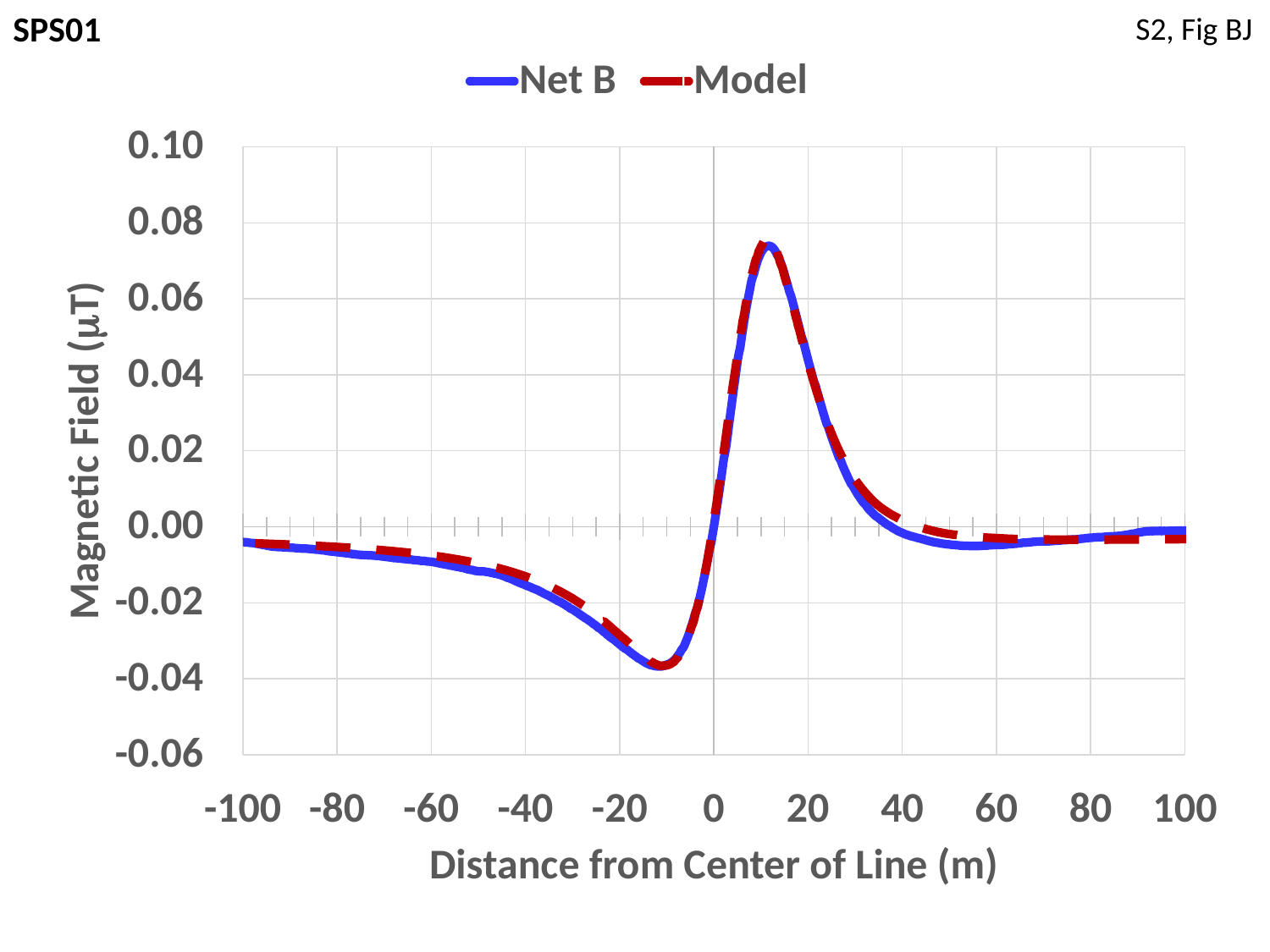

SPS01
S2, Fig BJ

## Slide 64
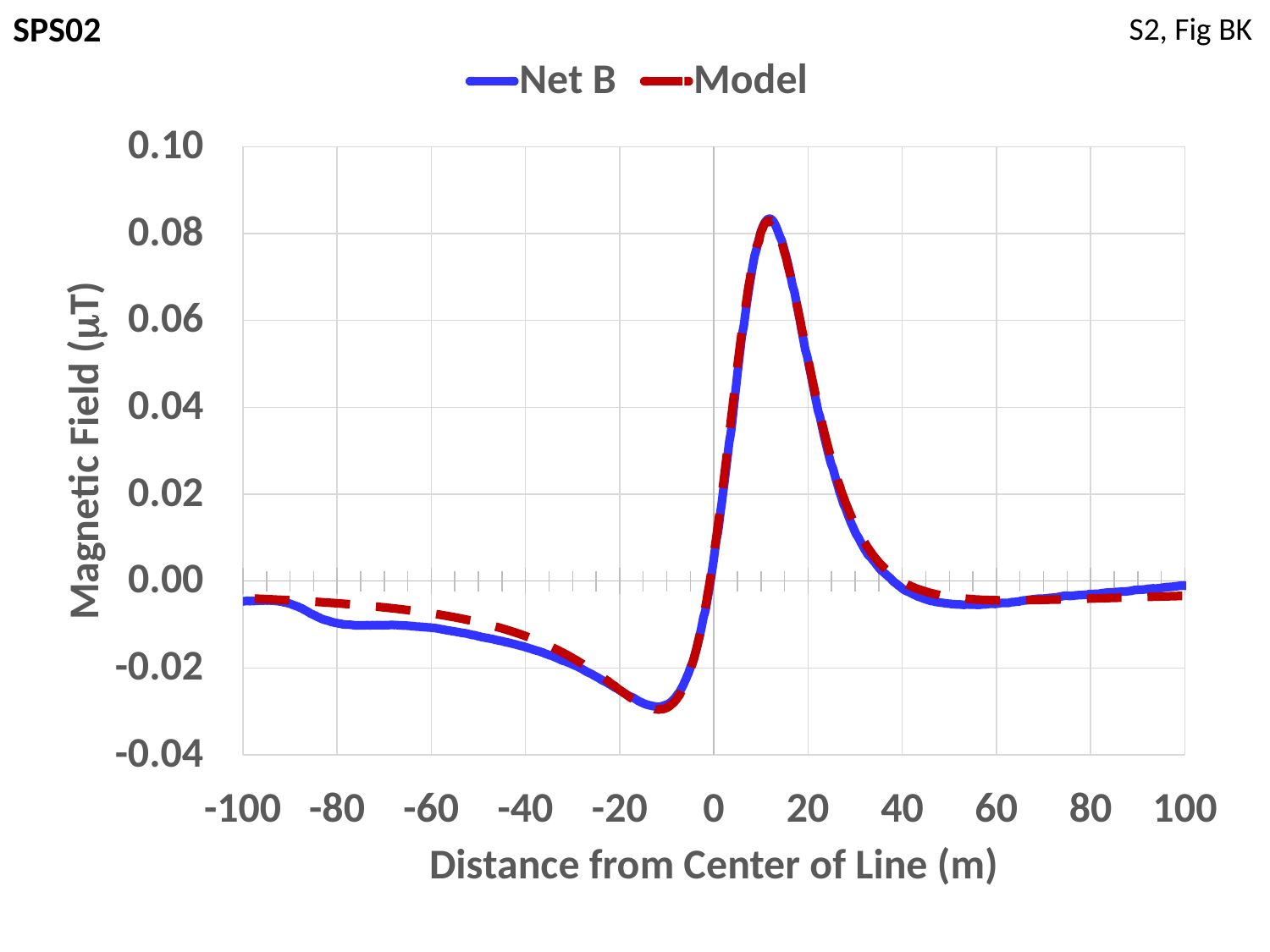

SPS02
S2, Fig BK

## Slide 65
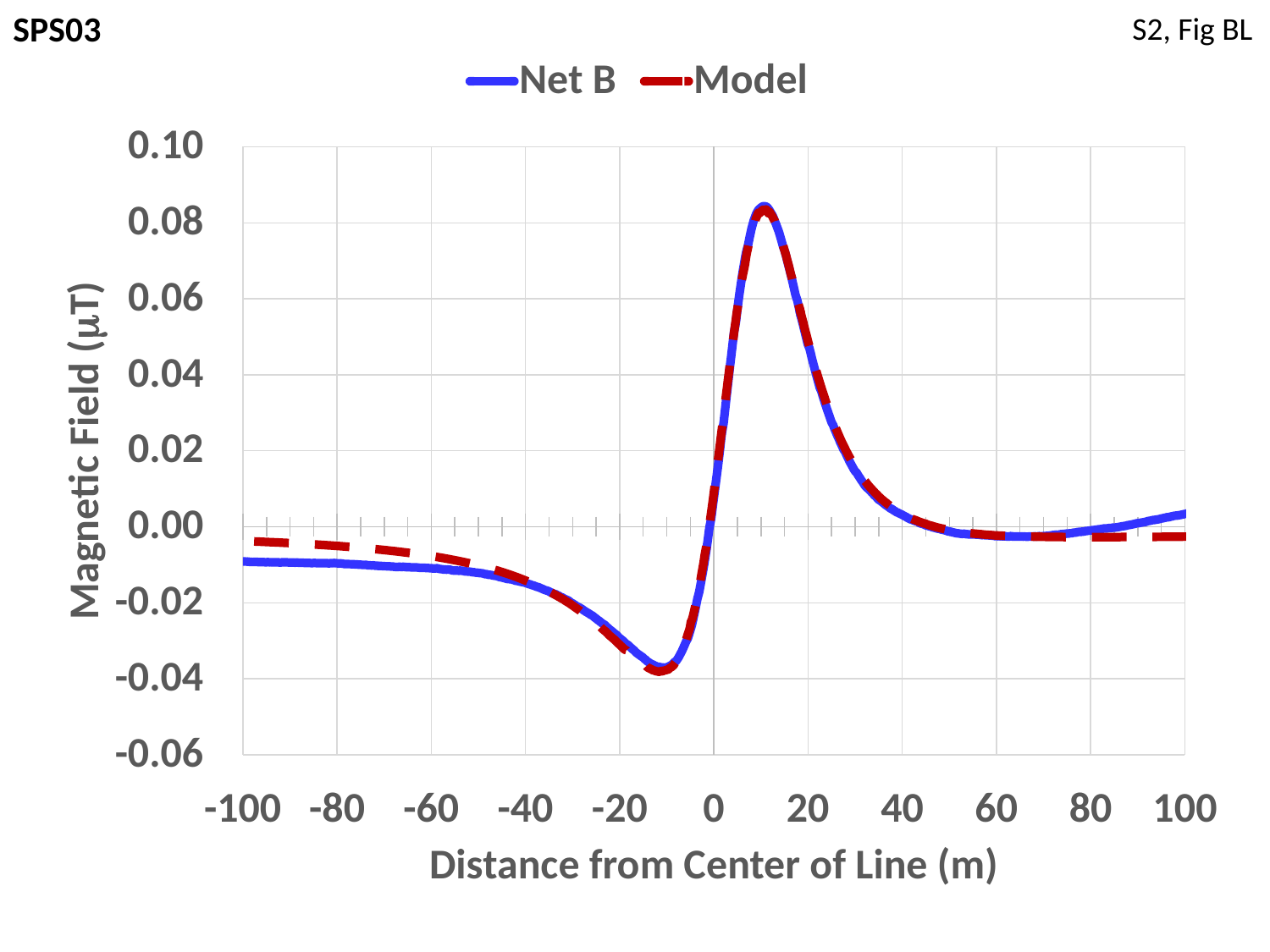

SPS03
S2, Fig BL

## Slide 66
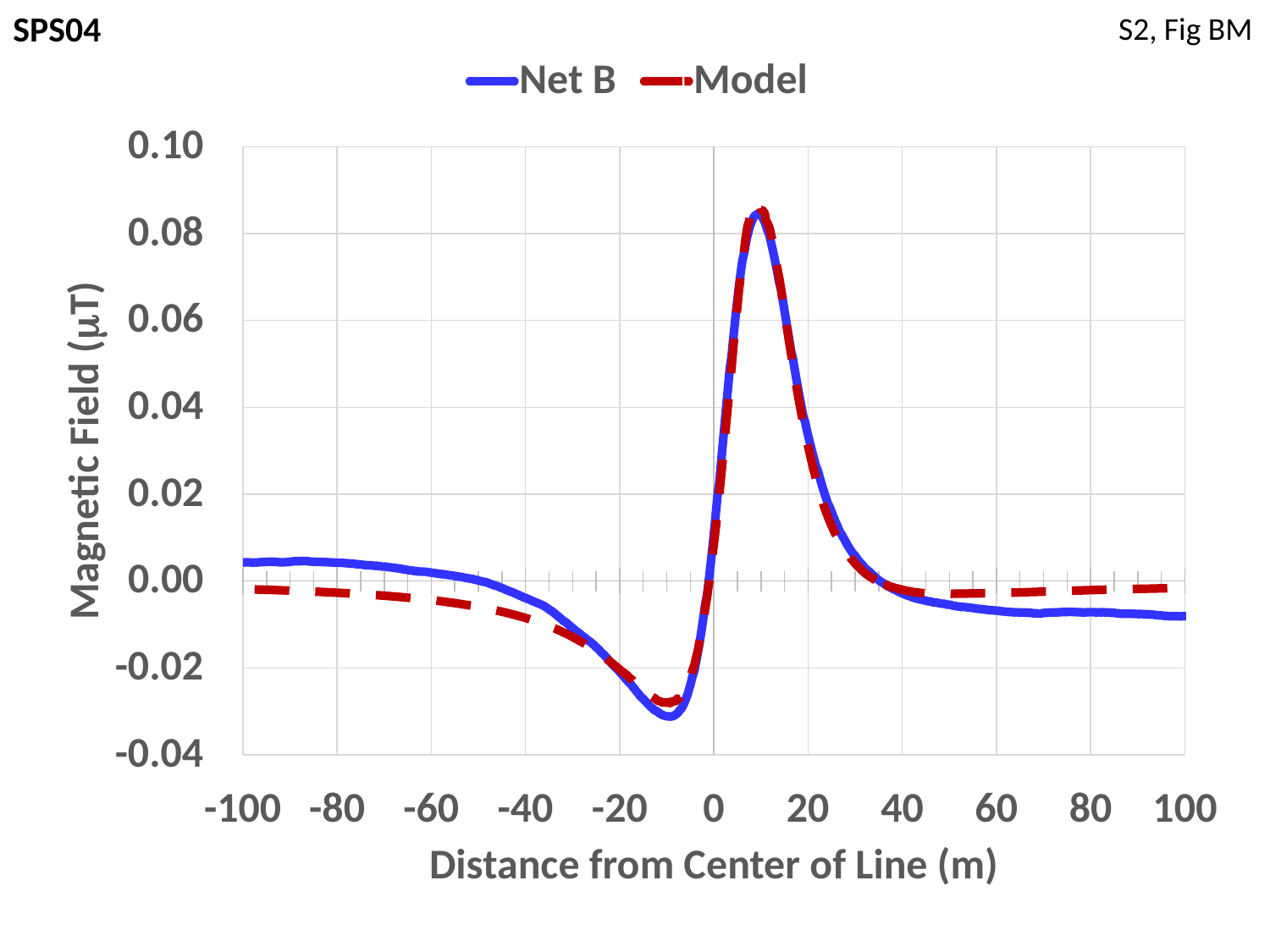

SPS04
S2, Fig BM

## Slide 67
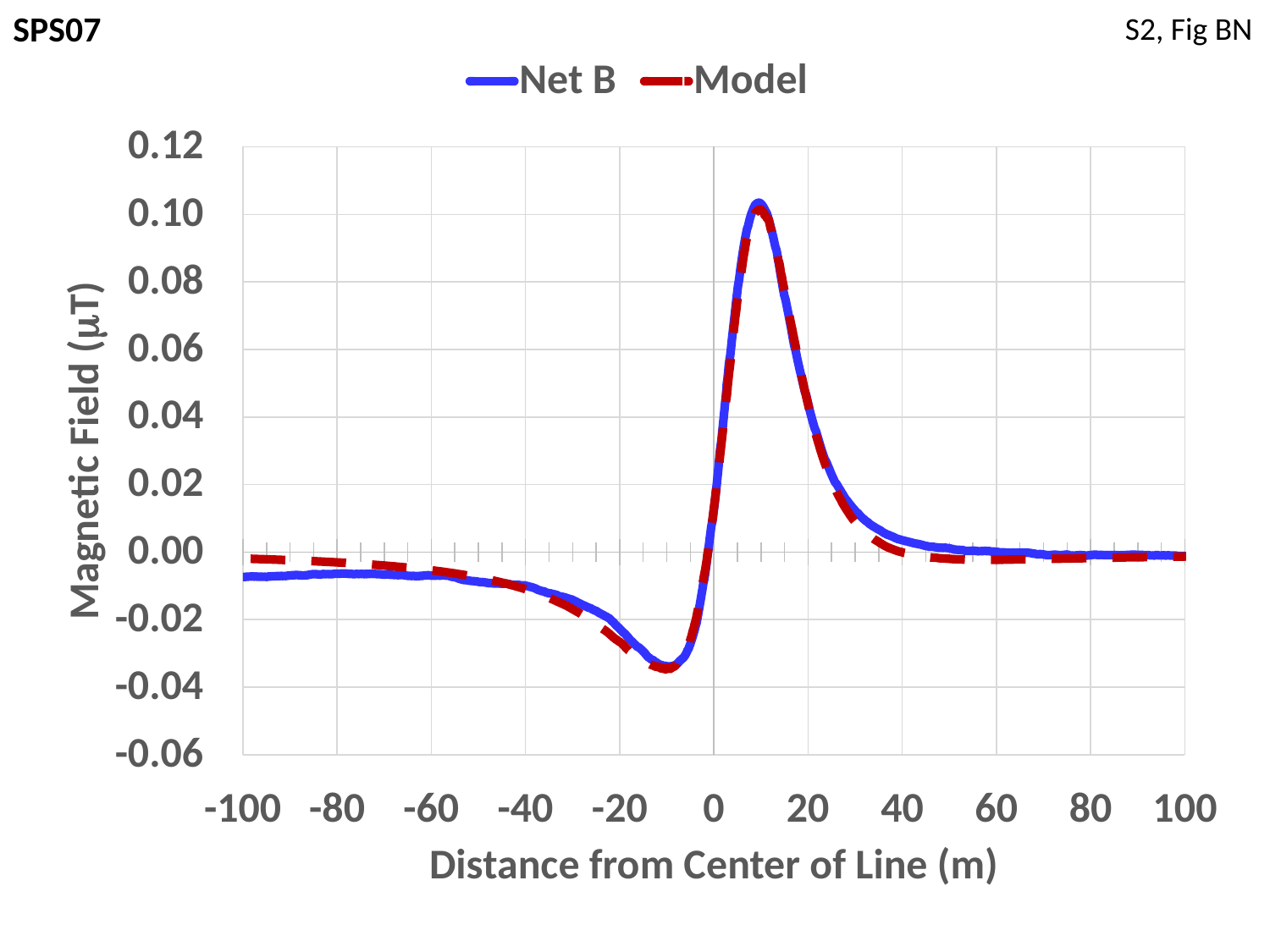

SPS07
S2, Fig BN

## Slide 68
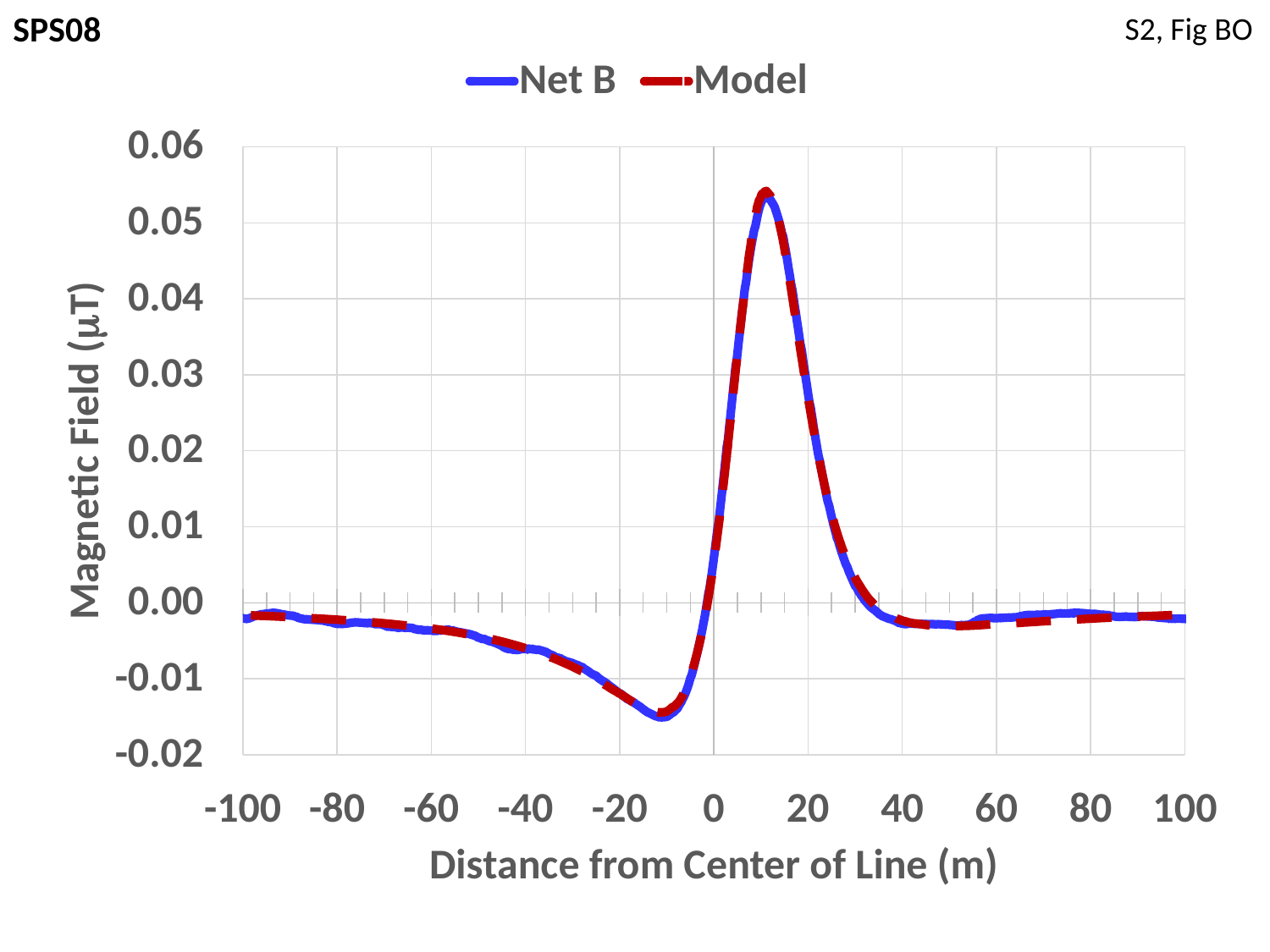

SPS08
S2, Fig BO

## Slide 69
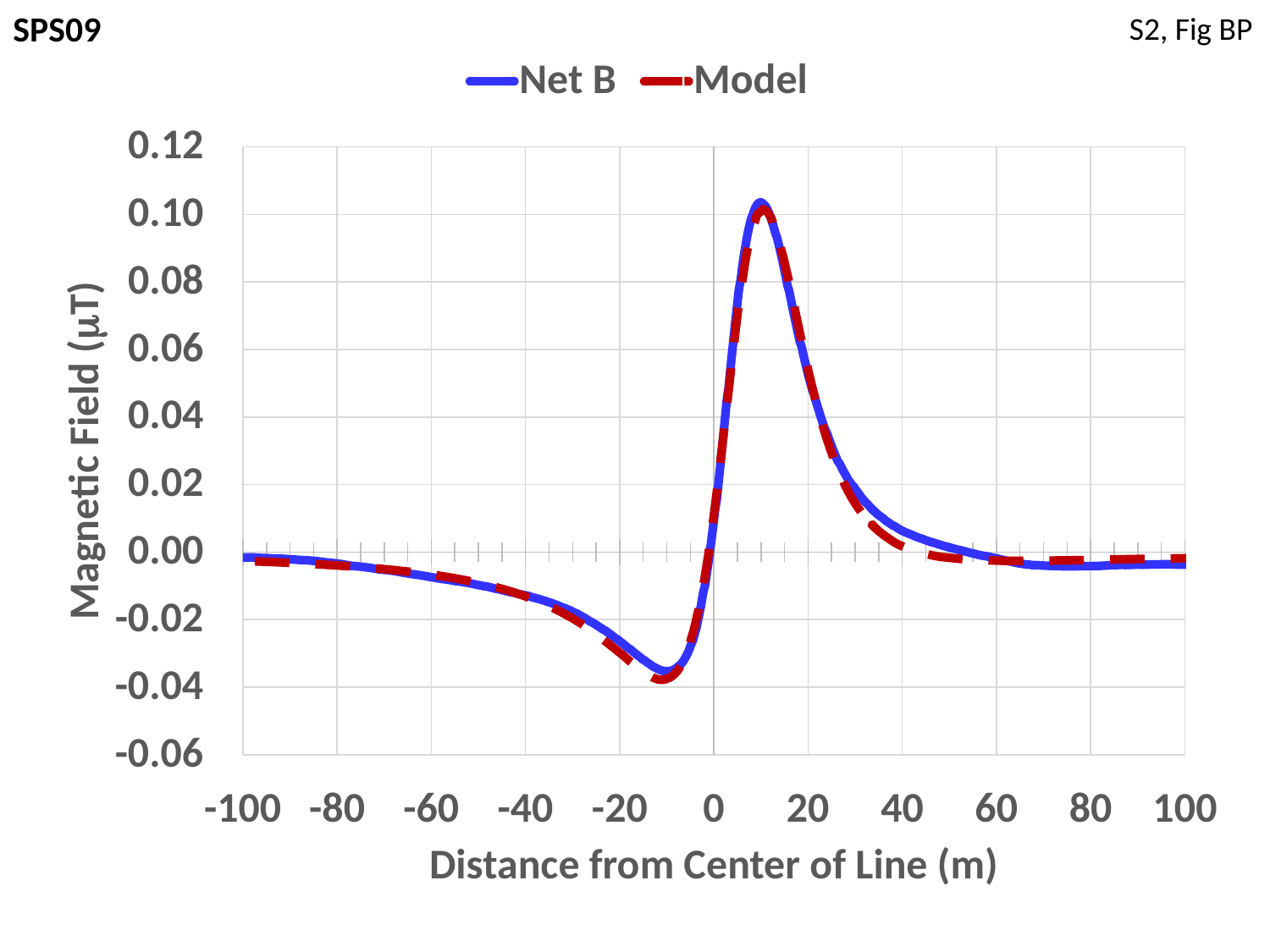

SPS09
S2, Fig BP

## Slide 70
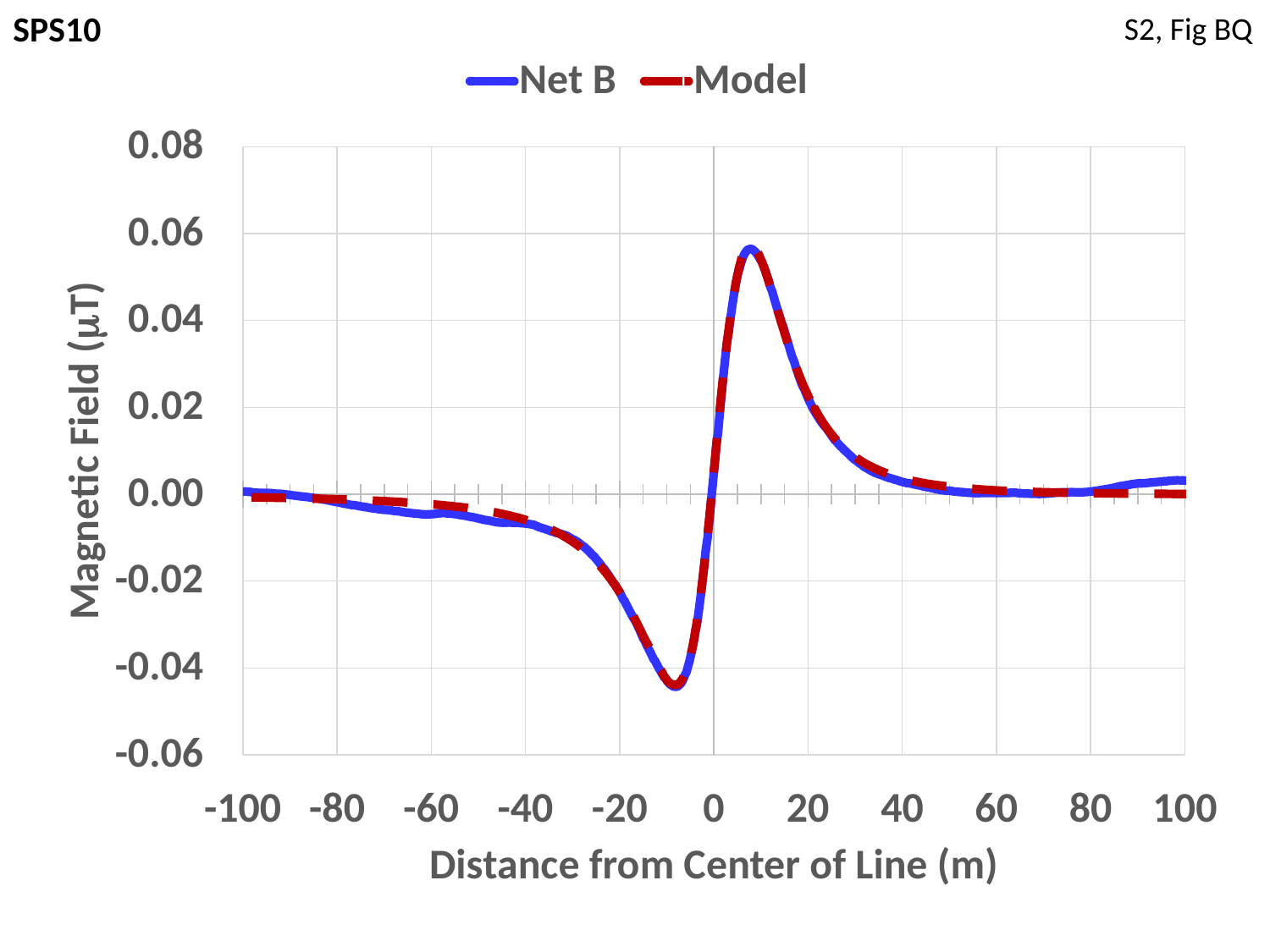

SPS10
S2, Fig BQ

## Slide 71
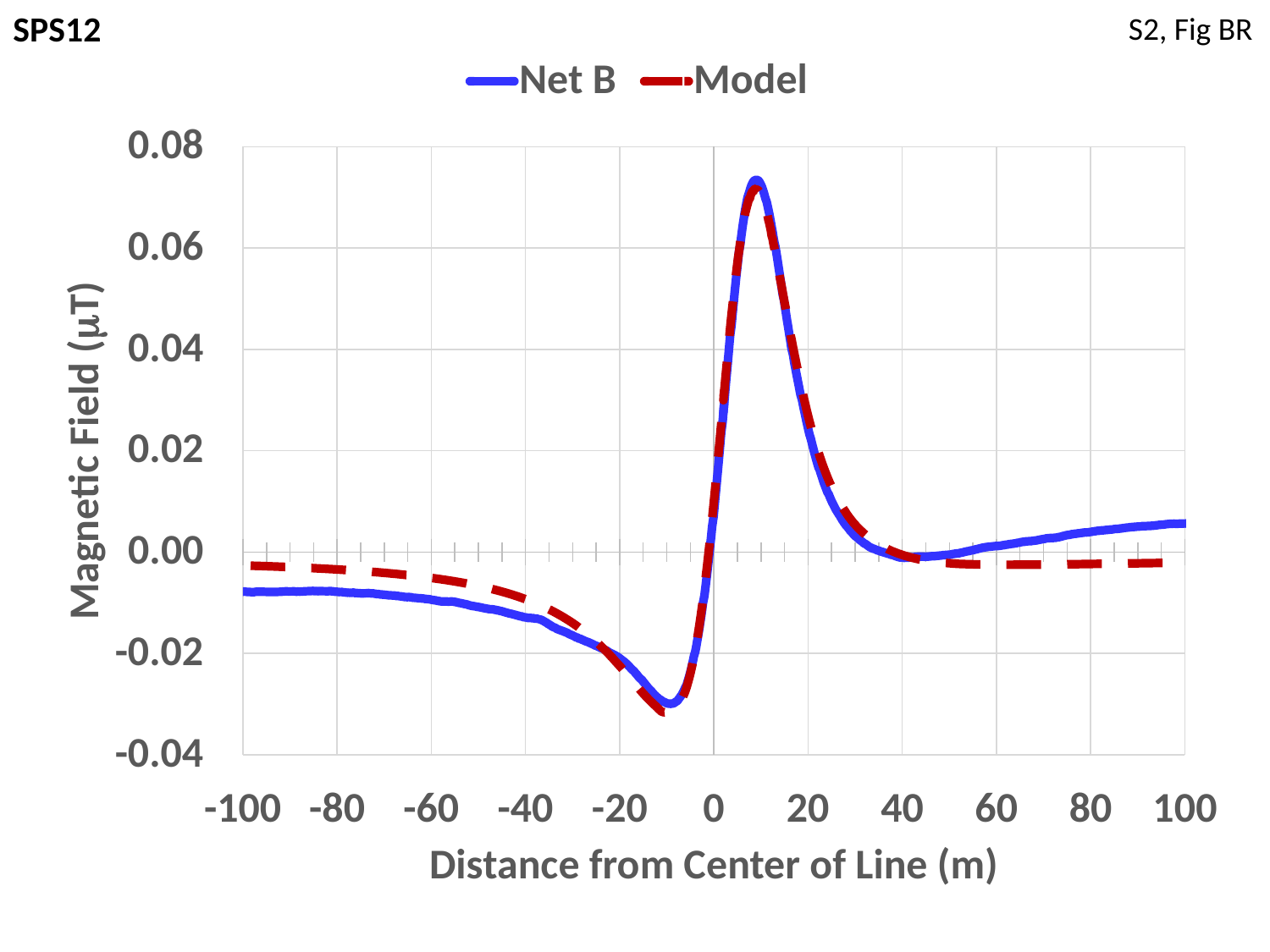

SPS12
S2, Fig BR

## Slide 72
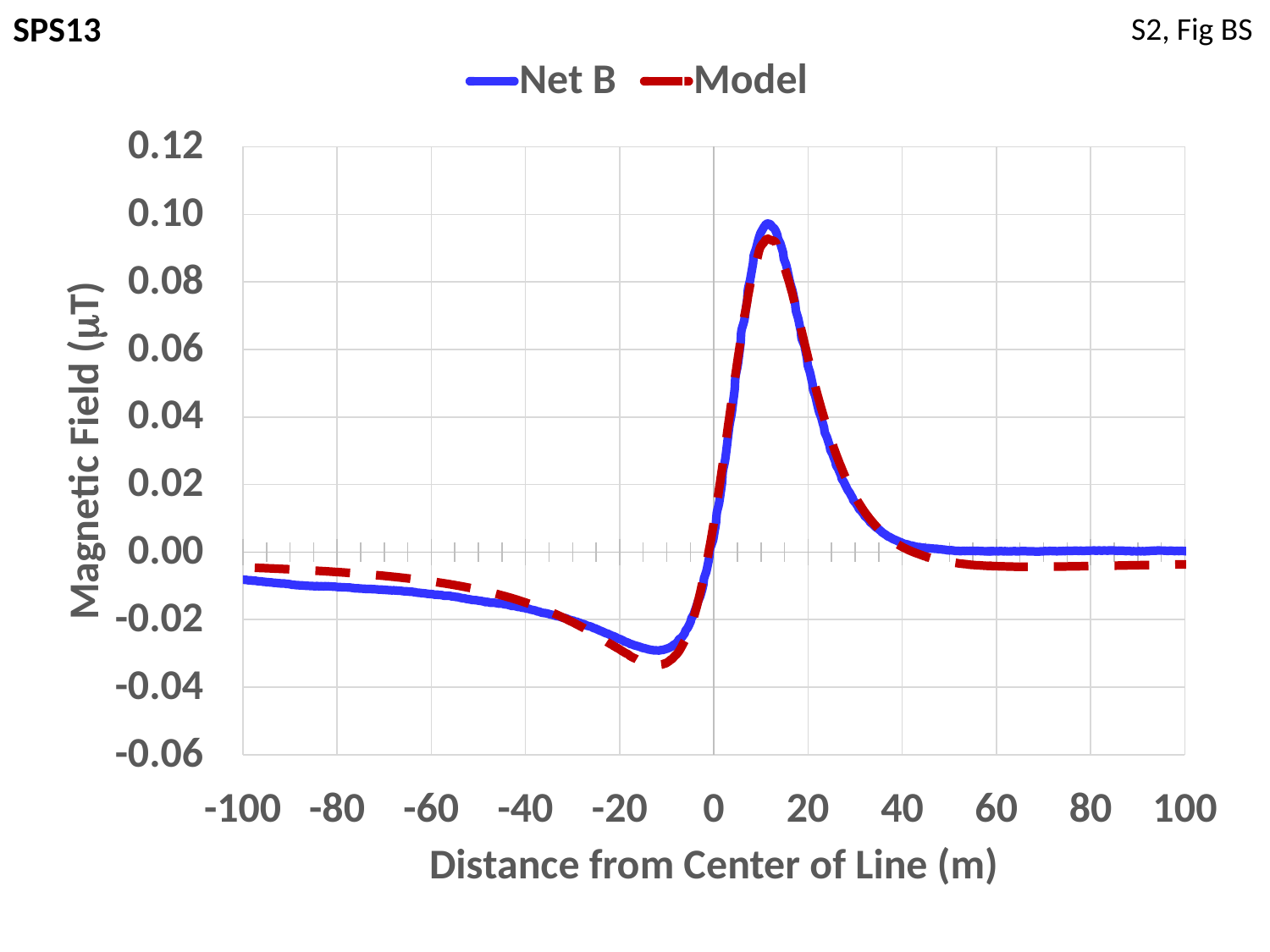

SPS13
S2, Fig BS

## Slide 73
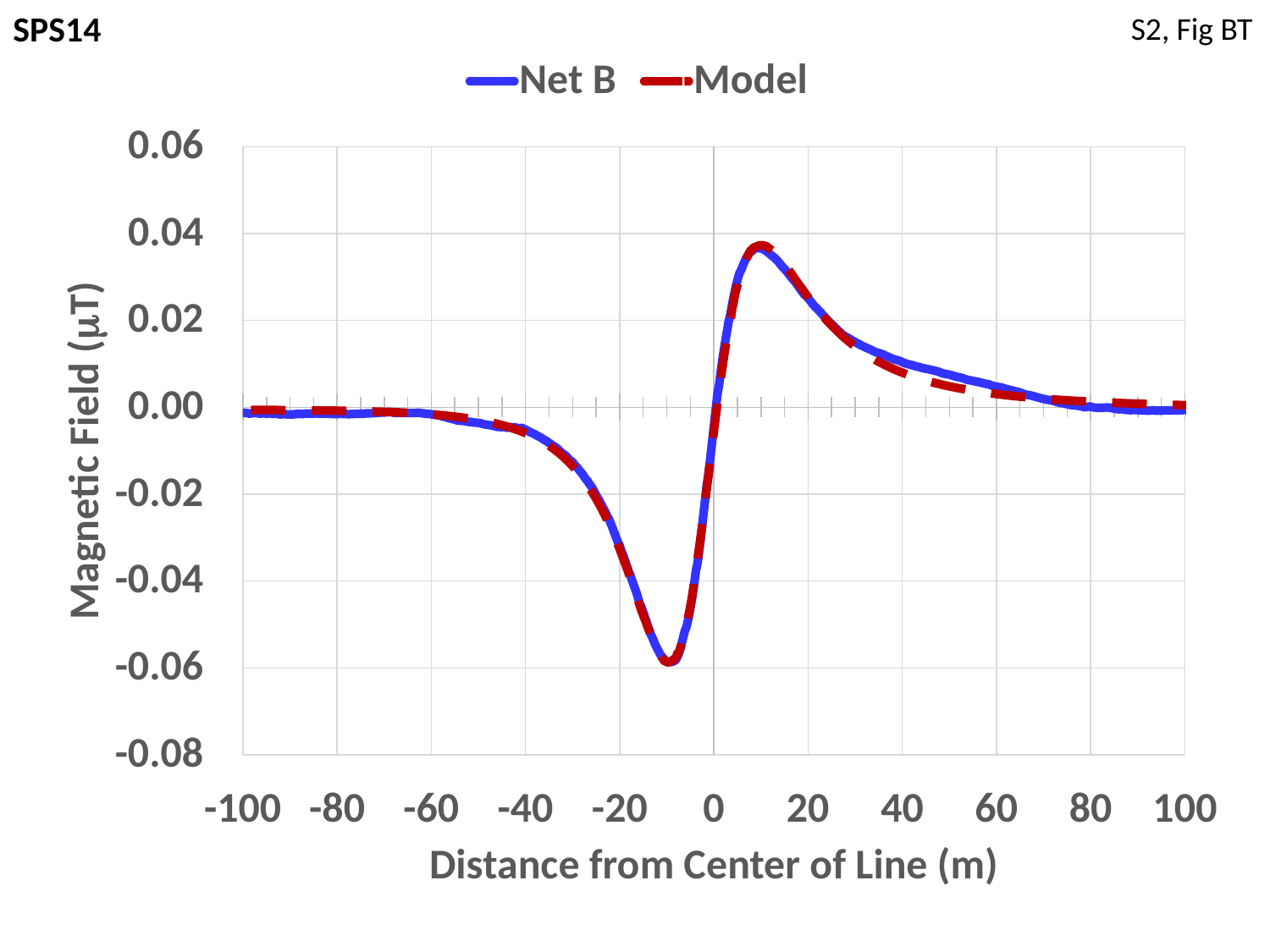

SPS14
S2, Fig BT

## Slide 74
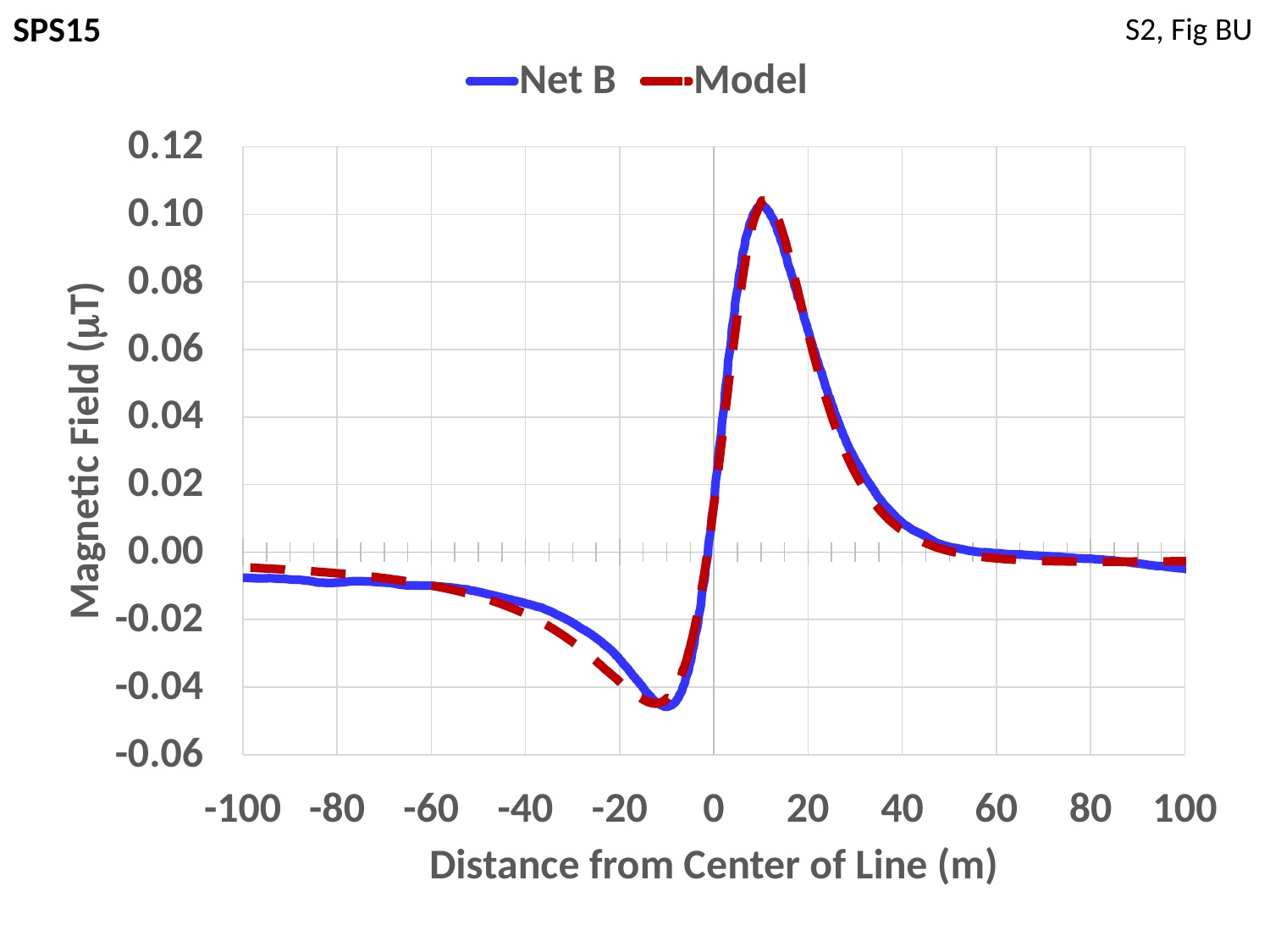

SPS15
S2, Fig BU

## Slide 75
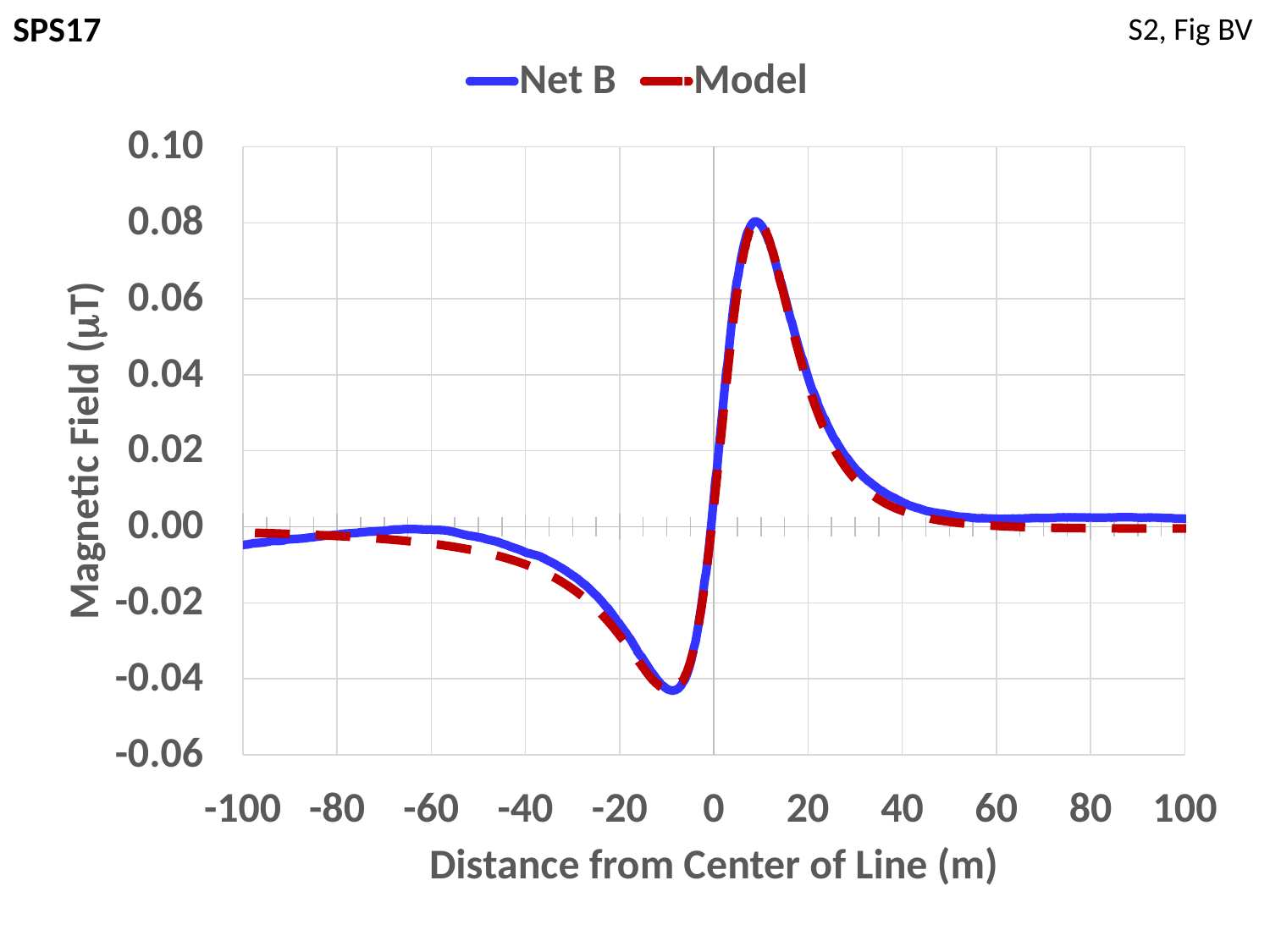

SPS17
S2, Fig BV

## Slide 76
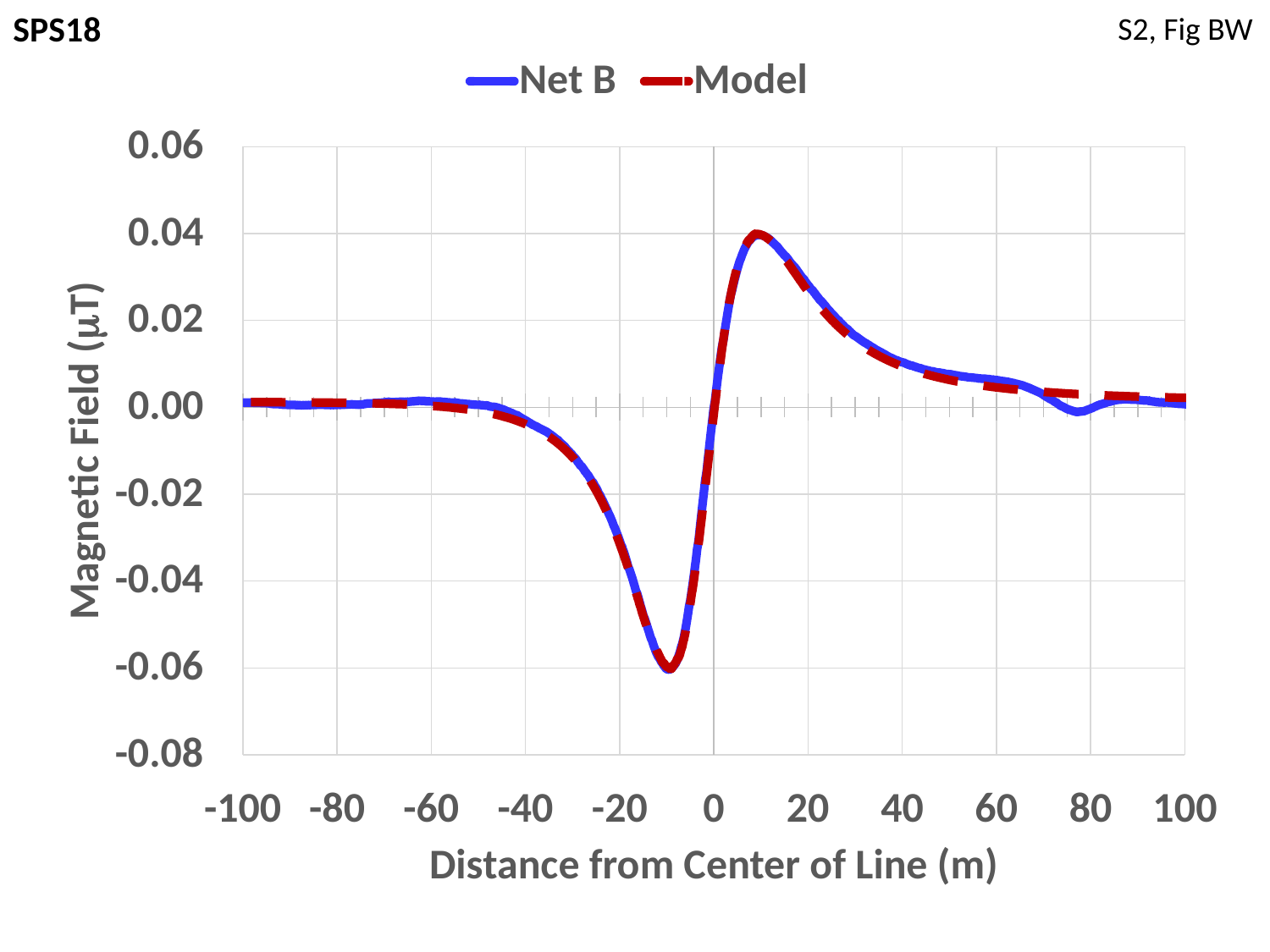

SPS18
S2, Fig BW

## Slide 77
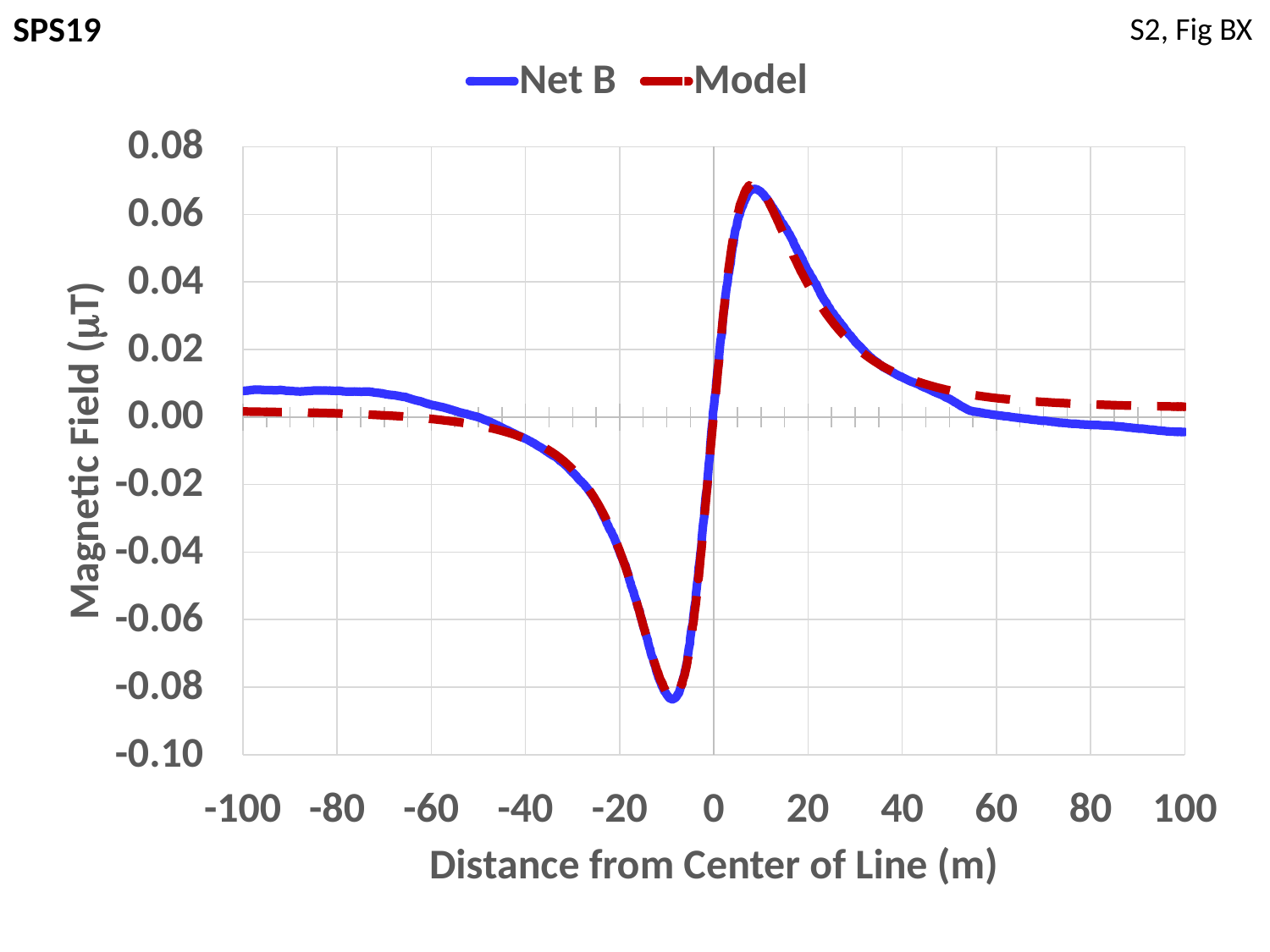

SPS19
S2, Fig BX

## Slide 78
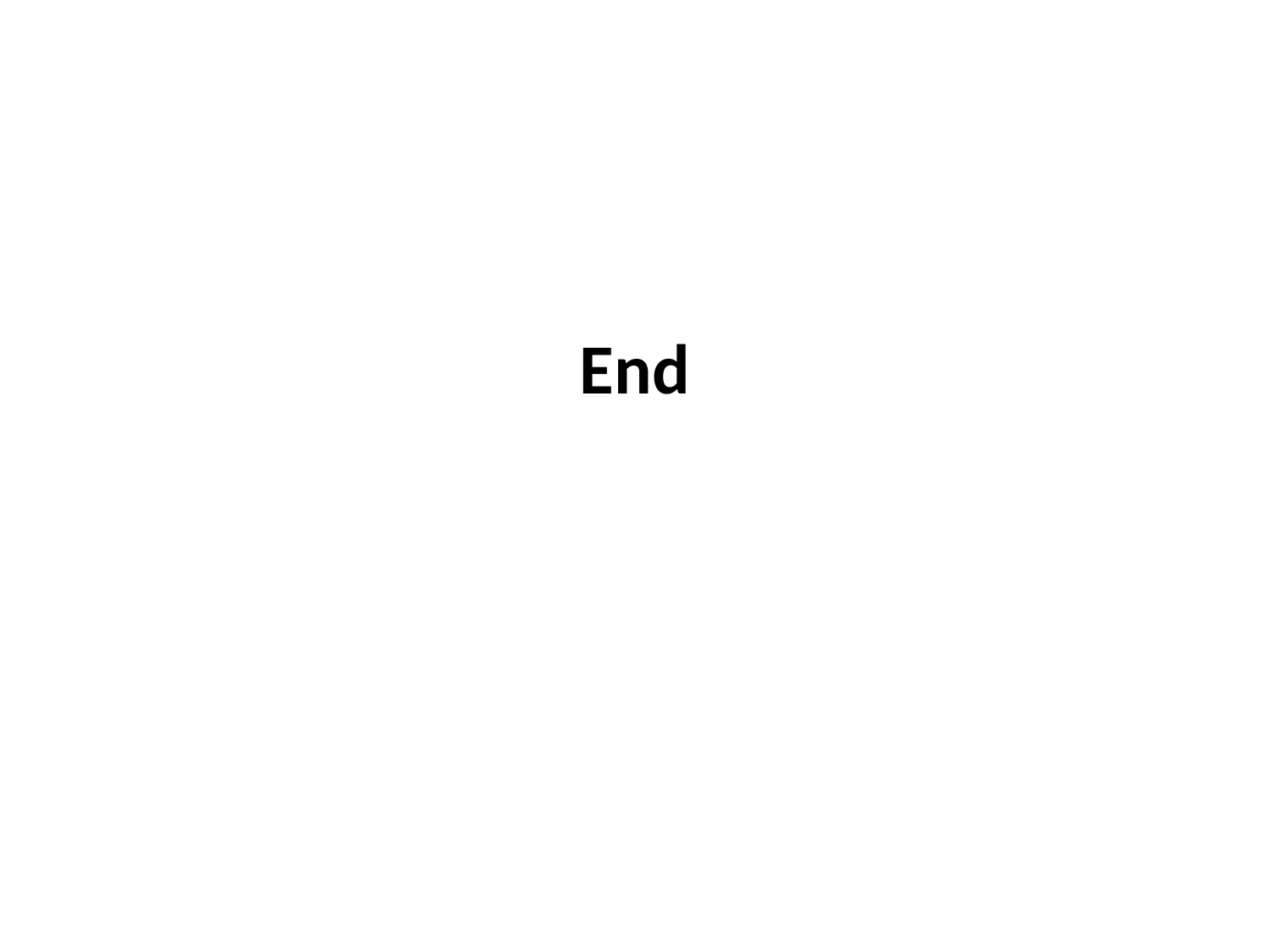

End
